# Bile-Acid-Appended Triazolyl Aryl Ketones: Design, Synthesis, In Vitro Anticancer Activity and Pharmacokinetics in Rats

**DOI:** 10.3390/molecules26195741

**Published:** 2021-09-22

**Authors:** Devesh S. Agarwal, Samrat Mazumdar, Kishan S. Italiya, Deepak Chitkara, Rajeev Sakhuja

**Affiliations:** 1 Department of Chemistry, Birla Institute of Technology and Science, Pilani 333 031, India; deveshagarwal88@gmail.com; 2 Department of Pharmacy, Birla Institute of Technology and Science, Pilani 333 031, India; samratm64@gmail.com (S.M.); italiyakishan2010@gmail.com (K.S.I.); deepak.chitkara@pilani.bits-pilani.ac.in (D.C.)

**Keywords:** bile acid, anticancer, cytotoxicity, apoptosis, pharmacokinetic study

## Abstract

A library of bile-acid-appended triazolyl aryl ketones was synthesized and characterized by detailed spectroscopic techniques such as ^1^H and ^13^C NMR, HRMS and HPLC. All the synthesized conjugates were evaluated for their cytotoxicity at 10 µM against MCF-7 (human breast adenocarcinoma) and 4T1 (mouse mammary carcinoma) cells. In vitro cytotoxicity studies on the synthesized conjugates against MCF-7 and 4T1 cells indicated one of the conjugate **6cf** to be most active against both cancer cell lines, with IC_50_ values of 5.71 µM and 8.71 µM, respectively, as compared to the reference drug docetaxel, possessing IC_50_ values of 9.46 µM and 13.85 µM, respectively. Interestingly, another compound **6af** (IC_50_ = 2.61 µM) was found to possess pronounced anticancer activity as compared to the reference drug docetaxel (IC_50_ = 9.46 µM) against MCF-7. In addition, the potent compounds (**6cf** and **6af**) were found to be non-toxic to normal human embryonic kidney cell line (HEK 293), as evident from their cell viability of greater than 86%. Compound **6cf** induces higher apoptosis in comparison to **6af** (46.09% vs. 33.89%) in MCF-7 cells, while similar apoptotic potential was observed for **6cf** and **6af** in 4T1 cells. The pharmacokinetics of **6cf** in Wistar rats showed an MRT of 8.47 h with a half-life of 5.63 h. Clearly, these results suggest **6cf** to be a potential candidate for the development of anticancer agents.

## 1. Introduction

Cancer is presently a major health concern around the globe, leading to an alarming increase in the number of deaths, after cardiovascular disease, which is further expected to elevate to 12 million by 2030, as per WHO report [1,2,3,4]. Among all, breast cancer is the second most treacherous and common form of malignant tumor found in 23% of all forms of female cancers [5,6]. Cancer treatment procedures, such as hormone and radiation therapy, immunotherapy, combination chemotherapy and surgery, have been implemented to achieve reasonable success in this battle of mankind against cancer [7]. Among these, chemotherapy has proved to be one of the most promising pathways to overcome cancer; however, concerns such as selectivity, resistance and bioavailability of existing chemotherapeutic agents and associated side effects limit its exemplification as an ideal cancer-treating procedure [8]. Thus, the search for selective anticancer agents with lower side effects and better efficacy remains a prime target of medicinal chemists around the globe.

In this realm, some of the endogenous steroids and secondary bile acids have proven their repute as valuable cytotoxic agents [9]. For example, tauroursodeoxycholic acid (TUDCA) and ursodeoxycholic acid (UDCA) have shown significant apoptotic effects on various cancer cell lines [10,11,12,13]. Ursodeoxycholic acid (UDCA) has exhibited remarkable cytotoxicity against human oral squamous carcinoma (HSC-3), cultured animal/human tumor cells and HepG2 human hepatoma cells in combination with doxorubicin and also prevented colorectal adenoma recurrence [14,15]. Bile acids have also served as handy tools for the prodrug approach. For example, dihydroartemisinin–bile-acid hybridization has resulted in enhancement of dihydroartemisinin anticancer activity [16]. Strikingly, considerable research has been fueled toward developing steroidal heterocycles, in view of their broad spectrum of biological activities and added advantage of hydrophobic steroidal behavior capable of interacting with cell membranes [17].

**Figure 1:**
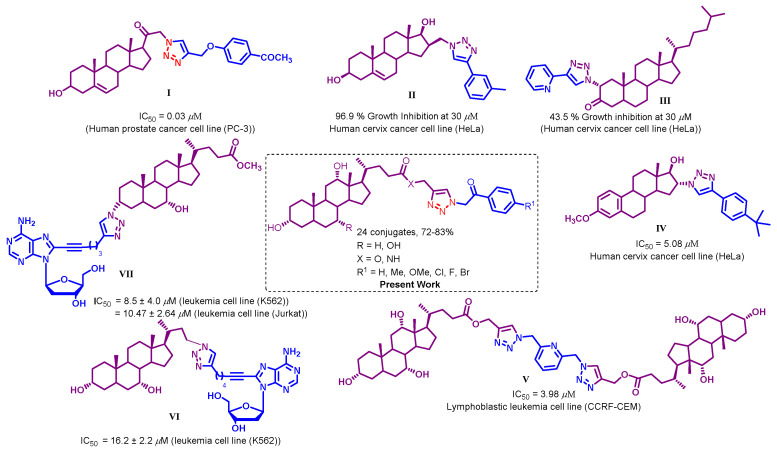
Representative examples of triazole-derived steroids and bile acids as anticancer agents.

In particular, 1,2,3-triazole provides favorable properties to binding molecular targets in a biological environment due to its metabolic stability and hydrogen-bonding capability [18]. Thus, the synthesis of diverse triazolyl steroids has received special interest due to a wide range of pharmacological properties such as anticancer [17,19] and antimicrobial [20,21]. Triazole-derived terpenoids/steroids such as oleanolic acid [22,23], betulinic acid [24,25,26,27,28], eteinic acid [29], nor-testosterone [30], androst-5-ene [31], estrone [29,32], 5α-androstane [33,34], cholesterol [20,21,35], 2-methoxyestradiol [36] and pregnenanes [37] have showcased interesting cytotoxic behavior against a variety of cancer cell lines (Figure 1, **I**–**IV**). However, bile-acid-appended triazoles have rarely been explored for their cytotoxic efficacy [38]. Drasar and coworkers documented one such report that discloses the synthesis and cytotoxicity of two ribbon-type pyridyl-based triazolyl cholic acid dimers, of which the ester analog demonstrated significant cytotoxic activity in low micromolar concentrations against lymphoblastic (CCRF-CEM) and myeloid leukemia (K562) cell lines (Figure 1, **V**) [29]. Perrone’s group reported cytotoxic studies on C-24-triazolyl-linked bile-acid–nucleoside conjugates, where a chenodeoxycholic-acid-linked deoxyadenosine derivative exhibited an IC_50_ value of 16.2 ± 2.2 µM against leukemia cell line (K562) (Figure 1, **VI**) [39]. The same group reported the synthesis of triazolyl-linked bile-acid–deoxyadenosine conjugates and evaluated their cytotoxic activity against two leukemia cell lines (Jurkat and K562), colon cancer cell line (HCT116), ovarian cancer cell line (A2780) and human fibroblast cell line. The best compound in this series exhibited an IC_50_ value of 8.51 ± 4.05 µM and 10.47 ± 2.64 µM against two leukemia cancer cell lines K562 and Jurkat, respectively (Figure 1, **VII**) [40].

In continuation to our program for the synthesis of C-24-functionalized bile acids as anticancer agents [41,42,43] and the aforementioned properties of triazolyl steroids, we present a synthetic approach for the synthesis of bile-acid-appended triazolyl aryl ketones. Their cytotoxic potency was examined against two breast cancer cell lines (MCF-7 and 4T1). In addition, in vivo pharmacokinetic study was also performed.

## 2. Results and Discussion

### 2.1. Chemistry

**Scheme 1:**
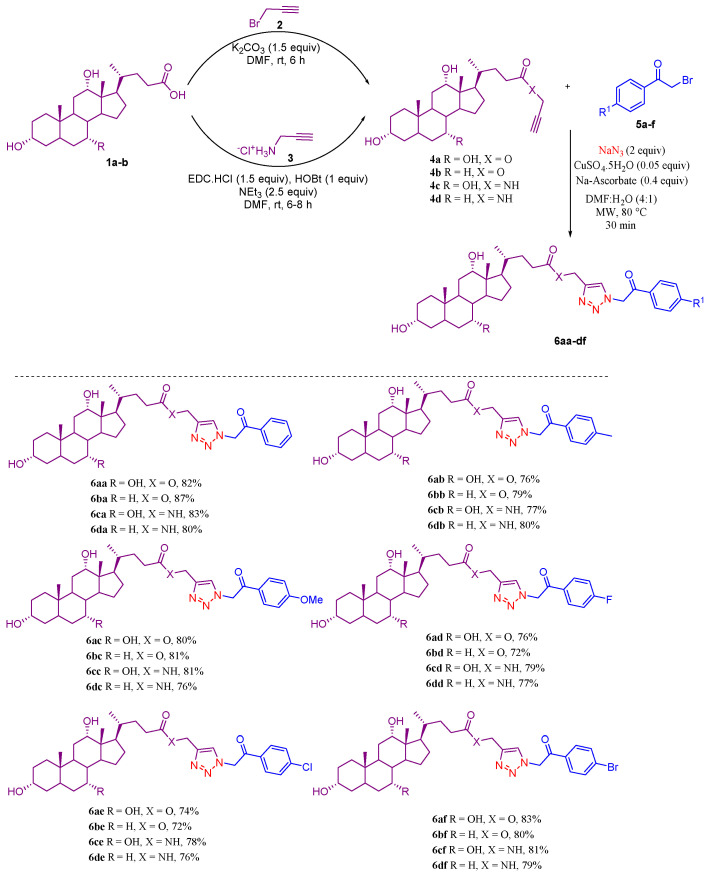
Synthesis of bile-acid-appended triazolyl aryl ketones.

**Figure 2:**
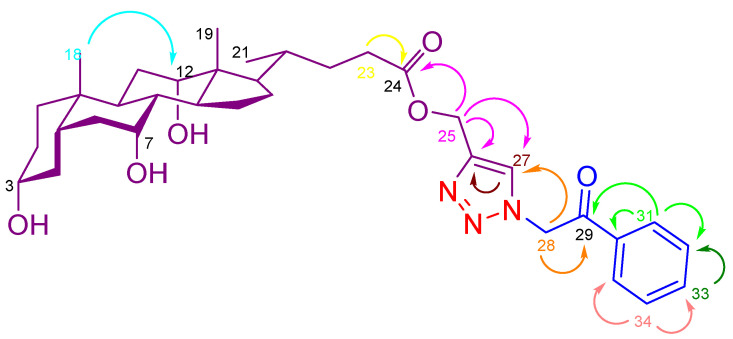
Selective correlations based on ^1^H,^13^C-HMBC spectrum of **6aa**.

From the outset of the proposed work, the synthesis of targeted bile-acid-appended triazolyl aryl ketones commenced with the preparation of cholic acid and deoxycholic acid propargyl esters (**4a**,**b**) and amides (**4c**,**d**) by coupling cholic acid (**1a**)/deoxycholic acid (**1b**) with propargyl bromide (**2**)/propargyl amine hydrochloride (**3**), respectively, using reported single-step protocols (Scheme 1) [41,44]. Thereafter, a Cu-catalyzed multicomponent reaction between CA and DCA propargyl esters/amides (**4a**–**d**) with α-bromoacetophenones (**5a**–**f**) and sodium azide in aqueous DMF under microwave irradiation at 80 °C comfortably afforded the desired bile-acid-appended triazolyl aryl ketones (**6aa**–**6df**) in excellent yields (Scheme 1). All the synthesized compounds were completely characterized on the basis of ^1^H NMR, ^13^C NMR and HRMS. As a representative example, the assignment of hydrogen and carbons in **6aa** was also performed using COSY, HSQC and HMBC (SI). The ^1^H and ^13^C NMR assignments for the representative proton/carbon signals of **6aa** are given in Table 1, and selective correlations are showcased in Figure 2 on the basis of the ^1^H, ^13^C HMBC spectrum, please see Supplementary Materials.

### 2.2. Biological Evaluation

#### 2.2.1. Cytotoxic Activity

All the synthesized compounds (**6aa**–**df**) were studied for their anticancer activity in two cancer lines viz. human breast adenocarcinoma (MCF-7) and mouse mammary carcinoma (4T1) cells at 10 µM concentration (Table 2). Most of the compounds showcased moderate-to-good activity against both cell lines as compared to the standard drug (docetaxel). Among all, compound **6af** was found to be the most active (26.52% cell viability at 10 µM) against MCF-7 cells. In addition, compounds **6bf** and **6cf** were also found to be active against human breast cancer cell line (MCF-7), exhibiting cell viabilities of 44.43% and 37.53%, respectively, at 10 µM. In 4T1 cells, **6cf** exhibited 49.27% cell viability at 10 µM. Triazolyl aryl ketones appended with cholic acid at the expense of an ester bond (**6aa**, **6ab**, **6ac**, **6ad**, **6af**) were found to be more active than their corresponding amide surrogates and deoxycholic acid ester/amide conjugates, except **6be** and **6ce**. In general, *para*-substitution (Me, OMe, F, Cl, Br) on the aryl ketone showcased lower cell viability as compared to the unsubstituted analogs. The analogs containing electron-withdrawing groups (F, Cl, Br) on aryl ketone were found to be more active as compared to the ones containing electron-donating groups (Me, OMe). Among halo-substituted analogs, triazolyl bromo-substituted aryl ketones appended to cholic acid and deoxycholic acid via an ester bond (**6af**, **6bf**) and amide bond (**6cf**) were found to be more active in inhibiting the growth of MCF-7 cells. While in 4T1 cells, triazolyl bromo-substituted aryl ketones appended to cholic acid and deoxycholic acid via an amide bond (**6cf**, **6df**) were found to be more active. In addition, adsorption, distribution, metabolism and excretion (ADME) properties and physiochemical properties of the synthesized analogs were calculated using molinspiration cheminformatics [45,46]. Additionally, percentage absorption and drug-likeness model score were also calculated using the reported formula [47] and Molsoft [48], respectively. As indicated by the TPSA values (between 60 and 160Å^2^), all the analogs (**6aa**–**df**) possessed better intestinal absorption ability over the blood–brain barrier (BBB) penetration power. Similarly, all the analogs (**6aa**–**df**) possessed a positive drug-likeness score between 0.60 and 1.14, indicating them to be ideal drug candidates. For instance, the most active analogs **6af**, **6bf** and **6cf** were found to possess relatively good drug-likeness scores of 0.94, 0.85 and 0.88, respectively.

Further, all the compounds (**6aa**–**df**) tested on a normal human embryonic kidney cell line (HEK 293) indicated cell viability to be greater than 85% and thus were found to be non-toxic against normal cells (Table 2). In particular, the most active derivatives **6af**, **6bf** and **6cf** possessing cell viability of 88.20, 86.82 and 87.82 appeared to be quite safer on normal human embryonic kidney cells.

IC_50_ values of the most potent compounds **6af**, **6bf** and **6cf** were further evaluated against the two cancer cell lines by employing MTT assay (Table 3). Interestingly, **6af**, **6bf** and **6cf** showed IC_50_ values in the range of 2.61–18.26 µM against the MCF-7 cancer cell line and 8.76–12.84 µM against the 4T1 cancer cell line. Compounds **6af** (IC_50_ = 2.6 µM) and **6cf** (IC_50_ = 5.71 µM) were found to possess pronounced anticancer activity as compared to the reference drug docetaxel (IC_50_ = 9.46 µM) against human breast adenocarcinoma (MCF-7), while all the three compounds (**6af**, **6bf**, **6cf**) were found to be more active with respect to docetaxel (IC_50_ = 13.85 µM) against rat mammary carcinoma (4T1). Further, these compounds did not induce cell death in HEK 293 cells.

#### 2.2.2. Apoptotic Study

**Figure 3:**
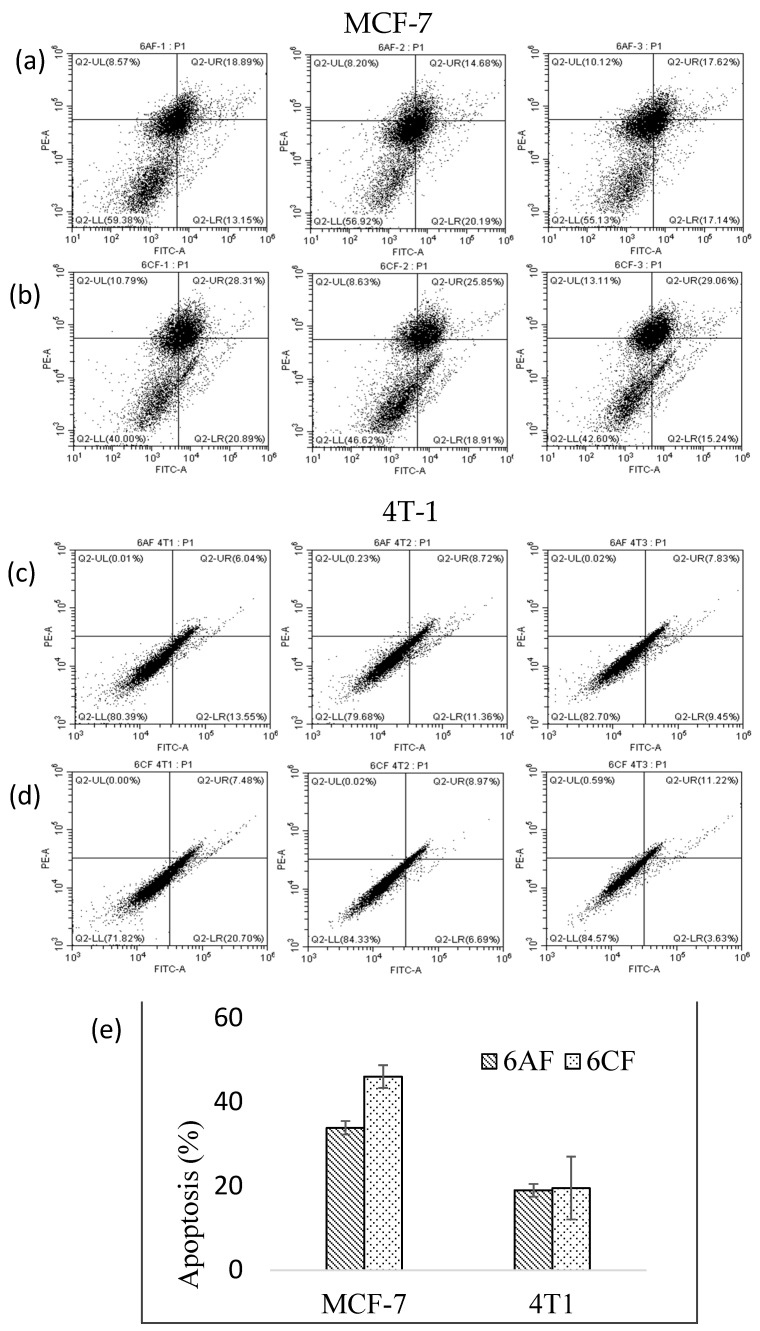
Apoptosis assay of **6af** and **6cf** in MCF-7 and 4T1 cells. (**a**,**b**) Flow cytometry plots for apoptosis in MCF-7 cells treated with **6af** and **6cf**, respectively. (**c**,**d**) Flow cytometry plots for apoptosis in 4T1 cells treated with **6af** and **6cf**, respectively. Upper left (necrotic cells), lower left (live cells), lower right (early apoptotic cells) and upper right (late apoptotic cells). (**e**) Graph showing the apoptosis (%) induced by **6af** and **6cf** in MCF-7 and 4T1 cells.

The apoptotic effect of **6af** and **6cf** was evaluated by the Annexin V/PI staining method. Following treatment of MCF-7 cells with **6af** and **6cf** at 2.61 µM and 5.71 µM, respectively, it was observed that compound **6cf** was capable of inducing higher apoptosis in comparison to **6af** (46.09% vs. 33.89%) (Figure 3a,b,e). Meanwhile, in 4T1 cells, both **6af** (at 12.84 µM) and **6cf** (at 8.76 µM) produced total apoptosis of 19.02% and 19.56%, indicating similar apoptotic potential in 4T1 cells (Figure 3c,d,e).

Of the two compounds, the most active compound **6cf** was chosen for the in vivo pharmacokinetic study.

#### 2.2.3. Pharmacokinetic Study of **6cf**

**Figure 4:**
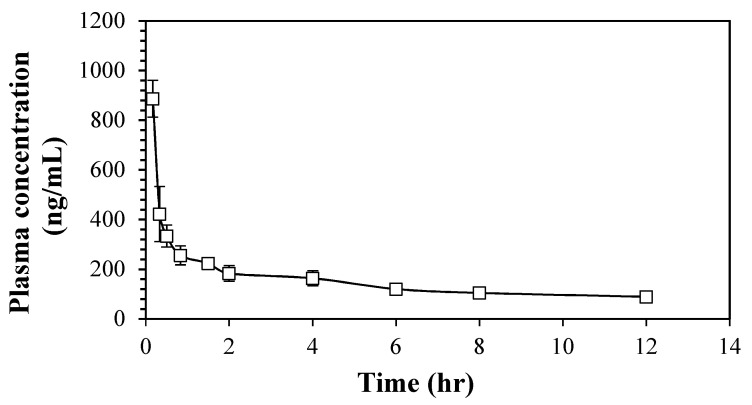
The pharmacokinetic profile for **6cf** in rat plasma after i.v. bolus at dose of 10 mg/kg administration to rat.

The relationship between the pharmacokinetic parameters and in vitro cytotoxicity study could be useful in determining the starting dose for the initial clinical trials for anticancer drugs. The compound **6cf** was found to have an IC_50_ (µM) of 5.71 and 8.76 µM in MCF-7 and 4T1 cells, respectively. The pharmacokinetic study was performed at a dose of 10 mg/kg i.v. bolus in rats that showed the initial concentration of 1752.69 ng/mL (~2.56 µM) with a half-life of 5.63 h. The mean plasma concentration–time profile of **6cf** after a single dose of 10 mg/kg (intravenously) in rats is presented in Figure 4. Different pharmacokinetic parameters were evaluated by a non-compartmental model approach using Phoenix WinNonlin software as shown in Table 4. The initial concentration (C_0_) was found to be 1752.69 ± 66.52 ng/mL. The AUC_0–last_ calculated based on the trapezoidal rule was found to be 1995.306 ± 87.43 ng.h/mL. The mean residence time (MRT) was found to be 8.47 ± 0.96 h. The **6cf** half-life was found to be 5.63 ± 0.54 h [49]. Although it may not be feasible to predict the in vivo concentrations at the tumor site from the plasma concentrations, the pharmacokinetic data provide initial insights into the mean residence time of the drug candidate and may be useful in predicting the dose relationship with the pharmacological/toxic effect after in vivo assessment in the tumor models. Thus, further assessment in tumor models to advance this molecule is warranted.

## 3. Materials and Methods

All the chemicals were purchased from Sigma-Aldrich (St. Louis, MO, USA), Alfa Aesar (Haverhill, MA, USA) and Spectrochem India Pvt. Ltd. (Mumbai, India) and used without further purification. The solvents were purchased from Merck (Burlington, MA, USA) and were distilled and dried before use. Nuclear magnetic resonance spectra were recorded on Bruker (Zurich, Switzerland) 400 spectrometer. The ^1^H NMR experiments were reported in δ units, parts per million (ppm), and were measured relative to residual chloroform (7.26 ppm) or DMSO-*d_6_* (2.5 ppm) in the deuterated solvent. The ^13^C NMR spectra were reported in ppm relative to deuterochloroform (77.0 ppm) or DMSO-*d_6_* (39.5 ppm). All coupling constants *J* were reported in Hz. The following abbreviations were used to describe peak splitting patterns when appropriate: s = singlet, d = doublet, t = triplet, dd = doublet of doublet, m = multiplet and brs = broad singlet. Melting points were determined on a capillary point apparatus equipped with a digital thermometer and were uncorrected. Reactions were monitored by using thin-layer chromatography (TLC) on 0.2 mm silica gel F254 plates (Merck). The chemical structures of final products were confirmed by a high-resolution ESI/APCI hybrid quadrupole time-of-flight mass spectrometer. High-resolution mass spectrometry (HRMS) was performed with a Waters SYNAPT G2 HDMS instrument using time-of-flight (TOF-MS) with ESI/APCI- hybrid quadrupole. The purity of final products was confirmed by high-performance liquid chromatography (HPLC), using the following chromatographic conditions: liquid chromatographic conditions, a Thermo Fisher Rapid Separation (RS) UHPLC System (Ultimate 3000, Waltham, MA, USA) equipped with a pump (LPG-3400SD), Diode Array Detector (DAD) (DAD-3000, Thermo Fisher, Waltham, MA, USA) and autosampler (ACC-3000T, Thermo Fisher, Waltham, MA, USA) were used for purity analysis. The UHPLC system was equilibrated for approximately 40 min before beginning the sample analysis. Control of hardware and data handling was performed using Chromeleon software version 7.2 SR4 (Thermo Fisher, Waltham, MA, USA). Column: Inertsil^®^ (GL Sciences, Tokyo, Japan) ODS C18 column (250 × 4.6 mm, 5µm). Mobile phase: ACN: water (95:05 % *v*/*v*); flow rate: 1 mL/min; detection wavelength: 259 nm; retention time: 3–5 min.

### 3.1. General Procedure for the Synthesis of CA and DCA Propargyl Amides *(**4c**,**d**)*

To a stirred solution of bile acid (CA (**1a**) or DCA (**1b**), 2.0 g, 1 equiv) in DMF (10 mL), triethyl amine (2.5 equiv) was added at 0 °C, and subsequently EDC.HCl (1.5 equiv) and HOBt (1 equiv) were added. The reaction mixture was stirred for 15 min at 0 °C, after which propargyl amine hydrochloride (1.5 equiv) was added. The reaction was stirred at room temperature for 6–8 h and was monitored by TLC. After the completion of the reaction, the reaction mixture was poured over crushed ice, and the resulted precipitate was filtered, washed with cold water, recrystallized from ethyl acetate/hexanes and triturated with diethyl ether to afford bile acid propargyl amide (**4c**,**d**), please see Supplementary Materials.

*(4R)-N-(Prop-2-yn-1-yl)-4-((3R,7R,10S,12S,13R)-3,7,12-trihydroxy-10,13-dimethylhexadecahydro-1H-cyclopenta[a]phenanthren-17-yl)pentanamide* (**4c**): White solid; Yield: 90% (1.96 g); mp: 277–279 °C (Lit. [41] 276–278 °C); ^1^H NMR (400 MHz, DMSO-*d_6_*) δ 8.23 (t, *J* = 5.5 Hz, 1H, NH_Amide_), 4.36 (d, *J* = 4.3 Hz, 1H, OH_CA_), 4.13 (d, *J* = 3.5 Hz, 1H, OH_CA_), 4.04 (d, *J* = 3.4 Hz, 1H, OH_CA_), 3.82 (dd, *J* = 5.6, 2.5 Hz, 2H), 3.78 (d, *J* = 3.4 Hz, 1H, H-12_CA_), 3.64–3.57 (m, 1H, H-7_CA_), 3.24–3.14 (m, 1H, H-3_CA_), 3.08 (t, *J* = 2.5 Hz, 1H, CH_Alkyne_), 2.25–2.12 (m, 2H), 2.04–1.92 (m, 2H), 1.84–1.59 (m, 6H), 1.48–1.06 (m, 14H), 0.92 (d, *J* = 6.4 Hz, 3H, Me-21_CA_), 0.81 (s, 3H, Me-19_CA_), 0.58 (s, 3H, Me-18_CA_); ^13^C NMR (100 MHz, DMSO-*d*_6_) δ 172.9 (C=O), 81.9, 73.2, 71.5 (C-12_CA_), 70.9 (C-3_CA_), 66.7 (C-7_CA_), 46.6, 46.2, 42.0, 41.8, 35.8, 35.6, 35.3, 34.9, 32.7, 32.0, 30.9, 29.0, 28.2, 27.8, 26.7, 23.3, 23.1 (C-19_CA_), 17.6 (C-21_CA_), 12.8 (C-18_CA_).

*(4R)-4-((3R,10S,12S,13R)-3,12-Dihydroxy-10,13-dimethylhexadecahydro-1H-cyclopenta[a]phenanthren-17-yl)-N-(prop-2-yn-1-yl)pentanamide* (**4d**): White solid; Yield: 89% (1.94 g); mp: 182–184 °C (Lit. [41] 184–186 °C); ^1^H NMR (400 MHz, DMSO-*d_6_*) δ 8.23 (t, *J* = 5.5 Hz, 1H, NH_Amide_), 4.50 (brs, 1H, OH_DCA_), 4.22 (brs, 1H, OH_DCA_), 3.82 (dd, *J* = 5.6, 2.5 Hz, 2H), 3.79 (d, *J* = 2.7 Hz, 1H, H-12_DCA_), 3.47 (brs, 1H, H-3_DCA_), 3.09 (t, *J* = 2.5 Hz, 1H, CH_Alkyne_), 2.13–1.95 (m, 2H), 1.83–1.74 (m, 2H), 1.72–1.42 (m, 8H), 1.39–1.06 (m, 14H), 0.91 (d, *J* = 6.4 Hz, 3H, Me-21_DCA_), 0.85 (s, 3H, Me-19_DCA_), 0.59 (s, 3H, Me-18_DCA_); ^13^C NMR (100 MHz, DMSO-*d*_6_) δ 172.8 (C=O), 81.9, 73.2, 71.5 (C-12_DCA_), 70.4 (C-3_DCA_), 47.9, 46.7, 46.4, 42.1, 36.7, 36.1, 35.6, 35.5, 34.3, 33.4, 32.6, 32.0, 30.7, 29.1, 28.2, 27.7, 27.4, 26.6, 24.0, 23.6 (C-19_DCA_), 17.5 (C-21_DCA_), 12.9 (C-18_DCA_).

### 3.2. General Procedure for the Synthesis of Bile-Acid-Appended Triazolyl Aryl Ketones *(**6aa**–**df**)*

Bile acid propargyl ester/propargyl amide (**4a**–**d**) (100 mg, 1 equiv), NaN_3_ (2 equiv), CuSO_4_.5H_2_O (0.05 equiv), sodium ascorbate (0.4 equiv) and substituted α-bromo acetone/phenacyl bromide (**5a**–**f**) (2 equiv) were added in a microwave vial containing DMF:H_2_O (4 mL:1 mL) mixture. The reaction mixture was stirred under microwave irradiation for 30 min at 80 °C, and the progress of the reaction was monitored by TLC (MeOH:DCM, 1% *v*/*v*). After the completion of the reaction, the mixture was quenched by adding crushed ice. The aqueous layer was extracted using ethyl acetate (2 × 20 mL). The combined organic layer was dried over anhydrous sodium sulfate, concentrated under reduced pressure and subjected to flash column chromatography (SiO_2_ (100–200 mesh), DCM:MeOH, 99:1 *v*/*v*) to yield pure bile-acid-appended triazolyl aryl ketone (**6aa**–**6df**), please see Supplementary Materials.

*(1-(2-Oxo-2-phenylethyl)-1H-1,2,3-triazol-4-yl)methyl (4R)-4-((3R,7R,10S,12S,13R)-3,7,12-trihydroxy-10,13-dimethylhexadecahydro-1H-cyclopenta[a]phenanthren-17-yl)pentanoate* (**6aa**): Off-white solid; Yield: 82% (0.111 g); mp: 108–109 °C; ^1^H NMR (400 MHz, DMSO-*d_6_*) δ 8.11 (s, 1H, H_Triazole_), 8.10–8.03 (m, 2H, H_Ar_), 7.80–7.70 (m, 1H, H_Ar_), 7.66–7.58 (m, 2H, H_Ar_), 6.20 (s, 2H), 5.18 (s, 2H), 4.33 (d, *J* = 4.3 Hz, 1H, OH_CA_), 4.12 (d, *J* = 3.8 Hz, 1H, OH_CA_), 4.01 (d, *J* = 3.3 Hz, 1H, OH_CA_), 3.78 (d, *J* = 3.8 Hz, 1H, H-12_CA_), 3.61 (brs, 1H, H-7_CA_), 3.17 (d, *J* = 5.1 Hz, 1H, H-3_CA_), 2.43–2.27 (m, 2H), 2.23–2.13 (m, 2H), 1.97–1.63 (m, 7H), 1.47–1.12 (m, 13H), 0.92 (d, *J* = 6.2 Hz, 3H, Me-21_CA_), 0.80 (s, 3H, Me-19_CA_), 0.57 (s, 3H, Me-18_CA_); ^13^C NMR (100 MHz, DMSO-*d*_6_) δ 192.5 (C=O), 173.6 (C=O), 142.4, 134.7, 134.6, 129.5, 128.6, 126.9, 71.5 (C-12_CA_), 70.9 (C-3_CA_), 66.7 (C-7_CA_), 57.5, 56.3, 46.6, 46.2, 42.0, 41.8, 35.8, 35.5, 35.3, 34.9, 31.1, 30.9, 29.0, 27.7, 26.7, 23.1 (C-19_CA_), 17.4 (C-21_CA_), 12.8 (C-18_CA_); HRMS (ESI): *m*/*z* [M + H]^+^ calcd for chemical formula C_35_H_50_N_3_O_6_^+^ 608.3694: found: 608.3685; Anal. RP-HPLC t_R_ = 3.773 min, purity 95.46%; ${\left[\alpha \right]}_{D}^{20}$ = +20 (*c* 1.0, MeOH).

*(1-(2-Oxo-2-phenylethyl)-1H-1,2,3-triazol-4-yl)methyl (4R)-4-((3R,10S,12S,13R)-3,12-dihydroxy-10,13-dimethylhexadecahydro-1H-cyclopenta[a]phenanthren-17-yl)pentanoate* (**6ba**): Off-white solid; Yield: 87% (0.119 g); mp: 143–144 °C; ^1^H NMR (400 MHz, DMSO-*d_6_*) δ 8.11 (s, 1H, H_Triazole_), 8.07 (d, *J* = 7.9 Hz, 2H, H_Ar_), 7.75 (t, *J* = 7.4 Hz, 1H, H_Ar_), 7.65–7.57 (d, *J* = 7.5 Hz, 2H, H_Ar_), 6.20 (s, 2H), 5.17 (s, 2H), 4.53 (d, *J* = 4.2 Hz, 1H, OH_DCA_), 4.24 (d, *J* = 3.9 Hz, 1H, OH_DCA_), 3.78 (brs, 1H, H-12_DCA_), 3.49 (brs, 1H, H-3_DCA_), 2.46–2.30 (m, 2H), 2.25–2.13 (m, 2H), 1.74–1.44 (m, 9H), 1.37–1.14 (m, 13H), 0.90 (d, *J* = 6.3 Hz, 3H, Me-21_DCA_), 0.84 (s, 3H, Me-19_DCA_), 0.57 (s, 3H, Me-18_DCA_)*;* ^13^C NMR (100 MHz, DMSO-*d_6_*) δ 192.6 (C=O), 173.6 (C=O), 142.3, 134.7, 134.5, 129.5, 128.6, 126.9, 71.5 (C-12_DCA_), 70.4 (C-3_DCA_), 57.5, 56.3, 47.9, 46.6, 46.5, 42.1, 36.7, 36.1, 35.6, 35.4, 34.3, 33.4, 31.1, 30.7, 29.0, 27.6, 27.4, 26.6, 23.6 (C-19_DCA_), 17.3 (C-21_DCA_), 12.9 (C-18_DCA_); HRMS (ESI): *m*/*z* [M + H]^+^ calcd for chemical formula C_35_H_50_N_3_O_5_^+^ 592.3745 found: 592.3733; Anal. RP-HPLC t_R_ = 4.553 min, purity 94.55%; ${\left[\alpha \right]}_{D}^{20}$ = +16 (*c* 1.0, MeOH).

*(4R)-N-((1-(2-Oxo-2-phenylethyl)-1H-1,2,3-triazol-4-yl)methyl)-4-((3R,7R,10S,12S,13R)-3,7,12-trihydroxy-10,13-dimethylhexadecahydro-1H-cyclopenta[a]phenanthren-17-yl)pentanamide* (**6ca**): Off-white solid; Yield: 83% (0.112 g); mp: 148–149 °C; ^1^H NMR (400 MHz, DMSO-*d_6_*) δ 8.39 (t, *J* = 5.8 Hz, 1H, NH_Amide_), 8.09–8.05 (m, 2H, H_Ar_), 7.84 (s, 1H, H_Triazole_), 7.77–7.72 (m, 1H, H_Ar_), 7.61 (t, *J* = 7.7 Hz, 2H, H_Ar_), 6.15 (s, 2H), 4.39 (brs, 1H, OH_CA_), 4.33 (d, *J* = 5.7 Hz, 2H), 4.12 (d, *J* = 3.7 Hz, 1H, OH_CA_), 4.03 (d, *J* = 3.5 Hz, 1H, OH_CA_), 3.78 (d, *J* = 3.8 Hz, 1H, H-12_CA_), 3.60 (brs, 1H, H-7_CA_), 3.18 (s, 1H, H-3_CA_), 2.19–2.10 (m, 2H), 2.05 – 1.94 (m, 2H), 1.80–1.60 (m, 6H), 1.49–1.15 (m, 14H), 0.93 (d, *J* = 6.1 Hz, 3H, Me-21_CA_), 0.79 (s, 3H, Me-19_CA_), 0.56 (s, 3H, Me-18_CA_); ^13^C NMR (100 MHz, DMSO-*d_6_*) δ 192.7 (C=O), 173.2 (C=O), 145.6, 134.7, 134.6, 129.5, 128.6, 124.9, 71.5 (C-12_CA_), 70.9 (C-3_CA_), 66.7 (C-7_CA_), 56.2, 54.7, 46.6, 46.2, 41.9, 41.8, 35.6, 35.3, 34.8, 34.6, 32.1, 30.8, 29.0, 27.8, 26.7, 23.1 (C-19_CA_), 17.6 (C-21_CA_), 12.8 (C-18_CA_); HRMS (ESI): *m*/*z* [M + H]^+^ calcd for chemical formula: C_35_H_51_N_4_O_5_^+^ 607.3854 found: 607.3840; Anal. RP-HPLC t_R_ = 3.780 min, purity 95.86%; ${\left[\alpha \right]}_{D}^{20}$ = +58 (*c* 1.0, MeOH).

*(4R)-4-((3R,10S,12S,13R)-3,12-Dihydroxy-10,13-dimethylhexadecahydro-1H-cyclopenta[a]phenanthren-17-yl)-N-((1-(2-oxo-2-phenylethyl)-1H-1,2,3-triazol-4-yl)methyl)pentanamide* (**6da**): Off-white solid; Yield: 80% (0.110 g); mp: 135–136 °C; ^1^H NMR (400 MHz, DMSO-*d_6_*) δ 8.39 (t, *J* = 5.7 Hz, 1H, NH_Amide_), 8.06 (d, *J* = 7.1 Hz, 2H, H_Ar_), 7.84 (s, 1H, H_Triazole_), 7.74 (t, *J* = 7.4 Hz, 1H, H_Ar_), 7.61 (t, *J* = 7.7 Hz, 2H, H_Ar_), 6.15 (s, 2H), 4.53 (d, *J* = 4.2 Hz, 1H, OH_DCA_), 4.32 (d, *J* = 5.7 Hz, 2H), 4.22 (d, *J* = 4.0 Hz, 1H, OH_DCA_), 3.78 (brs, 1H, H-12_DCA_), 3.48 (brs, 1H, H-3_DCA_), 2.17–1.98 (m, 2H), 1.76–1.44 (m, 10H), 1.37–1.14 (m, 14H), 0.91 (d, *J* = 6.3 Hz, 3H, Me-21_DCA_), 0.82 (s, 3H, Me-19_DCA_), 0.56 (s, 3H, Me-18_DCA_); ^13^C NMR (100 MHz, DMSO-*d_6_*) δ 192.7 (C=O), 173.1 (C=O), 145.6, 134.7, 134.6, 129.4, 128.6, 124.8, 71.5 (C-12_DCA_), 70.4 (C-3_DCA_), 56.2, 47.9, 46.7, 46.4, 42.1, 36.7, 36.1, 35.6, 35.5, 34.6, 34.3, 33.4, 32.8, 32.1, 30.7, 29.1, 27.7, 27.4, 26.6, 24.0, 23.6 (C-19_DCA_), 17.5 (C-21_DCA_), 12.9 (C-18_DCA_); HRMS (ESI): *m*/*z* [M + H]^+^ calcd for chemical formula: C_35_H_51_N_4_O_4_^+^ 591.3905 found: 591.3883; Anal. RP-HPLC t_R_ = 3.610 min, purity 96.50%; ${\left[\alpha \right]}_{D}^{20}$ = +9 (*c* 1.0, MeOH).

*(1-(2-Oxo-2-(p-tolyl)ethyl)-1H-1,2,3-triazol-4-yl)methyl (4R)-4-((3R,7R,10S,12S,13R)-3,7,12-trihydroxy-10,13-dimethylhexadecahydro-1H-cyclopenta[a]phenanthren-17-yl)pentanoate* (**6ab**): White solid; Yield: 76% (0.103 g); mp: 121–124 °C; ^1^H NMR (400 MHz, DMSO-*d_6_*) δ 8.10 (s, 1H, H_Triazole_), 7.97 (d, *J* = 8.3 Hz, 2H, H_Ar_), 7.42 (d, *J* = 8.0 Hz, 2H, H_Ar_), 6.15 (s, 2H), 5.16 (s, 2H), 4.38 (d, *J* = 4.4 Hz, 1H, OH_CA_), 4.15 (d, *J* = 3.5 Hz, 1H, OH_CA_), 4.05 (d, *J* = 3.3 Hz, 1H, OH_CA_), 3.77 (d, *J* = 3.4 Hz, 1H, H-12_CA_), 3.60 (brs, 1H, H-7_CA_), 3.21–3.14 (m, 1H, H-3_CA_), 2.42 (s, 3H, Me_Ar_), 2.30–2.18 (m, 2H), 2.14–1.92 (m, 2H), 1.84–1.55 (m, 8H), 1.45–1.66 (m, 12H), 0.91 (d, *J* = 6.1 Hz, 3H, Me-21_CA_), 0.79 (s, 3H, Me-19_CA_), 0.56 (s, 3H, Me-18_CA_); ^13^C NMR (100 MHz, DMSO-*d*_6_) δ 192.0 (C=O), 173.6 (C=O), 145.3, 142.3, 132.0, 130.0, 128.7, 126.9, 71.4 (C-12_CA_), 70.9 (C-3_CA_), 66.7 (C-7_CA_), 57.5, 56.2, 46.5, 46.2, 42.0, 41.8, 35.4, 35.3, 34.8, 31.1, 31.0, 30.8, 29.0, 27.7, 26.6, 23.1 (C-19_CA_), 21.8 (Me_Ar_), 17.3 (C-21_CA_), 12.8 (C-18_CA_); Anal. RP-HPLC t_R_ = 4.010 min, purity 99.37%; HRMS (ESI): *m*/*z* [M + H]^+^ calcd for chemical formula: C_36_H_52_N_3_O_6_^+^ 622.3851 found: 622.3837; ${\left[\alpha \right]}_{D}^{20}$ = +29 (*c* 1.0, MeOH).

*(1-(2-Oxo-2-(p-tolyl)ethyl)-1H-1,2,3-triazol-4-yl)methyl (4R)-4-((3R,10S,12S,13R)-3,12-dihydroxy-10,13-dimethylhexadecahydro-1H-cyclopenta[a]phenanthren-17-yl)pentanoate* (**6bb**): White solid; Yield: 79% (0.110 g); mp: 123–125 °C; ^1^H NMR (400 MHz, DMSO-*d_6_*) δ 8.10 (s, 1H, H_Triazole_), 7.97 (d, *J* = 8.2 Hz, 2H, H_Ar_), 7.42 (d, *J* = 8.0 Hz, 2H, H_Ar_), 6.15 (s, 2H), 5.16 (s, 2H), 4.53 (d, *J* = 4.3 Hz, 1H, OH_DCA_), 4.24 (d, *J* = 4.1 Hz, 1H, OH_DCA_), 3.78 (d, *J* = 3.8 Hz, 1H, H-12_DCA_), 3.47 (brs, 1H, H-3_DCA_), 2.42 (s, 3H, Me_Ar_), 2.39–2.24 (m, 2H), 1.85–1.50 (m, 10H), 1.47–1.15 (m, 14H), 0.90 (d, *J* = 6.2 Hz, 3H, Me-21_DCA_), 0.83 (s, 3H, Me-19_DCA_), 0.57 (s, 3H, Me-18_DCA_);^13^C NMR (100 MHz, DMSO-*d*_6_) δ 192.0 (C=O), 173.6 (C=O), 145.3, 142.3, 132.0, 130.0, 128.7, 126.9, 71.5 (C-12_DCA_), 70.4 (C-3_DCA_), 57.5, 56.2, 47.9, 46.6, 46.5, 42.0, 36.7, 36.1, 35.6, 35.4, 34.3, 33.4, 31.1, 30.7, 29.0, 27.6, 27.4, 26.6, 24.0, 23.5 (C-19_DCA_), 21.8, 17.3 (C-21_DCA_), 12.9 (C-18_DCA_); HRMS (ESI): *m*/*z* [M]^+^ calcd for chemical formula: C_36_H_52_N_3_O_5_^+^ 606.3901 found: 606.3874; Anal. RP-HPLC t_R_ = 4.813 min, purity 95.99% ${\left[\alpha \right]}_{D}^{20}$ = +47 (*c* 1.0, MeOH).

*(4R)-N-((1-(2-Oxo-2-(p-tolyl)ethyl)-1H-1,2,3-triazol-4-yl)methyl)-4-((3R,7R,10S,12S,13R)-3,7,12-trihydroxy-10,13-dimethylhexadecahydro-1H-cyclopenta[a]phenanthren-17-yl)pentanamide* (**6cb**): White solid; Yield: 77% (0.107 g); mp: 129–131 °C; ^1^H NMR (400 MHz, DMSO-*d_6_*) δ 8.39 (t, *J* = 5.7 Hz, 1H, NH_Amide_), 7.96 (d, *J* = 8.2 Hz, 2H, H_Ar_), 7.82 (s, 1H, H_Triazole_), 7.41 (d, *J* = 8.1 Hz, 2H, H_Ar_), 6.11 (s, 2H), 4.38 (d, *J* = 4.3 Hz, 1H, OH_CA_), 4.32 (d, *J* = 5.7 Hz, 2H), 4.13 (d, *J* = 3.5 Hz, 1H, OH_CA_), 4.04 (d, *J* = 3.3 Hz, 1H, OH_CA_), 3.77 (d, *J* = 3.6 Hz, 1H, H-12_CA_), 3.59 (brs, 1H, H-7_CA_), 3.23–3.16 (m, 1H, H-3_CA_), 2.41 (s, 3H, Me_Ar_), 2.22–2.11 (m, 2H), 2.06–1.94 (m, 2H), 1.82–1.58 (m, 7H), 1.50–1.11 (m, 13H), 0.92 (d, *J* = 6.2 Hz, 3H, Me-21_CA_), 0.78 (s, 3H, Me-19_CA_), 0.55 (s, 3H, Me-18_CA_);^13^C NMR (100 MHz, DMSO-*d*_6_) δ 192.1 (C=O), 173.2 (C=O), 145.6, 145.3, 132.1, 130.0, 128.7, 124.8, 71.5 (C-12_CA_), 70.9 (C-3_CA_), 66.7 (C-7_CA_), 56.1, 46.6, 46.2, 42.0, 41.8, 35.8, 35.6, 35.3, 34.8, 34.6, 32.8, 32.1, 30.8, 29.0, 27.8, 26.7, 23.1 (C-19_CA_), 21.7 (Me_Ar_), 17.6 (C-21_CA_), 12.8 (C-18_CA_);HRMS (ESI): *m*/*z* [M + H]^+^ calcd for chemical formula: C_36_H_53_N_4_O_5_^+^ 621.4010 found: 621.4006; Anal. RP-HPLC t_R_ = 3.770 min, purity 95.06%; ${\left[\alpha \right]}_{D}^{20}$ = +19 (*c* 1.0, MeOH).

*(4R)-4-((3R,10S,12S,13R)-3,12-Dihydroxy-10,13-dimethylhexadecahydro-1H-cyclopenta[a]phenanthren-17-yl)-N-((1-(2-oxo-2-(p-tolyl)ethyl)-1H-1,2,3-triazol-4-yl)methyl)pentanamide* (**6db**): White solid Yield: 80% (0.112 g); mp: 112–115 °C; ^1^H NMR (400 MHz, DMSO-*d_6_*) δ 8.37 (t, *J* = 5.8 Hz, 1H, NH_Amide_), 7.97 (d, *J* = 8.2 Hz, 2H, H_Ar_), 7.82 (s, 1H, H_Triazole_), 7.41 (d, *J* = 8.1 Hz, 2H, H_Ar_), 6.11 (s, 2H), 4.48 (d, *J* = 4.2 Hz, 1H, OH_DCA_), 4.32 (d, *J* = 5.7 Hz, 2H), 4.20 (d, *J* = 4.1 Hz, 1H, OH_DCA_), 3.77 (d, *J* = 3.8 Hz, 1H, H-12_DCA_), 3.37 (brs, 1H, H-3_DCA_), 2.42 (s, 3H, Me_Ar_), 2.15–1.95 (m, 2H), 1.82–1.42 (m, 11H), 1.37–1.13 (m, 13H), 0.92 (d, *J* = 6.3 Hz, 3H, Me-21_DCA_), 0.82 (s, 3H, Me-19_DCA_), 0.56 (s, 3H, Me-18_DCA_); ^13^C NMR (100 MHz, DMSO-*d*_6_) δ 192.1 (C=O), 173.0 (C=O), 145.6, 145.2, 132.1, 130.0, 128.7, 124.8, 71.5 (C-12_DCA_), 70.4 (C-3_DCA_), 56.1, 47.9, 46.7, 46.4, 42.1, 36.1, 35.5, 34.6, 34.3, 33.4, 32.8, 32.1, 29.1, 27.7, 27.4, 26.6, 24.0, 23.5 (C-19_DCA_), 21.8 (Me_Ar_), 17.5 (C-21_DCA_), 12.9 (C-18_DCA_); HRMS (ESI): *m*/*z* [M + H]^+^ calcd for chemical formula: C_36_H_53_N_4_O_4_^+^ 605.4035 found: 605.4061; Anal. RP-HPLC t_R_ = 4.100 min, purity 93.82%; ${\left[\alpha \right]}_{D}^{20}$ = +(*c* 1.0, MeOH).

*(1-(2-(4-Methoxyphenyl)-2-oxoethyl)-1H-1,2,3-triazol-4-yl)methyl (4R)-4-((3R,7R,10S,12S,13R)-3,7,12-trihydroxy-10,13-dimethylhexadecahydro-1H-cyclopenta[a]phenanthren-17-yl)pentanoate* (**6ac**): Off-white solid; Yield: 80% (0.114 g); mp: 176–177 °C; ^1^H NMR (400 MHz, DMSO-*d_6_*) δ 8.10 (s, 1H, H_Triazole_), 8.05 (d, *J* = 8.9 Hz, 2H, 2H, H_Ar_), 7.13 (d, *J* = 9.0 Hz, 2H, H_Ar_), 6.13 (s, 2H), 5.17 (s, 2H), 4.36 (brs, 1H, OH_CA_), 4.14 (d, *J* = 3.5 Hz, 1H, OH_CA_), 4.04 (d, *J* = 3.3 Hz, 1H, OH_CA_), 3.88 (s, 3H, OMe_Ar_), 3.77 (d, *J* = 3.1 Hz, 1H, H-12_CA_), 3.60 (brs, 1H, H-7_CA_), 3.22–3.15 (m, 1H, H-3_CA_), 2.40–2.24 (m, 2H), 2.23–2.10 (m, 2H), 1.80–1.61 (m, 6H), 1.49–1.09 (m, 14H), 0.91 (d, *J* = 6.1 Hz, 3H, Me-21_CA_), 0.80 (s, 3H, Me-19_CA_), 0.57 (s, 3H, Me-18_CA_); ^13^C NMR (100 MHz, DMSO-*d*_6_) δ 190.8 (C=O), 173.6 (C=O), 164.4, 142.3, 131.1, 127.4, 126.9, 114.7, 71.4 (C-12_CA_), 70.9 (C-3_CA_), 66.7 (C-7_CA_), 57.5, 56.2, 55.9, 46.5, 46.2, 42.0, 41.8, 35.8, 35.5, 35.3, 34.8, 31.1, 31.0, 30.9, 29.0, 27.7, 26.7, 23.3, 23.1 (C-19_CA_), 17.4 (C-21_CA_), 12.8 (C-18_CA_); HRMS (ESI): *m*/*z* [M + H]^+^ calcd for chemical formula: C_36_H_52_N_3_O_7_^+^ 638.3800 found: 638.3798; Anal. RP-HPLC t_R_ = 4.003 min, purity 99.43%; ${\left[\alpha \right]}_{D}^{20}$ = +31 (*c* 1.0, MeOH).

*(1-(2-(4-Methoxyphenyl)-2-oxoethyl)-1H-1,2,3-triazol-4-yl)methyl (4R)-4-((3R,10S,12S,13R)-3,12-dihydroxy-10,13-dimethylhexadecahydro-1H-cyclopenta[a]phenanthren-17-yl)pentanoate* (**6bc**): Off-white solid; Yield: 81% (0.116 g); mp: 144–45 °C; ^1^H NMR (400 MHz, DMSO-*d_6_*) δ 8.09 (s, 1H, H_Triazole_), 8.05 (d, *J* = 8.8 Hz, 2H, H_Ar_), 7.13 (d, *J* = 8.9 Hz, 2H, H_Ar_), 6.12 (s, 2H), 5.17 (s, 2H), 4.52 (d, *J* = 4.3 Hz, 1H, OH_DCA_), 4.22 (d, *J* = 4.1 Hz, 1H, OH_DCA_), 3.88 (s, 3H, OMe_Ar_), 3.78 (d, *J* = 4.2 Hz, 1H, H-12_DCA_), 3.46 (brs, 1H, H-3_DCA_), 2.43–2.31 (m, 2H), 2.29–2.15 (m, 2H), 1.78–1.55 (m, 10H), 1.35–1.20 (m, 12H), 0.91 (d, *J* = 6.2 Hz, 3H, Me-21_DCA_), 0.84 (s, 3H, Me-19 _DCA_), 0.57 (s, 3H, Me-18_DCA_); ^13^C NMR (100 MHz, DMSO-*d*_6_) δ 190.8 (C=O), 173.6 (C=O), 164.4, 142.3, 131.1, 127.4, 126.9, 114.7, 71.5 (C-12_DCA_), 70.5 (C-3_DCA_), 57.5, 56.2, 55.9, 47.9, 46.6, 46.5, 42.0, 36.7, 36.1, 35.6, 35.4, 34.3, 33.4, 31.0, 30.7, 29.0, 27.6, 27.4, 26.5, 23.5 (C-19_DCA_), 17.3 (C-21_DCA_), 12.9 (C-18_DCA_); HRMS (ESI): *m*/*z* [M + H]^+^ calcd for chemical formula: C_36_H_52_N_3_O_6_^+^ 622.3851 found: 622.3853; Anal. RP-HPLC t_R_ = 4.797 min, purity 97.61%; ${\left[\alpha \right]}_{D}^{20}$ = +11 (*c* 1.0, MeOH).

*(4R)-N-((1-(2-(4-Methoxyphenyl)-2-oxoethyl)-1H-1,2,3-triazol-4-yl)methyl)-4-((3R,7R,10S,12S,13R)-3,7,12-trihydroxy-10,13-dimethylhexadecahydro-1H-cyclopenta[a]phenanthren-17-yl)pentanamide* (**6cc**): Off-white solid; Yield: 81% (0.115 g); mp: 158–159 °C; ^1^H NMR (400 MHz, DMSO-*d_6_*) δ 8.38 (t, *J* = 5.8 Hz, 1H, NH_Amide_), 8.04 (d, *J* = 8.9 Hz, 2H, H_Ar_), 7.82 (s, 1H, H_Triazole_), 7.12 (d, *J* = 9.0 Hz, 2H, H_Ar_), 6.08 (s, 2H), 4.36 (brs, 1H, OH_CA_), 4.34–4.29 (m, 2H), 4.12 (d, *J* = 3.5 Hz, 1H, OH_CA_), 4.03 (d, *J* = 3.5 Hz, 1H, OH_CA_), 3.87 (s, 3H, OMe_Ar_), 3.77 (d, *J* = 3.5 Hz, 1H, H-12_CA_), 3.59 (brs, 1H, H-7_CA_), 3.22–3.16 (m, 1H, H-3_CA_), 2.20–2.13 (m, 2H), 2.04–1.95 (m, 2H), 1.81–1.63 (m, 6H), 1.48–1.29 (m, 8H), 1.27–1.17 (m, 6H), 0.92 (d, *J* = 6.2 Hz, 3H, Me-21_CA_), 0.78 (s, 3H, Me-19_CA_), 0.55 (s, 3H, Me-18_CA_);^13^C NMR (100 MHz, DMSO-*d*_6_) δ 190.9 (C=O), 173.1 (C=O), 164.3, 145.6, 131.0, 127.5, 124.8, 114.7, 71.5 (C-12_CA_), 70.9 (C-3_CA_), 66.7 (C-7_CA_), 56.2, 55.8, 46.6, 46.2, 42.0, 41.8, 35.8, 35.6, 35.3, 34.8, 34.6, 32.1, 30.9, 29.0, 27.8, 26.7, 23.3, 23.1 (C-19_CA_), 17.5 (C-21_CA_), 12.8 (C-18_CA_); HRMS (ESI): *m*/*z* [M + H]^+^ calcd for chemical formula: C_36_H_53_N_4_O_6_^+^ 637.3960 found: 637.3961; Anal. RP-HPLC t_R_ = 4.097 min, purity 93.92%; ${\left[\alpha \right]}_{D}^{20}$ = +47 (*c* 1.0, MeOH).

*(4R)-4-((3R,10S,12S,13R)-3,12-Dihydroxy-10,13-dimethylhexadecahydro-1H-cyclopenta[a]phenanthren-17-yl)-N-((1-(2-(4-methoxyphenyl)-2-oxoethyl)-1H-1,2,3-triazol-4-yl)methyl)pentanamide* (**6dc**): Off-white solid; Yield: 76% (0.109 g); mp: 114–115 °C; ^1^H NMR (400 MHz, DMSO-*d_6_*) δ 8.38 (t, *J* = 5.7 Hz, 1H, NH_Amide_), 8.05 (d, *J* = 8.9 Hz, 2H, H_Ar_), 7.82 (s, 1H, H_Triazole_), 7.12 (d, *J* = 9.0 Hz, 2H, H_Ar_), 6.08 (s, 2H), 4.51–4.49 (m, 1H, OH_DCA_), 4.32 (d, *J* = 5.7 Hz, 2H), 4.21 (d, *J* = 4.1 Hz, 1H, OH_DCA_), 3.88 (s, 3H, OMe_Ar_), 3.78 (d, *J* = 4.0 Hz, 1H, H-12_DCA_), 3.40 (brs, 1H, H-3_DCA_), 2.01–2.03 (m, 2H), 2.00–1.80 (d, *J* = 8.3 Hz, 2H), 1.77–1.47 (m, 10H), 1.–1.18 (m, 12H), 0.92 (d, *J* = 6.3 Hz, 3H, Me-21_DCA_), 0.82 (s, 3H, Me-19_DCA_), 0.56 (s, 3H, Me-18_DCA_); ^13^C NMR (100 MHz, DMSO-*d*_6_) δ 190.9 (C=O), 173.1 (C=O), 164.3, 145.6, 131.0, 127.5, 124.8, 114.7, 71.5 (C-12_DCA_), 70.4 (C-3_DCA_), 56.2, 55.8, 47.9, 46.7, 46.4, 42.1, 36.7, 36.1, 35.6, 35.5, 34.6, 34.3, 33.4, 32.8, 32.1, 30.7, 29.1, 27.7, 27.4, 26.6, 23.5 (C-19_DCA_), 17.5 (C-21_DCA_), 12.9 (C-18_DCA_); HRMS (ESI): *m*/*z* [M + H]^+^ calcd for chemical formula: C_36_H_53_N_4_O_5_^+^ 621.4010 found: 621.4009; Anal. RP-HPLC t_R_ = 4.097 min, purity 94.90%; ${\left[\alpha \right]}_{D}^{20}$ = +21 (*c* 1.0, MeOH).

*(1-(2-(4-Fluorophenyl)-2-oxoethyl)-1H-1,2,3-triazol-4-yl)methyl (4R)-4-((3R,7R,10S,12S,13R)-3,7,12-trihydroxy-10,13-dimethylhexadecahydro-1H-cyclopenta[a]phenanthren-17-yl)pentanoate* (**6ad**): Off-white solid; Yield: 76% (0.106 g); mp: 152–153 °C; ^1^H NMR (400 MHz, DMSO-*d_6_*) δ 8.19–8.09 (m, 3H, H_Triazole_&H_Ar_), 7.46 (t, *J* = 8.7 Hz, 2H, H_Ar_), 6.19 (s, 2H), 5.17 (s, 2H), 4.35 (d, *J* = 4.3 Hz, 1H, OH_CA_), 4.13 (d, *J* = 3.4 Hz, 1H, OH_CA_), 4.03 (d, *J* = 3.7 Hz, 1H, OH_CA_), 3.77 (brs, 1H, H-12_CA_), 3.61 (brs, 1H, H-7_CA_), 3.22–3.16 (m, 1H, H-3_CA_), 2.41–2.24 (m, 2H), 2.21–2.14 (m, 2H), 1.83–1.67 (m, 5H), 1.64–1.40 (m, 5H), 1.35–1.17 (m, 10H), 0.91 (d, *J* = 5.9 Hz, 3H, Me-21_CA_), 0.80 (s, 3H, Me-19_CA_), 0.57 (s, 3H, Me-18_CA_);^13^C NMR (100 MHz, DMSO-*d*_6_) δ 191.3 (C=O), 173.6 (C=O), 142.4, 131.8, 132.7, 126.9, 116.7, 116.5, 71.4 (C-12_CA_), 70.9 (C-3_CA_), 66.7 (C-7_CA_), 57.5, 56.2, 46.5, 46.2, 42.0, 35.8, 35.5, 35.3, 34.8, 31.1, 31.1, 30.9, 29.0, 27.7, 26.7, 23.3, 23.1 (C-19_CA_), 17.4 (C-21_CA_), 12.8 (C-18_CA_); HRMS (ESI): *m*/*z* [M + H]^+^ calcd for chemical formula: C_35_H_49_N_3_O_6_F^+^ 626.3600 found: 626.3600; Anal. RP-HPLC t_R_ = 3.783 min, purity 95.12%; ${\left[\alpha \right]}_{D}^{20}$ = +51 (*c* 1.0, MeOH).

*(1-(2-(4-Fluorophenyl)-2-oxoethyl)-1H-1,2,3-triazol-4-yl)methyl (4R)-4-((3R,10S,12S,13R)-3,12-dihydroxy-10,13-dimethylhexadecahydro-1H-cyclopenta[a]phenanthren-17-yl)pentanoate* (**6bd**): Off-white solid; Yield: 72% (0.101 g); mp: 127–128 °C; ^1^H NMR (400 MHz, DMSO-*d_6_*) δ 8.19–8.14 (m, 2H, H_Ar_), 8.10 (s, 1H, H_Triazole_), 7.46 (t, *J* = 8.9 Hz, 2H, H_Ar_), 6.19 (s, 2H), 5.17 (s, 2H), 4.49 (d, *J* = 4.3 Hz, 1H, OH_DCA_), 4.22 (d, *J* = 4.0 Hz, 1H, OH_DCA_), 3.78 (d, *J* = 4.1 Hz, 1H, H-12_DCA_), 3.43–3.40 (m, 1H, H-3_DCA_), 2.40–2.22 (m, 2H), 1.81–1.70 (m, 5H), 1.62–1.44 (m, 5H), 1.36–1.11 (m, 14H), 0.91 (d, *J* = 6.1 Hz, 3H, Me-21_DCA_), 0.84 (s, 3H, Me-19_DCA_), 0.57 (s, 3H, Me-18_DCA_); ^13^C NMR (100 MHz, DMSO-*d*_6_) δ 191.3 (C=O), 173.6 (C=O), 142.4, 131.8, 131.7, 126.9, 116.7, 116.5, 71.5 (C-12_DCA_), 70.4 (C-3_DCA_), 57.5, 56.2, 47.9, 46.6, 46.5, 42.1, 36.7, 36.1, 35.6, 35.4, 34.3, 33.4, 31.1, 30.7, 29.0, 27.6, 27.4, 26.6, 24.0, 23.6 (C-19_DCA_), 17.3 (C-21_DCA_), 12.9 (C-18_DCA_); HRMS (ESI): *m*/*z* [M + H]^+^ calcd for chemical formula: C_35_H_49_N_3_O_5_F^+^ 610.3651 found: 610.3650; Anal. RP-HPLC t_R_ = 5.333 min, purity 96.72%; ${\left[\alpha \right]}_{D}^{20}$ = +8 (*c* 1.0, MeOH).

*(4R)-N-((1-(2-(4-Fluorophenyl)-2-oxoethyl)-1H-1,2,3-triazol-4-yl)methyl)-4-((3R,7R,10S,12S,13R)-3,7,12-trihydroxy-10,13-dimethylhexadecahydro-1H-cyclopenta[a]phenanthren-17-yl)pentanamide* (**6cd**): Off-white solid; Yield: 79% (0.110 g); mp: 137–138 °C; ^1^H NMR (400 MHz, DMSO-*d_6_*) δ 8.40 (t, *J* = 5.7 Hz, 1H, NH_Amide_), 8.15 (dd, *J* = 8.8, 5.5 Hz, 2H, H_Ar_), 7.83 (s, 1H, H_Triazole_), 7.45 (t, *J* = 8.9 Hz, 2H, H_Ar_), 6.15 (s, 2H), 4.44 (brs, 1H, OH_CA_), 4.32 (d, *J* = 5.7 Hz, 2H), 4.14 (brs, 1H, OH_CA_), 4.06–4.01 (m, 1H, OH_CA_), 3.77 (d, *J* = 3.0 Hz, 1H, H-12_CA_), 3.61–3.58 (m, 1H, H-7_CA_), 3.21–3.16 (m, 1H, H-3_CA_), 2.16–2.12 (m, 2H), 2.03–1.99 (m, 2H), 1.80–1.60 (m, 7H), 1.44–1.12 (m, 13H), 0.92 (d, *J* = 6.2 Hz, 3H, Me-21_CA_), 0.78 (s, 3H, Me-19_CA_), 0.55 (s, 3H, Me-18_CA_); ^13^C NMR (100 MHz, DMSO-*d*_6_) δ 191.4 (C=O), 173.2 (C=O), 145.7, 131.8, 131.7, 124.8, 116.7, 116.4, 71.5 (C-12_CA_), 70.9 (C-3_CA_), 66.7 (C-7_CA_), 56.1, 46.6, 46.2, 42.0, 41.8, 35.8, 35.6, 35.3, 34.8, 34.6, 32.8, 32.1, 30.8, 29.0, 27.8, 26.7, 23.1 (C-19_CA_), 17.6 (C-21_CA_), 12.8 (C-18_CA_); HRMS (ESI): *m*/*z* [M + H]^+^ calcd for chemical formula: C_35_H_50_N_4_O_5_F^+^ 625.3760 found: 625.3754; Anal. RP-HPLC t_R_ = 4.000 min, purity 94.54%; ${\left[\alpha \right]}_{D}^{20}$ = +22 (*c* 1.0, MeOH).

*(4R)-4-((3R,10S,12S,13R)-3,12-Dihydroxy-10,13-dimethylhexadecahydro-1H-cyclopenta[a]phenanthren-17-yl)-N-((1-(2-(4-fluorophenyl)-2-oxoethyl)-1H-1,2,3-triazol-4-yl)methyl)pentanamide* (**6dd**): Off-white solid; Yield: 77% (0.117 g); mp: 126–127 °C; ^1^H NMR (400 MHz, DMSO-*d_6_*) δ 8.40–8.36 (m, 1H, NH_Amide_), 8.18–8.14 (m, 2H, H_Ar_), 7.83 (s, 1H, H_Triazole_), 7.46 (d, *J* = 8.8 Hz, 2H, H_Ar_), 6.15 (s, 2H), 4.49 (d, *J* = 4.3 Hz, 1H, OH_DCA_), 4.32 (d, *J* = 5.7 Hz, 2H), 4.20 (d, *J* = 4.1 Hz, 1H, OH_DCA_), 3.77 (d, *J* = 3.8 Hz, 1H, H-12_DCA_), 3.43–3.40 (m, 1H, H-3_DCA_), 2.18 – 1.98 (m, 2H), 1.80–1.66 (m, 6H), 1.64–1.44 (m, 5H), 1.35–1.15 (m, 13H), 0.92 (d, *J* = 6.4 Hz, 3H, Me-21_DCA_), 0.82 (s, 3H, Me-19_DCA_), 0.56 (s, 3H, Me-18_DCA_); ^13^C NMR (100 MHz, DMSO-*d*_6_) δ 191.4 (C=O), 173.1 (C=O), 145.7, 131.8, 131.7, 124.8, 116.7, 116.4, 71.5 (C-12_DCA_), 70.4 (C-3_DCA_), 56.1, 47.9, 46.7, 46.4, 42.1, 36.7, 36.1, 35.6, 35.5, 34.6, 34.3, 33.4, 32.8, 32.1, 30.7, 29.1, 27.7, 27.4, 26.6, 24.0, 23.5 (C-19_DCA_), 17.5 (C-21_DCA_), 12.9 (C-18_DCA_); HRMS (ESI): *m/z* [M + H]^+^ calcd for chemical formula: C_35_H_50_N_4_O_4_F^+^ 609.3811 found: 609.3807; Anal. RP-HPLC t_R_ = 4.030 min, purity 95.47%; ${\left[\alpha \right]}_{D}^{20}$ = +60 (*c* 1.0, MeOH).

*(1-(2-(4-Chlorophenyl)-2-oxoethyl)-1H-1,2,3-triazol-4-yl)methyl (4R)-4-((3R,7R,10S,12S,13R)-3,7,12-trihydroxy-10,13-dimethylhexadecahydro-1H-cyclopenta[a]phenanthren-17-yl)pentanoate* (**6ae**): Off-white solid; Yield: 74% (0.106 g); mp: 153–154 °C; ^1^H NMR (400 MHz, DMSO-*d_6_*) δ 8.10 (s, 1H, H_Triazole_), 8.08 (d, *J* = 8.6 Hz, 2H, H_Ar_), 7.70 (d, *J* = 8.6 Hz, 2H, H_Ar_), 6.19 (s, 2H), 5.17 (s, 2H), 4.39 (brs, 1H, OH_CA_), 4.15 (d, *J* = 3.5 Hz, 1H, OH_CA_), 4.05 (d, *J* = 3.3 Hz, 1H, OH_CA_), 3.77 (d, *J* = 3.4 Hz, 1H, H-12_CA_), 3.60 (brs, 1H, H-7_CA_), 3.18 (brs, 1H, H-3_CA_), 2.42–2.24 (m, 2H), 2.22–2.09 (m, 2H), 1.82–1.59 (m, 7H), 1.49–1.14 (m, 13H), 0.91 (d, *J* = 6.1 Hz, 3H, Me-21_CA_), 0.80 (s, 3H, Me-19_CA_), 0.56 (s, 3H, Me-18_CA_); ^13^C NMR (100 MHz, DMSO-*d*_6_) δ 191.7 (C=O), 173.6 (C=O), 142.4, 139.6, 133.3, 130.6, 129.6, 126.8, 71.5 (C-12_CA_), 70.9 (C-3_CA_), 66.7 (C-7_CA_), 57.5, 56.3, 46.5, 46.2, 42.0, 41.8, 35.8, 35.4, 35.3, 34.8, 31.2, 31.1, 30.8, 29.0, 27.7, 26.7, 23.1 (C-19_CA_), 17.4 (C-21_CA_), 12.8 (C-18_CA_); HRMS (ESI): *m*/*z* [M + H]^+^ calcd for chemical formula: C_35_H_49_N_3_O_6_Cl^+^ 642.3304 found: 642.3291; Anal. RP-HPLC t_R_ = 4.107 min, purity 97.01%; ${\left[\alpha \right]}_{D}^{20}$ = +27 (*c* 1.0, MeOH).

*(1-(2-(4-Chlorophenyl)-2-oxoethyl)-1H-1,2,3-triazol-4-yl)methyl (4R)-4-((3R,10S,12S,13R)-3,12-dihydroxy-10,13-dimethylhexadecahydro-1H-cyclopenta[a]phenanthren-17-yl)pentanoate* (**6be**): Off-white solid; Yield: 72% (0.104 g); mp: 92–93 °C; ^1^H NMR (400 MHz, DMSO-*d_6_*) δ 8.10 (d, *J* = 2.8 Hz, 2H, H_Ar_), 8.07 (s, 1H, H_Triazole_), 7.69 (d, *J* = 8.6 Hz, 2H, H_Ar_), 6.19 (s, 2H), 5.17 (s, 2H), 4.52 (d, *J* = 4.2 Hz, 1H, OH_DCA_), 4.22 (d, *J* = 4.1 Hz, 1H, OH_DCA_), 3.78 (d, *J* = 3.7 Hz, 1H, H-12_DCA_), 3.48 (brs, 1H, H-3_DCA_), 2.43–2.15 (m, 2H), 1.82–1.43 (m, 11H), 1.37–1.08 (m, 13H), 0.90 (d, *J* = 6.2 Hz, 3H, Me-21_DCA_), 0.84 (s, 3H, Me-19_DCA_), 0.57 (s, 3H, Me-18_DCA_); ^13^C NMR (100 MHz, DMSO-*d*_6_) δ 191.7 (C=O), 173.6 (C=O), 142.4, 139.6, 133.3, 130.6, 129.6, 126.9, 71.4 (C-12_DCA_), 70.4 (C-3_DCA_), 57.5, 56.3, 47.9, 46.6, 46.5, 42.0, 36.8, 36.1, 35.6, 35.4, 34.3, 33.4, 31.1, 31.1, 29.0, 27.6, 27.4, 26.6, 23.6 (C-19_DCA_), 17.3 (C-21_DCA_), 12.9 (C-18_DCA_); HRMS (ESI): *m*/*z* [M + H]^+^ calcd for chemical formula: C_35_H_49_N_3_O_5_Cl^+^ 626.3355 found: 626.3336; Anal. RP-HPLC t_R_ = 5.343 min, purity 96.73%; ${\left[\alpha \right]}_{D}^{20}$ = +18 (*c* 1.0, MeOH).

*(4R)-N-((1-(2-(4-Chlorophenyl)-2-oxoethyl)-1H-1,2,3-triazol-4-yl)methyl)-4-((3R,7R,10S,12S,13R)-3,7,12-trihydroxy-10,13-dimethylhexadecahydro-1H-cyclopenta[a]phenanthren-17-yl)pentanamide* (**6ce**): Off-white solid; Yield: 78% (0.112 g); mp: 154–155 °C; ^1^H NMR (400 MHz, DMSO-*d*_6_) δ 8.40 (t, *J* = 5.7 Hz, 1H, NH_Amide_), 8.07 (d, *J* = 8.6 Hz, 2H, H_Ar_), 7.83 (s, 1H, H_Triazole_), 7.69 (d, *J* = 8.6 Hz, 2H, H_Ar_), 6.15 (s, 2H), 4.39 (brs, 1H, OH_CA_), 4.32 (d, *J* = 5.7 Hz, 2H), 4.14 (brs, 1H, OH_CA_), 4.05 (brs, 1H, OH_CA_), 3.77 (brs, 1H, H-12_CA_), 3.60 (brs, 1H, H-7_CA_), 3.21–3.17 (m, 1H, H-3_CA_), 2.24–2.16 (m, 2H), 2.14–2.07 (m, 2H), 1.81–1.61 (m, 7H), 1.44–1.20 (m, 13H), 0.92 (d, *J* = 6.2 Hz, 3H, Me-21_CA_), 0.78 (s, 3H, Me-19_CA_), 0.54 (s, 3H, Me-18_CA_); ^13^C NMR (100 MHz, DMSO-*d*_6_) δ 191.8 (C=O), 173.2 (C=O), 145.7, 139.6, 133.3, 130.5, 129.6, 124.8, 71.5 (C-12_CA_), 70.9 (C-3_CA_), 66.7 (C-7_CA_), 56.2, 46.6, 46.2, 42.0, 41.8, 35.8, 35.6, 35.3, 34.9, 34.8, 34.6, 32.8, 32.7, 32.1, 32.0, 30.8, 29.0, 28.2, 27.8, 23.3 (C-19_CA_), 17.5 (C-21_CA_), 12.8 (C-18_CA_); HRMS (ESI): *m*/*z* [M + H]^+^ calcd for chemical formula: C_35_H_50_N_4_O_5_Cl^+^ 641.3464 found: 641.3457; Anal. RP-HPLC t_R_ = 3.607 min, purity 97.57%; ${\left[\alpha \right]}_{D}^{20}$ = +16 (c 1.0, MeOH).

*(4R)-N-((1-(2-(4-Chlorophenyl)-2-oxoethyl)-1H-1,2,3-triazol-4-yl)methyl)-4-((3R,10S,12S,13R)-3,12-dihydroxy-10,13-dimethylhexadecahydro-1H-cyclopenta[a]phenanthren-17-yl)pentanamide* (**6de**): Off-white solid; Yield: 76% (0.110 g); mp: 100–101 °C; ^1^H NMR (400 MHz, DMSO-*d_6_*) δ 8.39 (t, *J* = 5.8 Hz, 1H, NH_Amide_), 8.07 (d, *J* = 8.6 Hz, 2H, H_Ar_), 7.82 (s, 1H, H_Triazole_), 7.69 (d, *J* = 8.6 Hz, 2H, H_Ar_), 6.15 (s, 2H), 4.52 (d, *J* = 4.1 Hz, 1H, OH_DCA_), 4.32 (d, *J* = 5.7 Hz, 2H), 4.22 (d, *J* = 4.2 Hz, 1H, OH_DCA_), 3.77 (brs, 1H, H-12_DCA_), 3.47 (m, 1H, H-3_DCA_), 2.20–2.09 (m, 2H), 2.05–1.98 (m, 2H), 1.78–1.51 (m, 11H), 1.35–1.18 (m, 11H), 0.91 (d, *J* = 6.3 Hz, 3H, Me-21_DCA_), 0.82 (s, 3H, Me-19_DCA_), 0.55 (s, 3H, Me-18_DCA_); ^13^C NMR (100 MHz, DMSO-*d*_6_) δ 192.0 (C=O), 172.9 (C=O), 145.7, 139.6, 133.3, 130.5, 129.6, 124.8, 71.5 (C-12_DCA_), 70.4 (C-3_DCA_), 56.2, 47.9, 46.7, 46.4, 42.1, 36.8, 36.1, 35.6, 35.5, 34.6, 34.3, 33.4, 32.8, 32.1, 30.7, 29.1, 27.7, 27.4, 26.6, 24.0, 23.5 (C-19_DCA_), 17.5 (C-21_DCA_), 12.9 (C-18_DCA_); HRMS (ESI): *m*/*z* [M + H]^+^ calcd for chemical formula: C_35_H_50_N_4_O_4_Cl^+^ 625.3515 found: 625.3492; Anal. RP-HPLC t_R_ = 4.040 min, purity 96.32%; ${\left[\alpha \right]}_{D}^{20}$ = +47 (*c* 1.0, MeOH).

*(1-(2-(4-Bromophenyl)-2-oxoethyl)-1H-1,2,3-triazol-4-yl)methyl (4R)-4-((3R,7R,10S,12S,13R)-3,7,12-trihydroxy-10,13-dimethylhexadecahydro-1H-cyclopenta[a]phenanthren-17-yl)pentanoate* (**6af**): Off-white solid; Yield: 83% (0.127 g); mp: 159–160 °C; ^1^H NMR (400 MHz, DMSO-*d_6_*) δ 8.10 (s, 1H, H_Triazole_), 8.00 (d, *J* = 8.6 Hz, 2H, H_Ar_), 7.84 (d, *J* = 8.6 Hz, 2H, H_Ar_), 6.19 (s, 2H), 5.17 (s, 2H), 4.39 (d, *J* = 3.6 Hz, 1H, OH_CA_), 4.15 (d, *J* = 3.3 Hz, 1H, OH_CA_), 4.05 (d, *J* = 3.1 Hz, 1H, OH_CA_), 3.77 (brs, 1H, H-12_CA_), 3.60 (brs, 1H, H-7_CA_), 3.23–3.16 (m, 1H, H-3_CA_), 2.42–2.28 (m, 2H), 2.25–2.09 (m, 2H), 1.81–1.60 (m, 7H), 1.47–1.15 (m, 13H), 0.91 (d, *J* = 6.1 Hz, 3H, Me-21_CA_), 0.80 (s, 3H, Me-19_CA_), 0.56 (s, 3H, Me-18_CA_); ^13^C NMR (100 MHz, DMSO-*d*_6_) δ 191.9 (C=O), 173.6 (C=O), 142.4, 133.6, 132.5, 130.6, 128.9, 126.9, 71.4 (C-12_CA_), 70.9 (C-3_CA_), 66.7 (C-7_CA_), 57.5, 56.3, 46.5, 46.2, 42.0, 41.8, 35.8, 35.5, 35.3, 34.8, 31.1, 30.9, 29.0, 27.7, 26.7, 23.3, 23.1 (C-19_CA_), 17.3 (C-21_CA_), 12.8 (C-18_CA_); HRMS (ESI): *m*/*z* [M + H]^+^ calcd for chemical formula: C_35_H_49_N_3_O_6_Br^+^ 686.2799 found: 686.2780; Anal. RP-HPLC t_R_ = 4.197 min, purity 96.62%; ${\left[\alpha \right]}_{D}^{20}$ = +11 (*c* 1.0, MeOH).

*(1-(2-(4-Bromophenyl)-2-oxoethyl)-1H-1,2,3-triazol-4-yl)methyl (4R)-4-((3R,10S,12S,13R)-3,12-dihydroxy-10,13-dimethylhexadecahydro-1H-cyclopenta[a]phenanthren-17-yl)pentanoate* (**6bf**): Off-white solid; Yield: 80% (0.124 g); mp: 176–177 °C; ^1^H NMR (400 MHz, DMSO-*d_6_*) δ 8.10 (s, 1H, H_Triazole_), 8.00 (d, *J* = 8.7 Hz, 2H, H_Ar_), 7.84 (d, *J* = 8.6 Hz, 2H, H_Ar_), 6.19 (s, 2H), 5.17 (s, 2H), 4.50 (d, *J* = 4.3 Hz, 1H, OH_DCA_), 4.23 (d, *J* = 4.1 Hz, 1H, OH_DCA_), 3.78 (d, *J* = 3.8 Hz, 1H, H-12_DCA_), 3.43 (brs, 1H, H-3_DCA_), 2.40–2.20 (m, 2H), 1.80–1.44 (m, 10H), 1.36–1.16 (m, 14H), 0.90 (d, *J* = 6.2 Hz, 3H, Me-21_DCA_), 0.84 (s, 3H, Me-19_DCA_), 0.57 (s, 3H, Me-18_DCA_); ^13^C NMR (100 MHz, DMSO-*d*_6_) δ 191.9 (C=O), 173.6 (C=O), 142.4, 133.6, 132.5, 130.6, 128.9, 126.9, 71.5 (C-12_DCA_), 70.4 (C-3_DCA_), 57.5, 56.3, 47.9, 46.6, 46.5, 42.1, 36.7, 36.1, 35.6, 35.4, 34.3, 33.4, 33.1, 31.1, 30.7, 29.0, 27.6, 27.4, 26.6, 24.0, 23.6 (C-19_DCA_), 17.3 (C-21_DCA_), 12.9 (C-18_DCA_); HRMS (ESI): *m*/*z* [M + H]^+^ calcd for chemical formula: C_35_H_49_N_3_O_5_Br^+^ 670.2850 found: 670.2855; Anal. RP-HPLC t_R_ = 5.507 min, purity 98.37%; ${\left[\alpha \right]}_{D}^{20}$ = +81 (*c* 1.0, MeOH).

*(4R)-N-((1-(2-(4-Bromophenyl)-2-oxoethyl)-1H-1,2,3-triazol-4-yl)methyl)-4-((3R,7R,10S,12S,13R)-3,7,12-trihydroxy-10,13-dimethylhexadecahydro-1H-cyclopenta[a]phenanthren-17-yl)pentanamide* (**6cf**): Off-white solid; Yield: 81% (0.124 g); mp: 144–145 °C; ^1^H NMR (400 MHz, DMSO-*d_6_*) δ 8.39 (t, *J* = 5.8 Hz, 1H, NH_Amide_), 7.99 (d, *J* = 8.3 Hz, 2H, H_Ar_), 7.86–7.81 (m, 3H, H_Triazole_&H_Ar_), 6.15 (s, 2H), 4.36 (d, *J* = 4.3 Hz, 1H, OH_CA_), 4.32 (d, *J* = 5.7 Hz, 2H), 4.12 (d, *J* = 3.5 Hz, 1H, OH_CA_), 4.03 (d, *J* = 3.4 Hz, 1H, OH_CA_), 3.79–3.76 (m, 1H, H-12_CA_), 3.60 (brs, 1H, H-7_CA_), 3.21–3.15 (m, 1H, H-3_CA_), 2.18–2.12 (m, 2H), 2.04–1.94 (m, 2H), 1.83–1.58 (m, 8H), 1.50–1.15 (m, 12H), 0.92 (d, *J* = 6.2 Hz, 3H, Me-21_CA_), 0.78 (s, 3H, Me-19_CA_), 0.55 (s, 3H, Me-18_CA_); ^13^C NMR (100 MHz, DMSO-*d*_6_) δ 192.1 (C=O), 173.1 (C=O), 145.7, 133.6, 132.5, 130.6, 128.8, 124.8, 71.5 (C-12_CA_), 70.9 (C-3_CA_), 66.7 (C-7_CA_), 56.2, 46.6, 46.2, 42.0, 41.8, 35.6, 35.3, 34.8, 34.6, 32.8, 32.1, 30.9, 29.0, 27.8, 26.7, 23.3, 23.0 (C-19_CA_), 17.5 (C-21_CA_), 12.8 (C-18_CA_); HRMS (ESI): *m*/*z* [M + H]^+^ calcd for chemical formula: C_35_H_50_N_4_O_5_Br^+^ 685.2959 found: 685.2956; Anal. RP-HPLC t_R_ = 3.720 min, purity 96.70%; ${\left[\alpha \right]}_{D}^{20}$ = +13 (*c* 1.0, MeOH).

*(4R)-N-((1-(2-(4-Bromophenyl)-2-oxoethyl)-1H-1,2,3-triazol-4-yl)methyl)-4-((3R,10S,12S,13R)-3,12-dihydroxy-10,13-dimethylhexadecahydro-1H-cyclopenta[a]phenanthren-17-yl)pentanamide* (**6df**): Off-white solid; Yield: 79% (0.122 g); mp: 135–136 °C; ^1^H NMR (400 MHz, DMSO-*d_6_*) δ 8.36 (t, *J* = 5.7 Hz, 1H, NH_Amide_), 7.99 (d, *J* = 8.6 Hz, 2H, H_Ar_), 7.88–7.80 (m, 3H, H_Triazole_&H_Ar_), 6.14 (s, 2H), 4.48 (d, *J* = 4.2 Hz, 1H, OH_DCA_), 4.32 (d, *J* = 5.7 Hz, 2H), 4.19 (d, *J* = 4.1 Hz, 1H, OH_DCA_), 3.78 (brs, 1H, H-12_DCA_), 3.39 (brs, 1H, H-3_DCA_), 2.15–2.00 (m, 2H), 1.77–1.47 (m, 11H), 1.36–1.16 (m, 13H), 0.91 (d, *J* = 6.3 Hz, 3H, Me-21_DCA_), 0.82 (s, 3H, Me-19_DCA_), 0.55 (s, 3H, Me-18_DCA_); ^13^C NMR (100 MHz, DMSO-*d*_6_) δ 192.1 (C=O), 173.2 (C=O), 145.7, 133.6, 132.5, 130.6, 128.8, 124.8, 71.5 (C-12_DCA_), 70.9 (C-3_DCA_), 56.2, 46.6, 46.2, 42.0, 41.8, 35.7, 35.6, 35.3, 34.8, 32.8, 30.8, 27.8, 26.7, 23.1 (C-19_DCA_), 17.5 (C-21_DCA_), 12.8 (C-18_DCA_); HRMS (ESI): *m*/*z* [M + H]^+^ calcd for chemical formula: C_35_H_50_N_4_O_4_Br^+^ 669.3010 found: 669.3003; Anal. RP-HPLC t_R_ = 3.937 min, purity 98.65%; ${\left[\alpha \right]}_{D}^{20}$ = +17 (*c* 1.0, MeOH).

### 3.3. Biological Assay

#### 3.3.1. Cell Culture and MTT Assay

The cytotoxicity activity of all the conjugates (**6aa**–**6df**) was evaluated in vitro by MTT assay against two different breast cancer cell lines 4T1 (murine) and MCF-7 (human) and HEK 293 (human) as normal cell line. DTX was used as positive control. Cells were grown in DMEM supplemented with 10% FBS and 1% antibiotic solution and incubated at 5% CO_2_ and 37 °C for 24 h. The stock solutions of all the conjugates were prepared in DMSO and diluted for further use. Briefly, 5 × 10^3^ cells/well were seeded in 96-well cell culture plates and allowed to adhere for 24 h. Cell inhibition (%) was determined after 48 h exposure to the compounds at 1–25 μM concentration. After 48 h, MTT assay was performed and the yellow tetrazolium salt (MTT) was reduced in metabolically active cells to form insoluble purple formazan crystals, which were solubilized by the addition of DMSO. The optical density (OD) was recorded at 560 nm and 630 nm as reference wavelength. Percentage cell inhibition was determined by comparison with untreated cells [50,51].

#### 3.3.2. Apoptotic Study

The extent of apoptosis induced by compounds **6af** and **6cf** in MCF-7 and 4T1 cells was quantified by flow cytometry according to the manufacturer’s protocol. Briefly, cells were seeded in a 6-well plate at a cell density of 1 × 10^6^ cells/well. After 24 h, the media was discarded and cells were treated with fresh media containing compounds **6af** and **6cf** at their respective IC_50_ concentrations for 48 h. After treatment, cells were trypsinized, harvested in PBS and collected by centrifugation for 5 min at 2000 rpm. Cells were then resuspended in 1X binding buffer and stained with FITC-labeled Annexin V Alexa Fluor 488 (5 μL) and propidium iodide (10 μL). Cells were analyzed using flow cytometer (Beckman Coulter), and data were analyzed with CytExpert software.

#### 3.3.3. Pharmacokinetic Study of **6cf**

Wistar rats (male; 8–10 weeks, 200–240 g) were procured from Central Animal Facility, BITS Pilani (Pilani, India). Animal experiment protocol was approved by Institutional Animal Ethics Committee (IAEC/RES/24/03), BITS Pilani, Pilani, and all experiments were conducted as per CPCSEA guidelines. Rats were housed in well-ventilated cages at standard laboratory conditions with regular light/dark cycles for 12 h and fed with standard normal diet ab libitum.

The pharmacokinetic study of **6cf** was performed on Wistar rats. **6cf** solution (prepared in normal saline with 5% *w*/*v* tween 80) was administered intravenously at the dose of 10 mg/kg with maximum dosing volume of 300 µL to each rat without fasting (*n* = 4). After i.v. dosing, blood samples were collected for each preset time point at 10, 20, 30, 50 min, 1.5, 2, 4, 6, 8, 12 and 24 h. **6cf** plasma concentration–time profile was plotted and analyzed by non-compartmental model approach using Phoenix 2.1 WinNonlin (Pharsight Corporation, USA) to determine t_1/2_, elimination half-life; C_0_, drug concentration in plasma at t = 0; AUC_0-t_, area under curve from zero to the last time point; AUC_0–∞_, area under curve from zero to infinity; and MRT, mean residence time.

#### 3.3.4. Determination of **6cf** in Rat Plasma

A simple liquid–liquid extraction (LLE) method was used for extraction of **6cf** from the rat plasma. A 200 µL aliquot of plasma sample containing **6cf** was taken in 5 mL glass tube, followed by the addition of 100 µL of internal standard (I.S.) (clobetasol, 2 µg/mL) solution. Samples were vortexed for 1 min, and then 2 mL of ethyl acetate was added as extracting solvent. The samples were vortexed for 5 min and centrifuged at 5000 rpm for 15 min at 4 °C. The organic layer was collected and evaporated to dryness at 40 ± 0.5 °C. The residue was reconstituted with 250 µL of mobile phase and vortexed for 1 min. Finally, 150 µL of sample was injected into HPLC for quantification.

#### 3.3.5. Liquid Chromatographic Conditions

A Thermo Fisher Rapid Separation (RS) UHPLC System (Ultimate 3000) equipped with a pump (LPG-3400SD), Diode Array Detector (DAD) (DAD-3000) and autosampler (ACC-3000T) with 250 µL injection loop was used for purity analysis. The UHPLC system was equilibrated for approximately 40 min before beginning the sample analysis. Column temperature was 35° throughout the analysis. **6cf** and I.S. were separated on Intersil^®^ ODS (C18) column (250 × 4.6 mm, 5µm) with a mobile phase consisting of acetonitrile:water (60:40 % *v*/*v*) run in isocratic mode at a flow rate of 1 mL/min, detection wavelength 259 nm and injection volume of 150 µL. Retention time was found to be 6.2 and 12.2 min for **6cf** and clobetasol (I.S.), respectively. Control of hardware and data handling was performed using Chromeleon software version 7.2 SR4.

## 4. Conclusions

In summary, we synthesized a series of cholic-acid- and deoxycholic-acid-appended triazolyl aryl ketones in excellent yields via a Cu-catalyzed multi-component approach. All the synthesized conjugates were evaluated for their cytotoxicity against human breast adenocarcinoma (MCF-7) and mouse mammary carcinoma (4T1) cells at 10 µM, which highlighted three conjugates (**6af**, **6bf**, **6cf**) displaying interesting anticancer activity with IC_50_ values less than 19 µM on both tested cancer cell lines. Among these, the cholic-acid-appended triazolyl 4-bromophenyl ketone (**6cf**) connected via an amide bond was found to be active against both cancer cell lines with IC_50_ values of 5.71 µM and 8.71 µM, respectively, as compared to the reference drug possessing an IC_50_ value of 9.46 µM and 13.85 µM, respectively. Meanwhile, cholic-acid-appended triazolyl 4-bromophenyl ketone connected via an ester bond (**6af**) was found to be active against both cancer cell lines with IC_50_ values of 2.61 µM and 12.84 µM, respectively. Most of the conjugates showed low cytotoxicity toward the normal human embryonic kidney cell line (HEK 293) as evident from their cell viability data. Apoptosis studies of **6af** and **6cf** on MCF-7 cells at their respective IC_50_ values indicated induction of higher apoptosis by **6cf** in comparison to **6af** (46.09% vs. 33.89%). Meanwhile, in 4T1 cells, a similar apoptotic potential of the two compounds contributing to a total apoptosis of 19.02% and 19.56% in 4T1 cells was observed. Additionally, an MRT of 8.47 h with a half-life of 5.63 h was observed by in vivo pharmacokinetics studies of **6cf** in rats. In light of the present work, it appears that cytotoxicity is not only driven by the nature of the bile acid, but also by the electronic effect of the substituent present on the aryl moiety of aryl ketones. Clearly, the results suggest the potential of the studied conjugates in the development of anticancer drug candidates.

## Figures and Tables

**Table 1:** ^1^H and ^13^C NMR assignments for the representative proton/carbon signals of **6aa**.

S. No.	Labeling	^1^H NMR	^13^C NMR
1.	18	0.57 (s, 3H)	12.8
2.	19	0.80 (s, 3H)	23.1
3.	21	0.92 (d, *J* = 6.2 Hz, 3H)	17.4
4.	3	3.17 (d, *J* = 5.1 Hz, 1H)	70.9
5.	7	3.61 (brs, 1H)	66.7
6.	12	3.78 (d, *J* = 3.8 Hz, 1H)	71.5
7.	OH	4.33 (d, *J* = 4.3 Hz, 1H)	-
8.	OH	4.12 (d, *J* = 3.8 Hz, 1H)	-
9.	OH	4.01 (d, *J* = 3.3 Hz, 1H)	-
10.	25 (COO-CH_2_)	5.18 (s, 2H)	57.5
11.	28 (N-CH_2_)	6.20 (s, 2H)	56.3
12.	27 (Triazole-H)	8.11 (s, 1H)	126.9
13.	24 (COO)	-	173.6
14.	29 (CO)	-	192.5

**Table 2:** In vitro cytotoxicity of compounds (**6aa**–**6df**) against two different cancer cell lines and normal human kidney cell line.

Com. No.	% Cell Viability at 10 µM			milog P ^d^	TPSA ^e^	% ABS ^f^	Drug-Likeness Score
	MCF-7 ^a^	4T1 ^b^	HEK 293 ^c^				
**6aa**	77.32	74.32	86.08	4.54	134.78	62.50	0.75
**6ba**	91.90	97.81	88.92	5.45	114.55	69.48	0.65
**6ca**	92.06	85.16	90.13	3.89	137.57	61.53	0.69
**6da**	91.20	99.56	97.86	4.81	117.34	68.51	0.60
**6ab**	57.35	72.93	87.63	4.99	134.78	62.50	0.82
**6bb**	67.60	85.69	89.17	5.90	114.55	69.48	0.72
**6cb**	61.86	96.74	86.00	4.34	137.57	61.53	0.75
**6db**	78.69	53.67	89.43	5.26	117.34	68.51	0.66
**6ac**	55.59	80.02	87.88	4.59	144.01	59.31	0.98
**6bc**	69.62	68.40	90.77	5.51	123.78	66.29	0.89
**6cc**	95.36	56.79	96.75	3.95	146.81	58.35	0.96
**6dc**	68.52	64.92	86.53	4.87	126.58	65.32	0.88
**6ad**	66.48	47.70	86.79	4.70	134.78	62.50	1.07
**6bd**	79.62	52.35	89.37	5.62	114.55	69.48	0.97
**6cd**	88.72	70.65	97.51	4.06	137.57	61.53	1.01
**6dd**	73.76	89.90	81.61	4.97	117.34	68.51	0.92
**6ae**	79.62	58.92	96.21	5.21	134.78	62.50	1.14
**6be**	68.52	76.01	88.37	6.13	114.55	69.48	1.05
**6ce**	69.16	50.59	92.69	4.57	137.57	61.53	1.09
**6de**	86.85	62.89	90.45	5.49	117.34	68.50	1.01
**6af**	26.52	55.91	88.20	5.34	134.78	62.50	0.94
**6bf**	44.43	60.86	86.82	6.26	114.55	69.48	0.85
**6cf**	37.53	49.27	87.82	4.70	137.57	61.53	0.88
**6df**	86.45	48.38	93.46	5.62	117.34	68.51	0.79
**DTX** ^g^	46.47	56.88	-	-	-	-	-

^a^ Human breast adenocarcinoma, ^b^ Mouse mammary carcinoma, ^c^ Human embryonic kidney 293 cells, ^d^ Logarithm of compound partition coefficient between n-octanol and water, ^e^ Topological polar surface area, ^f^ Percentage absorption calculated using the formula %ABS = 109 − (0.345 × TPSA), ^g^ Docetaxel.

**Table 3:** IC_50_ (µM) values of the active compounds in two different cancer cell lines.

Compound	IC_50_ (µM)	
	MCF-7 ^a^	4T1 ^b^
**6af**	2.61 ± 0.70	12.84 ± 1.80
**6bf**	18.26 ± 1.48	9.68 ± 1.59
**6cf**	5.71 ± 1.00	8.76 ± 1.29
**DTX**	9.46 ± 0.98	13.85 ± 1.07

^a^ Human breast adenocarcinoma, ^b^ Mouse mammary carcinoma.

**Table 4:** The non-compartmental pharmacokinetic parameters for **6cf** in rat plasma after i.v. bolus at dose of 10 mg/kg administration to rat.

Parameters	6cf (Mean ± SEM)
Initial Concentration, C_0_ (ng/mL)	1752.69 ± 66.52
Half-Life, t_1/2_ (h)	5.63 ± 0.539
Elimination Rate Constant, Ke (1/h)	0.13 ± 0.01
Area Under the Curve (0 to 12 h), AUC_0–last_ (ng.h/mL)	1995.306 ± 87.43
Area Under the Curve (0 to infinity), AUC_0–∞_ (ng.h/mL)	2690.50 ± 113.20
Area Under the First Moment Curve (0 to 12 h), AUMC_0–last_ (ng.h/mL)	8155.94 ± 311.78
Area Under the First Moment Curve (0 to infinity), AUMC_0–∞_ (ng.h/mL)	22276.39 ± 2023.334
Mean Residence Time, MRT (h)	8.47 ± 0.96

## Data Availability

Not Applicable.

## Supplementary Materials

Original ^1^H and ^13^C NMR spectra of **4c**,**d** and **6aa**–**6df**, COSY, HSQC and HMBC spectra of **6aa**, HRMS spectra of **6aa**–**6df** and HPLC chromatogram of **6aa**–**6df**.

## Sample Availability

Samples of the compounds **6aa**–**df** are available from the authors.
