# Supplementary material for: Bile-Acid-Appended Triazolyl Aryl Ketones: Design, Synthesis, In Vitro Anticancer Activity and Pharmacokinetics in Rats
Source: Molecules. 2021 Sep 22;26(19):5741. doi: 10.3390/molecules26195741 (PMC8510344; doi:10.3390/molecules26195741)
Supplement: Supplementary file 1 [file molecules-26-05741-s001.zip › molecules-1337866-supplementary.pdf]

Bile-Acid-Appended Triazolyl Aryl Ketones: Design, Synthesis, In Vitro Anticancer Activity and Pharmacokinetics in Rats

Devesh S. Agarwal 1, Samrat Mazumdar 2, Kishan S. Italiya 2, Deepak Chitkara 2 and Rajeev Sakhuja 1,*

1 *Department of Chemistry, Birla Institute of Technology and Science, Pilani 333 031, Rajasthan, India; deveshagarwal88@gmail.com*

2 *Department of Pharmacy, Birla Institute of Technology and Science Pilani 333 031, Rajasthan, India; samratm64@gmail.com (S.M.); italiyakishan2010@gmail.com (K.S.I.); deepak.chitkara@pilani.bits-pilani.ac.in (D.C.)*

* *Correspondence: rajeev.sakhuja@pilani.bits-pilani.ac.in*

S. No.	Contents	Page No.
1.	^{1}H NMR & ^{13}C NMR Spectra of **4c-d**	S2-S3
2.	^{1}H NMR & ^{13}C NMR Spectra of **6aa-6df**	S4-S27
3.	COSY and HSQC spectra of **6aa**	S28
4.	HMBC of **6aa**	S29
5.	HRMS of spectra **6aa-6df**	S30-S35
6.	HPLC chromatogram of **6aa-6df**	S36-S47

^{1}H NMR of 4c

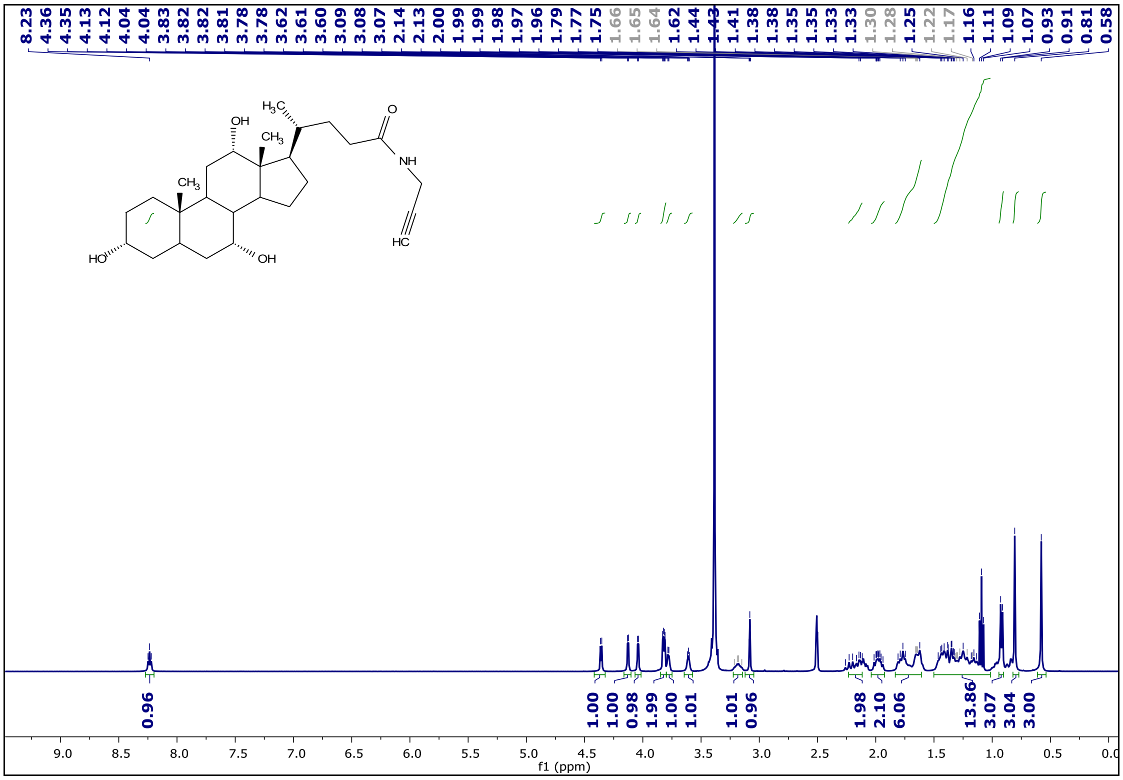

^{13}C NMR of 4c

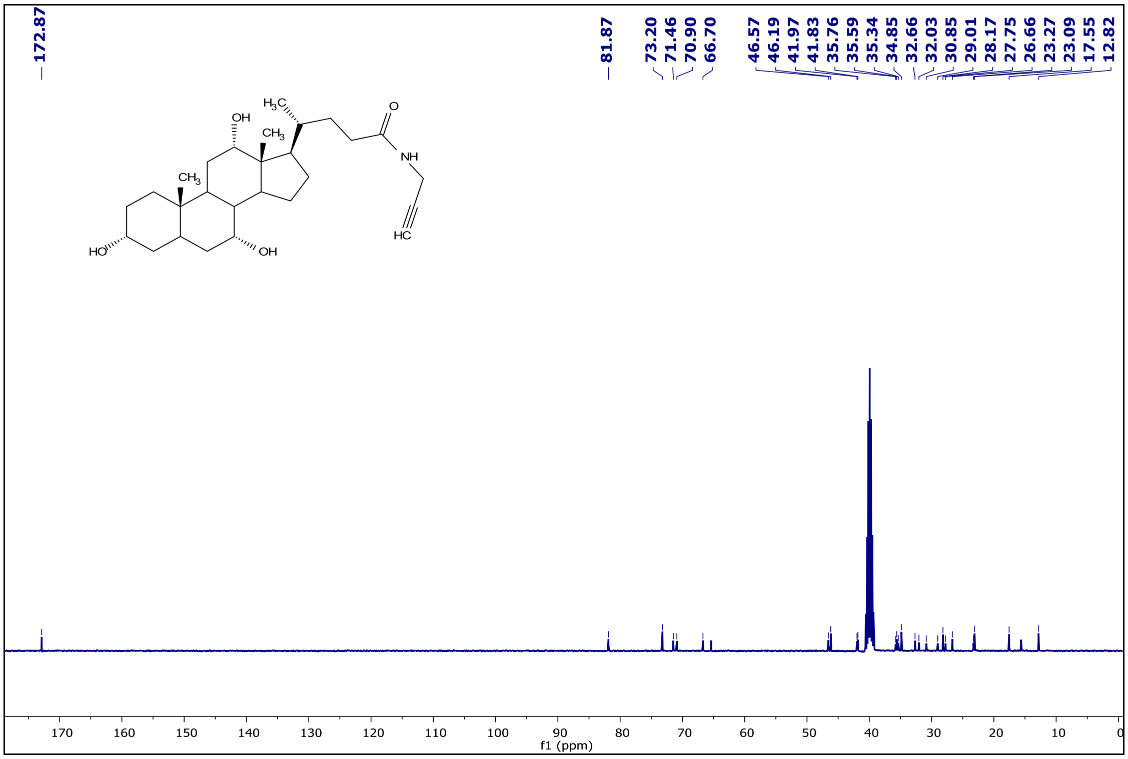

^{1}H NMR of 4d

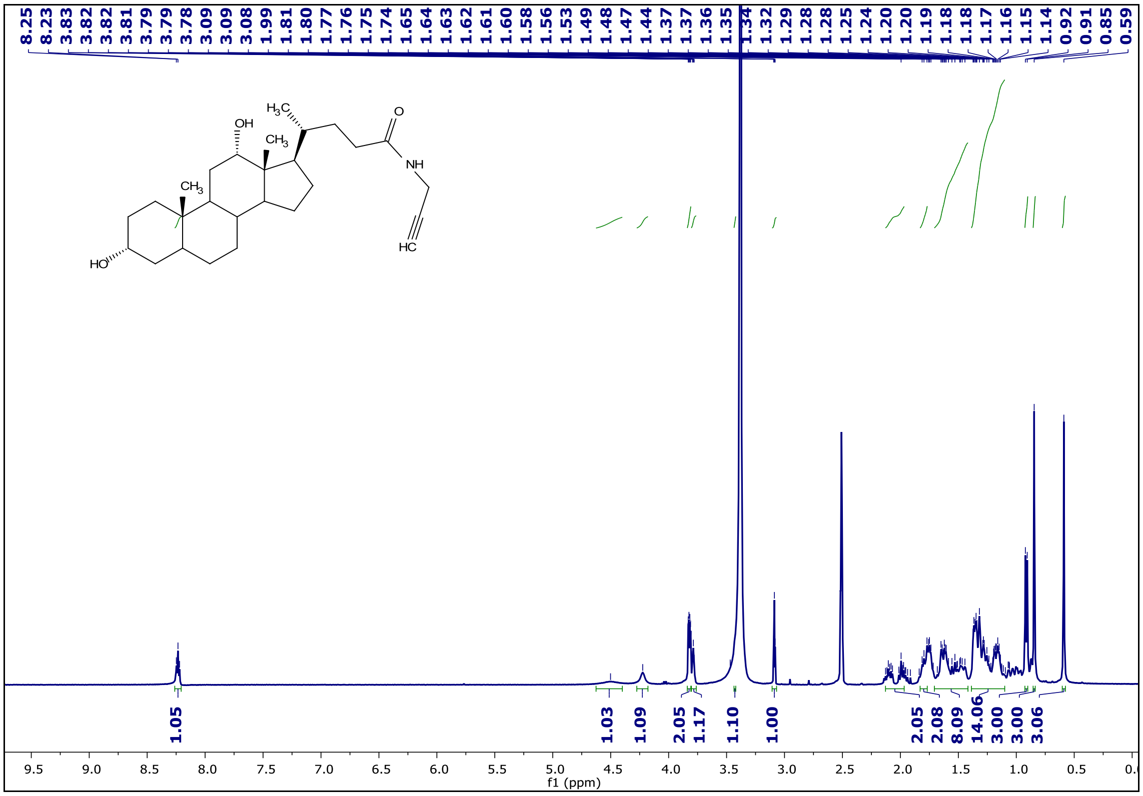

^{13}C NMR of 4d

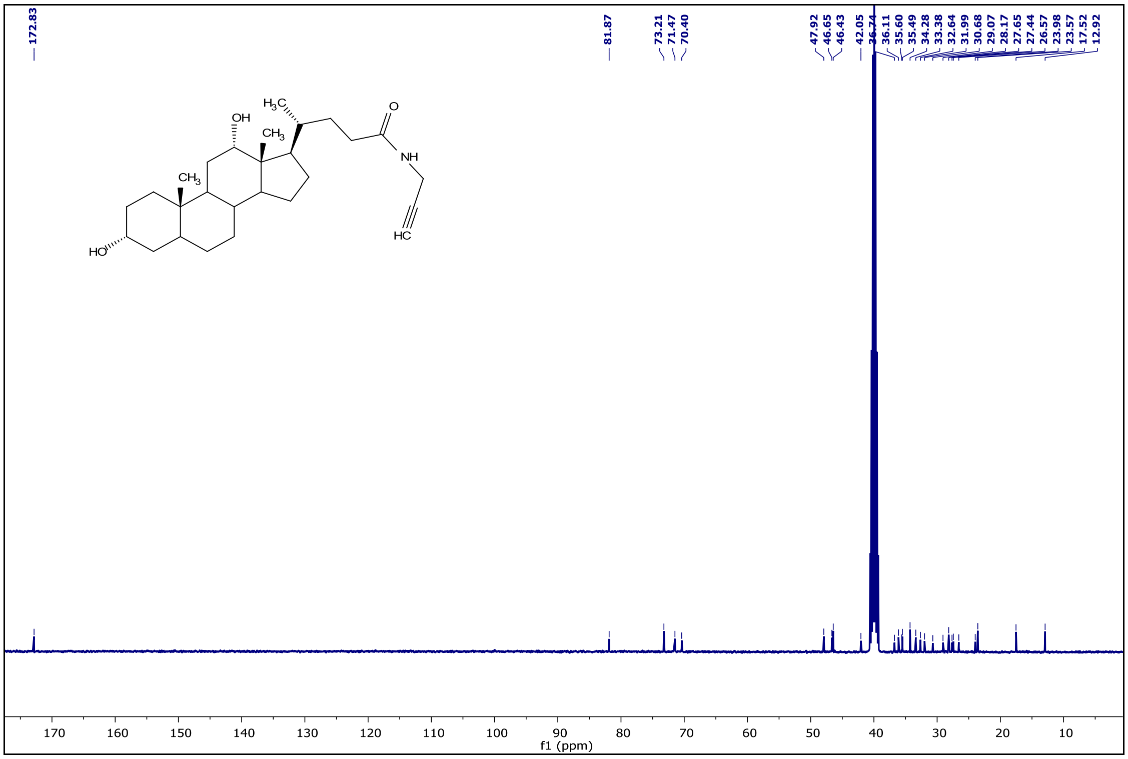

^{1}H NMR of 6aa

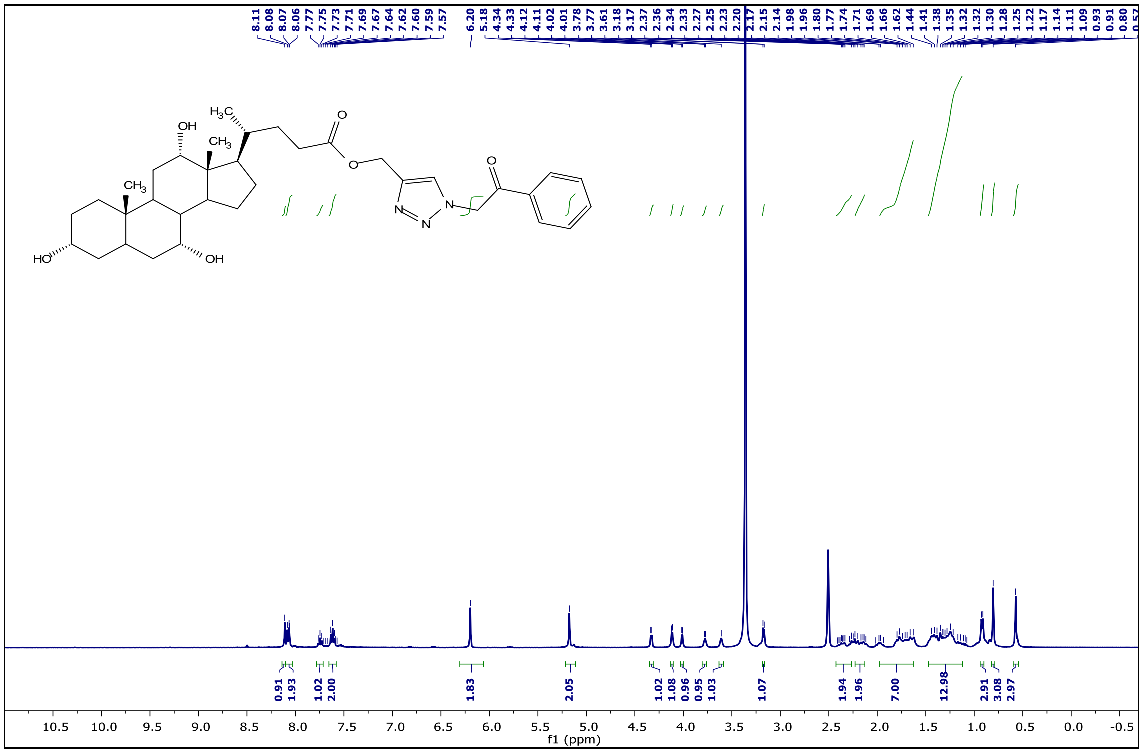

^{13}C NMR of 6aa

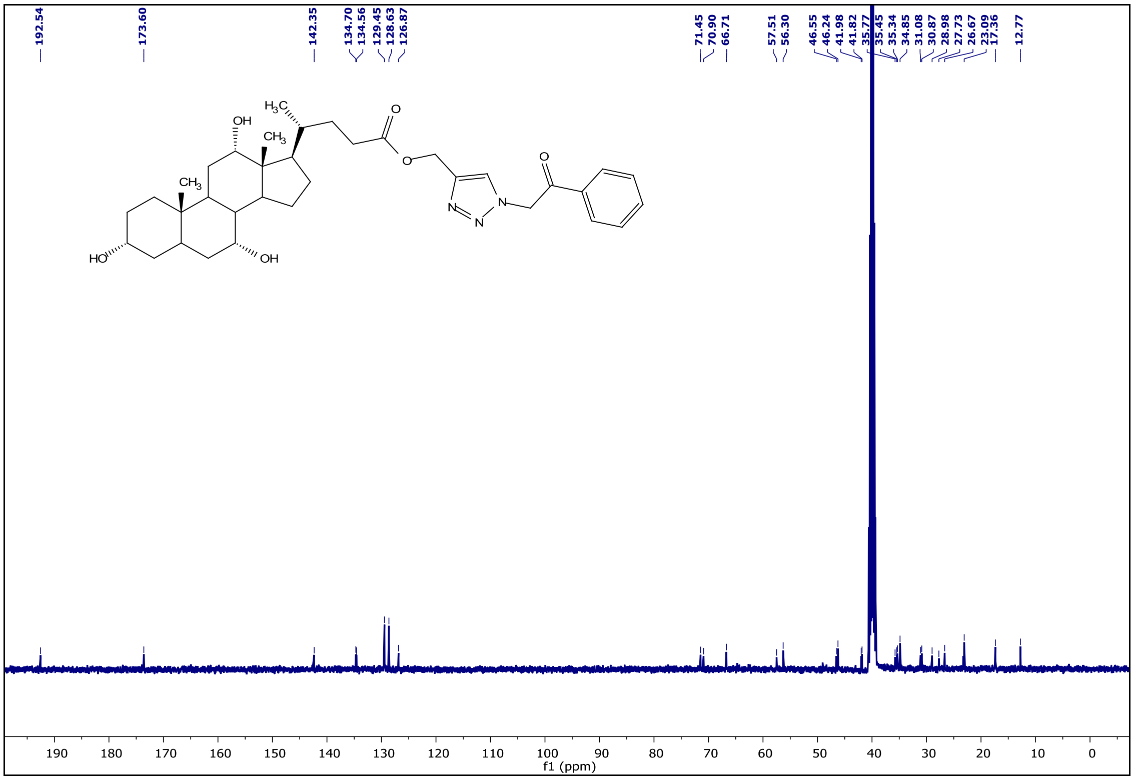

^{1}H NMR of 6ba

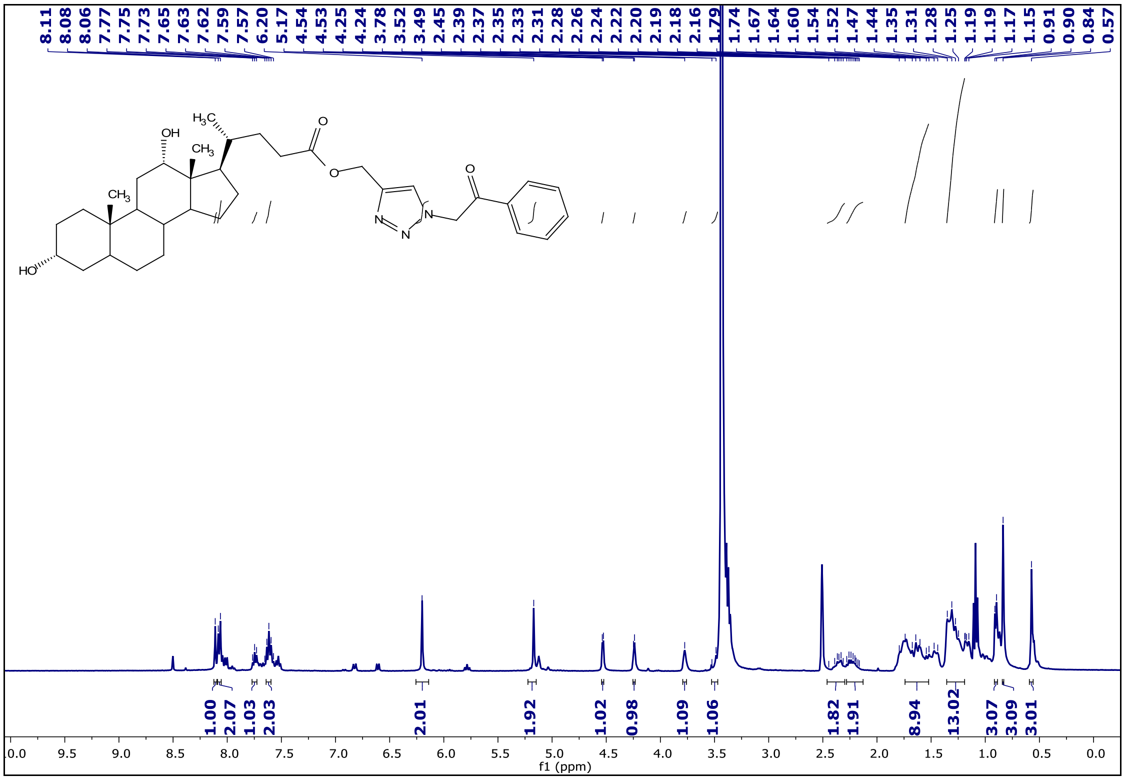

^{13}C NMR of 6ba

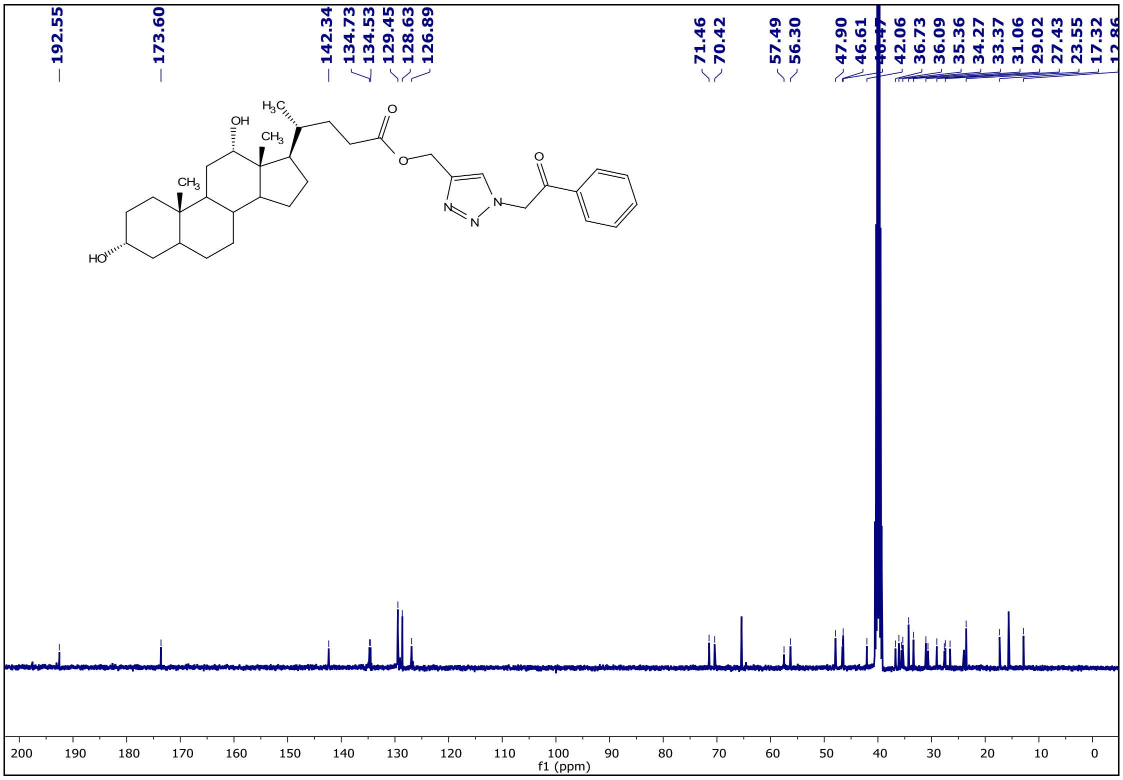

^{1}H NMR of 6ca

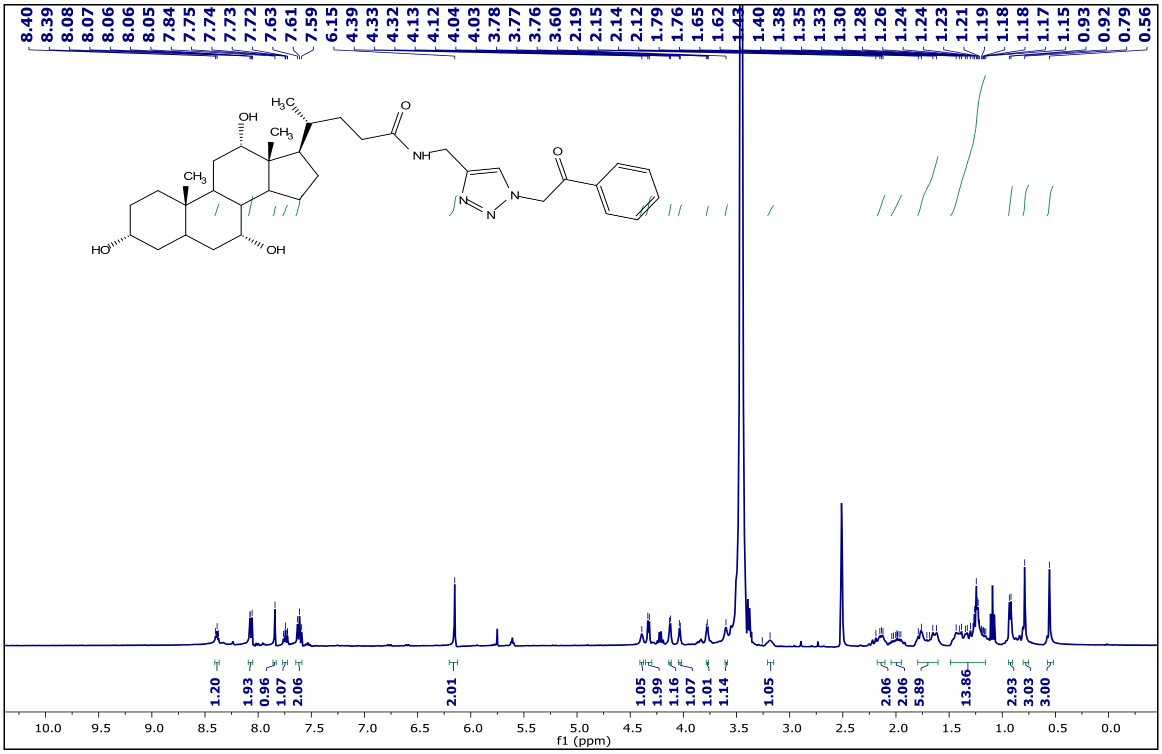

^{13}C NMR of 6ca

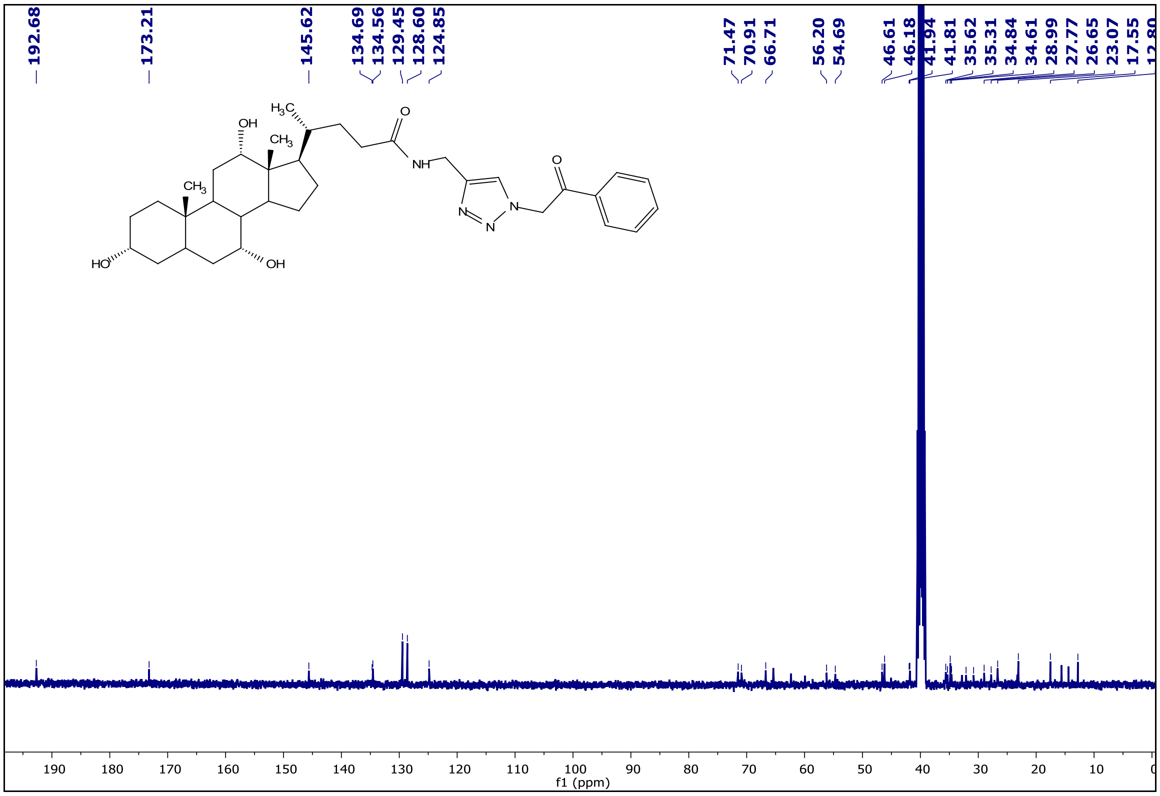

^{1}H NMR of 6da

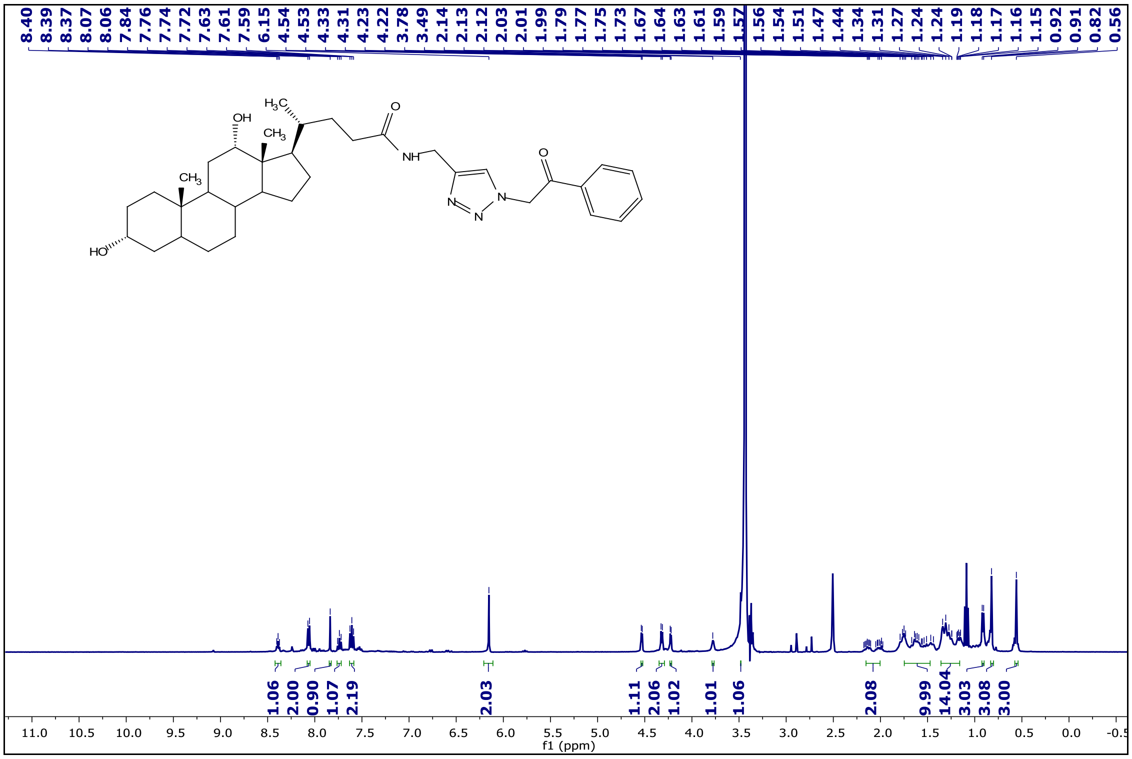

^{13}C NMR of 6da

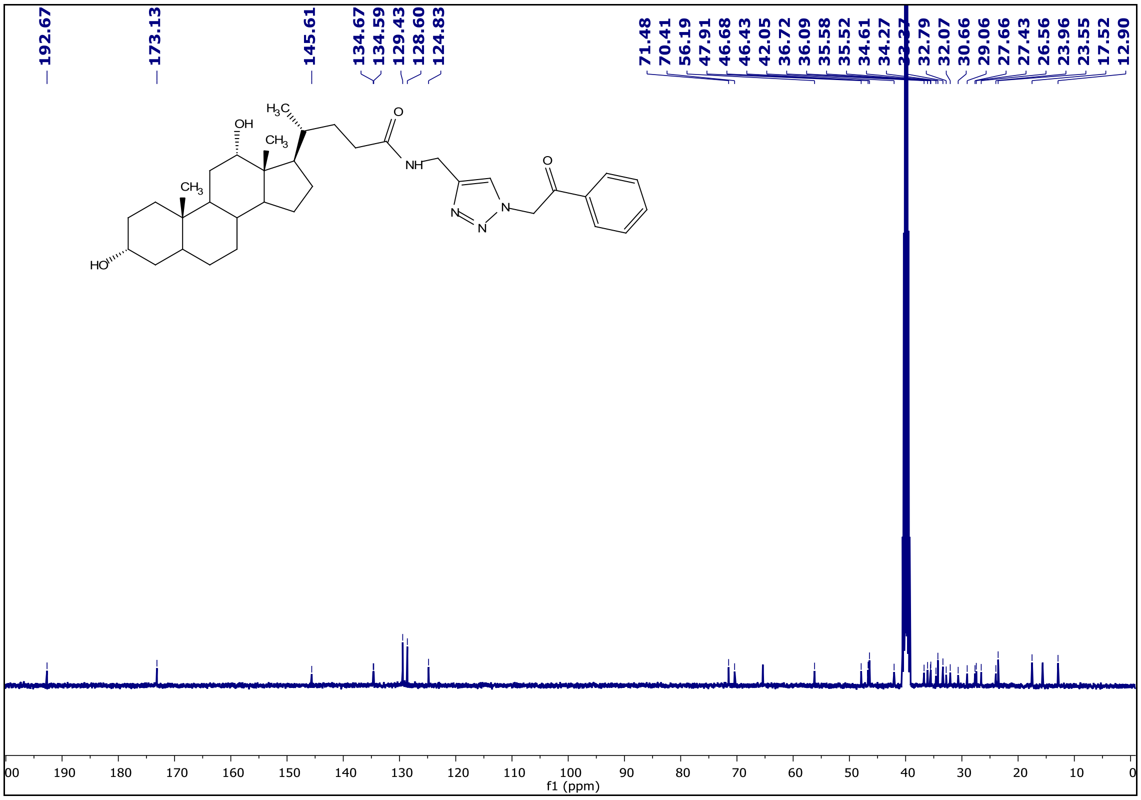

^{1}H NMR of 6ab

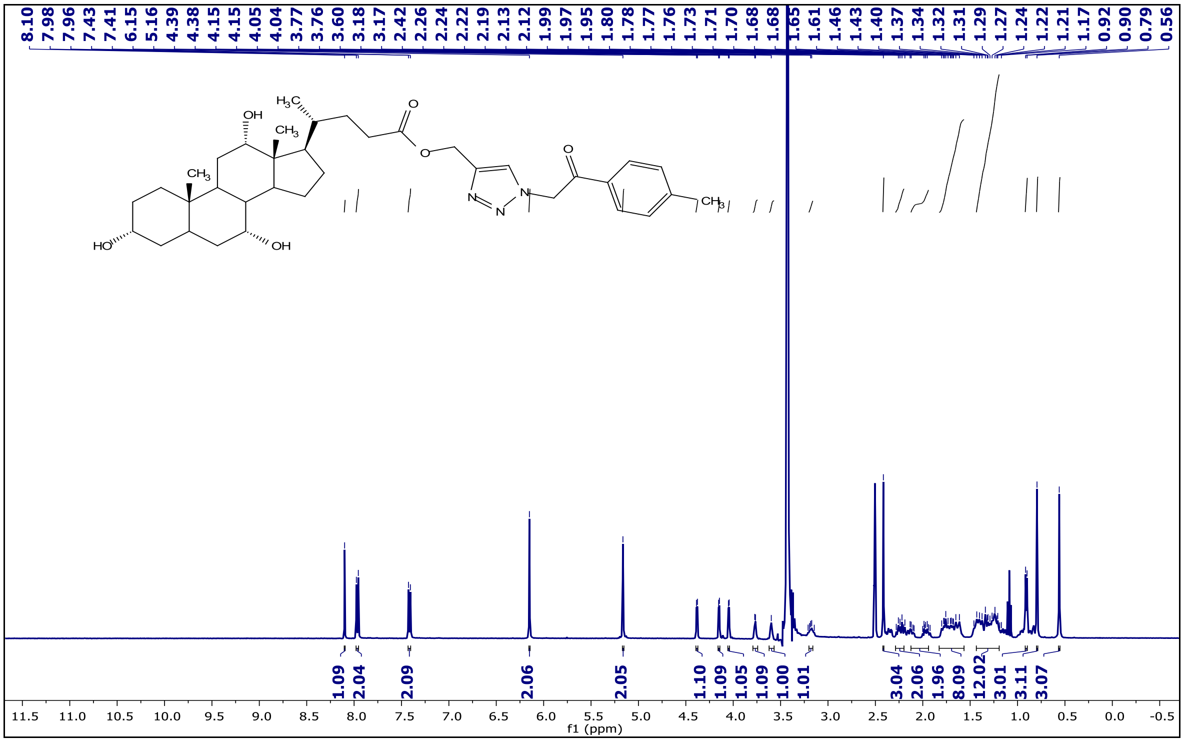

^{13}C NMR of 6ab

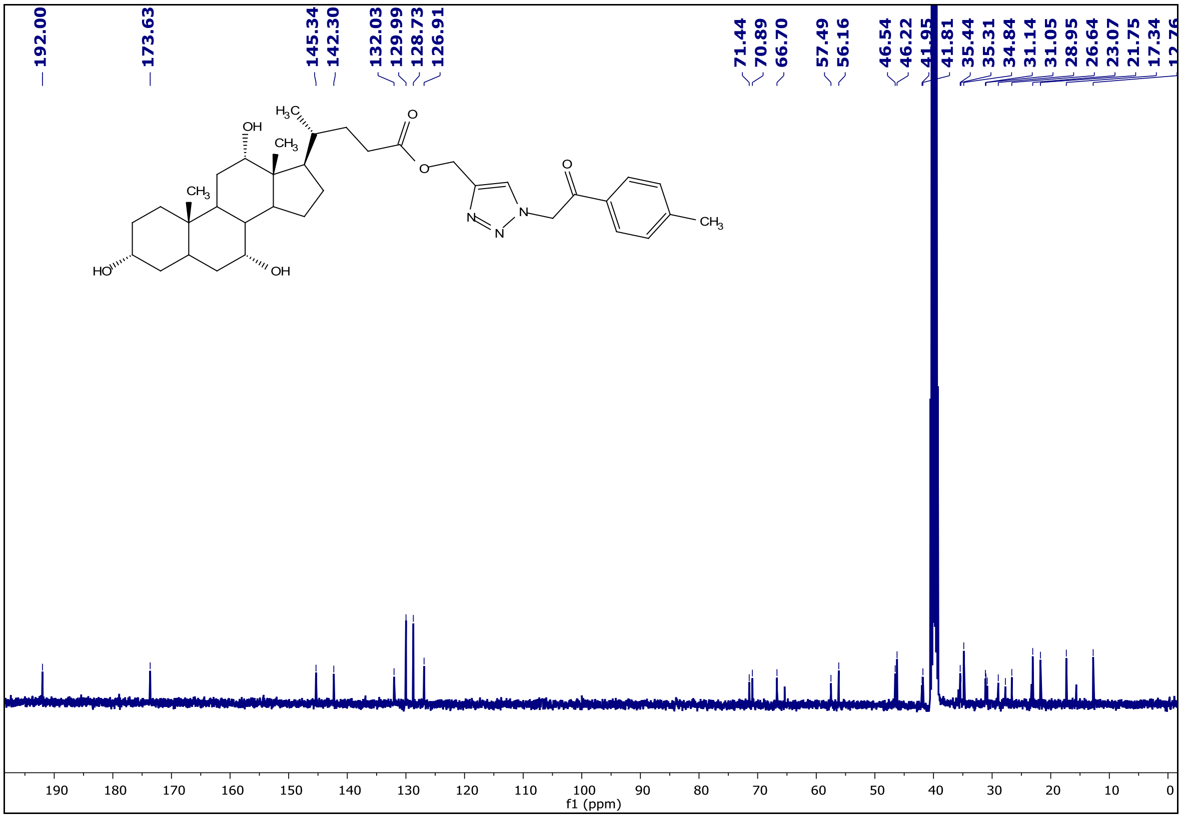

^{1}H NMR of 6bb

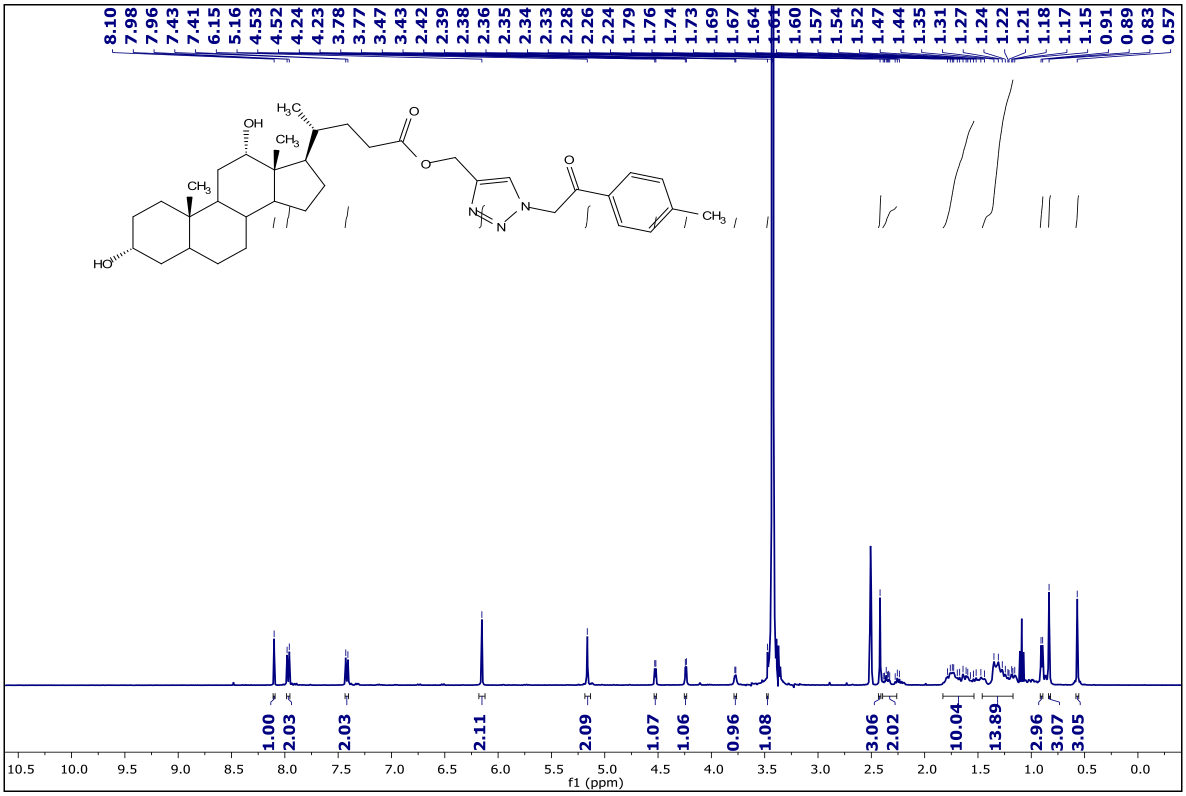

^{13}C NMR of 6bb

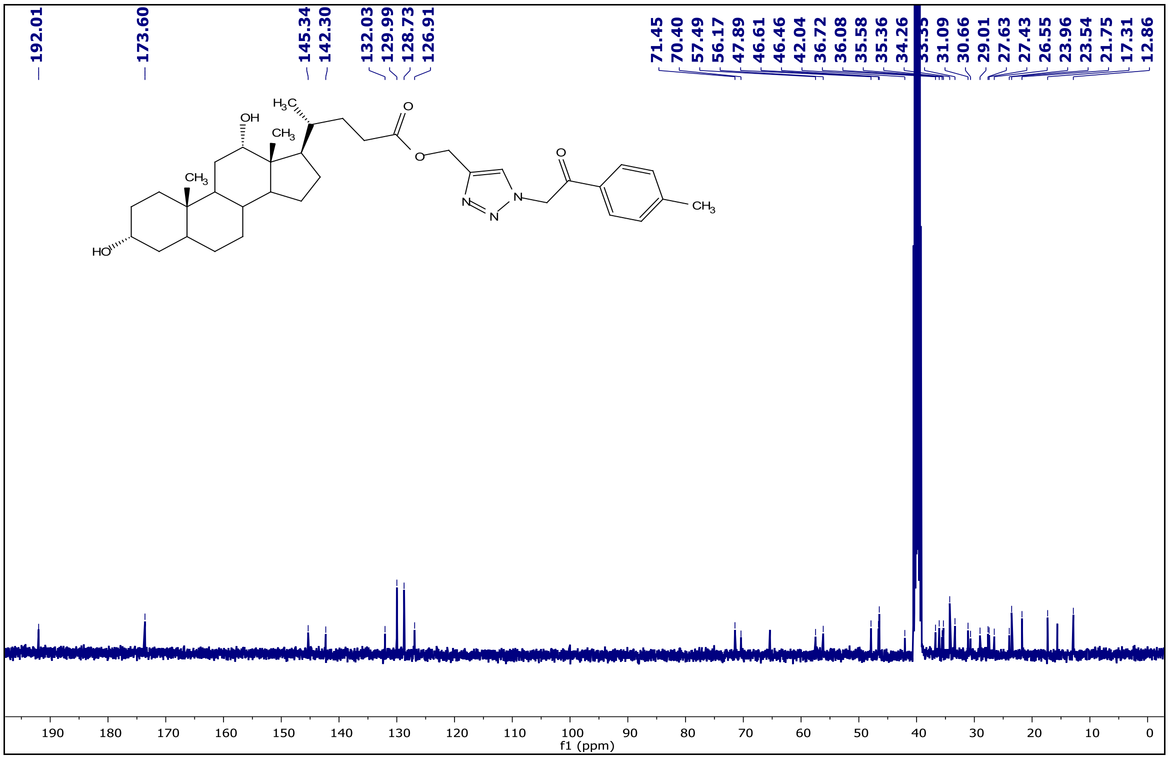

^{1}H NMR of 6cb

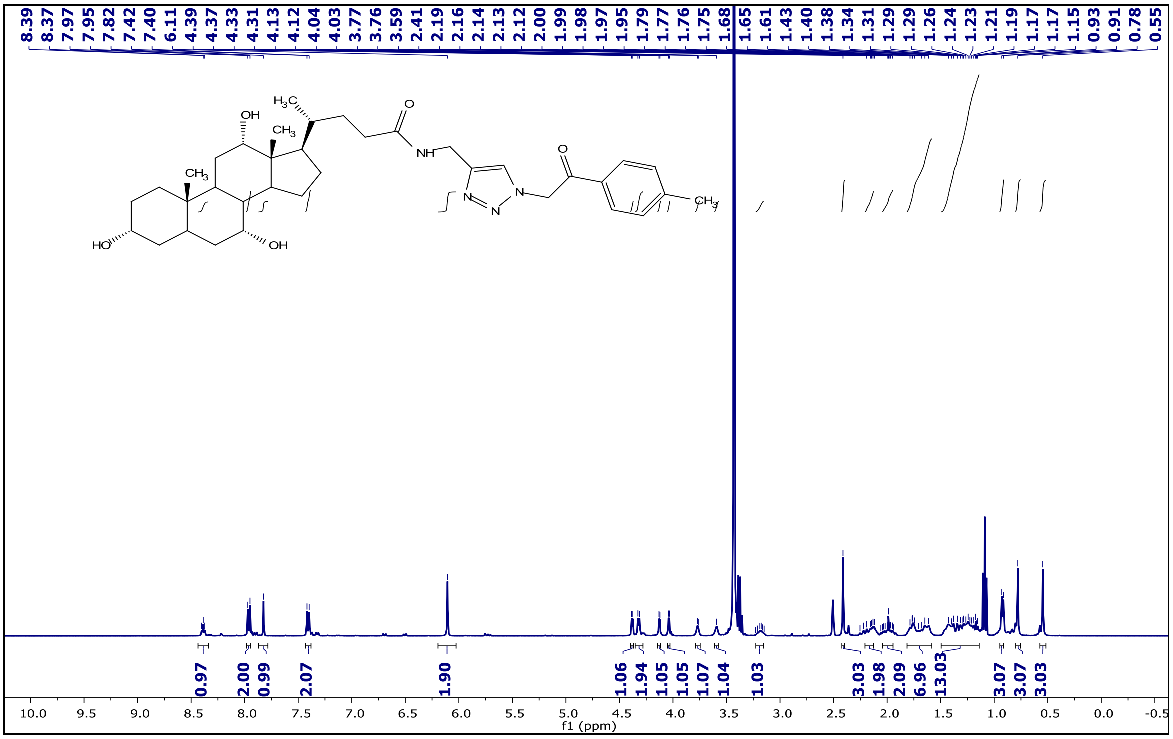

^{13}C NMR of 6cb

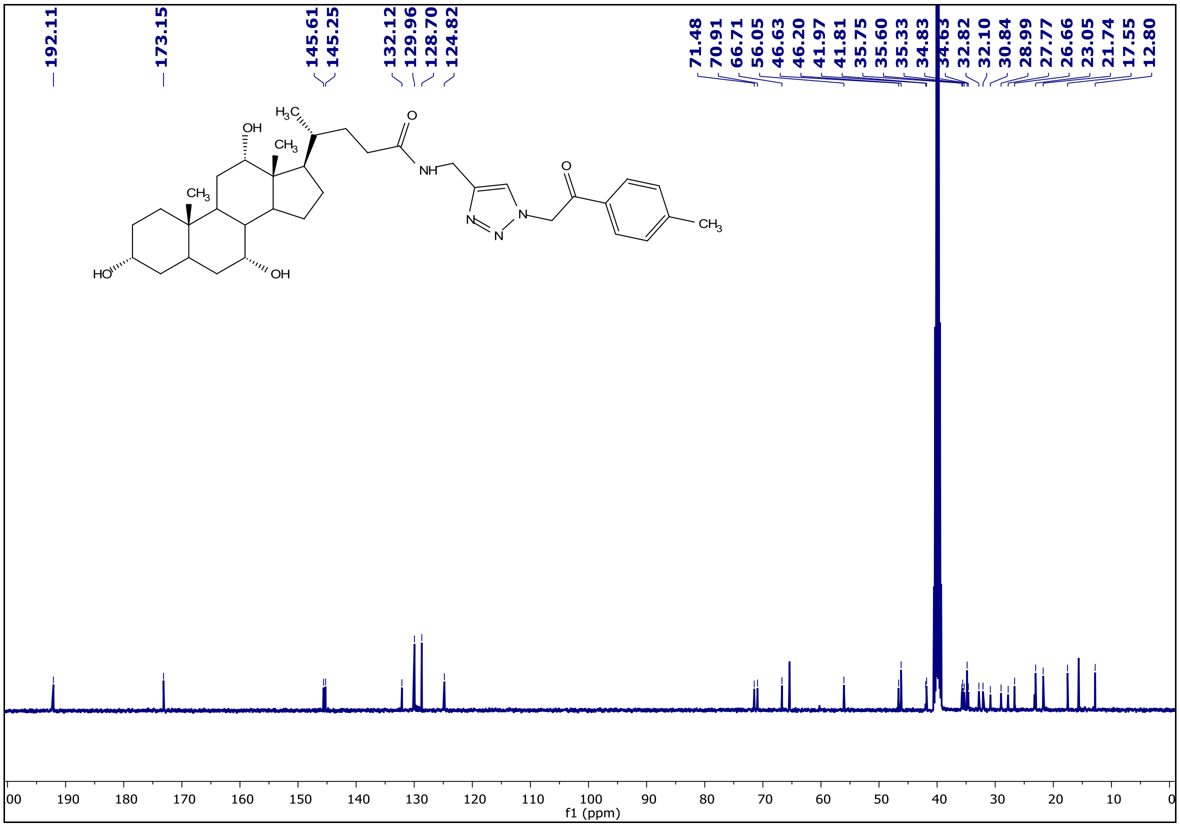

^{1}H NMR of 6db

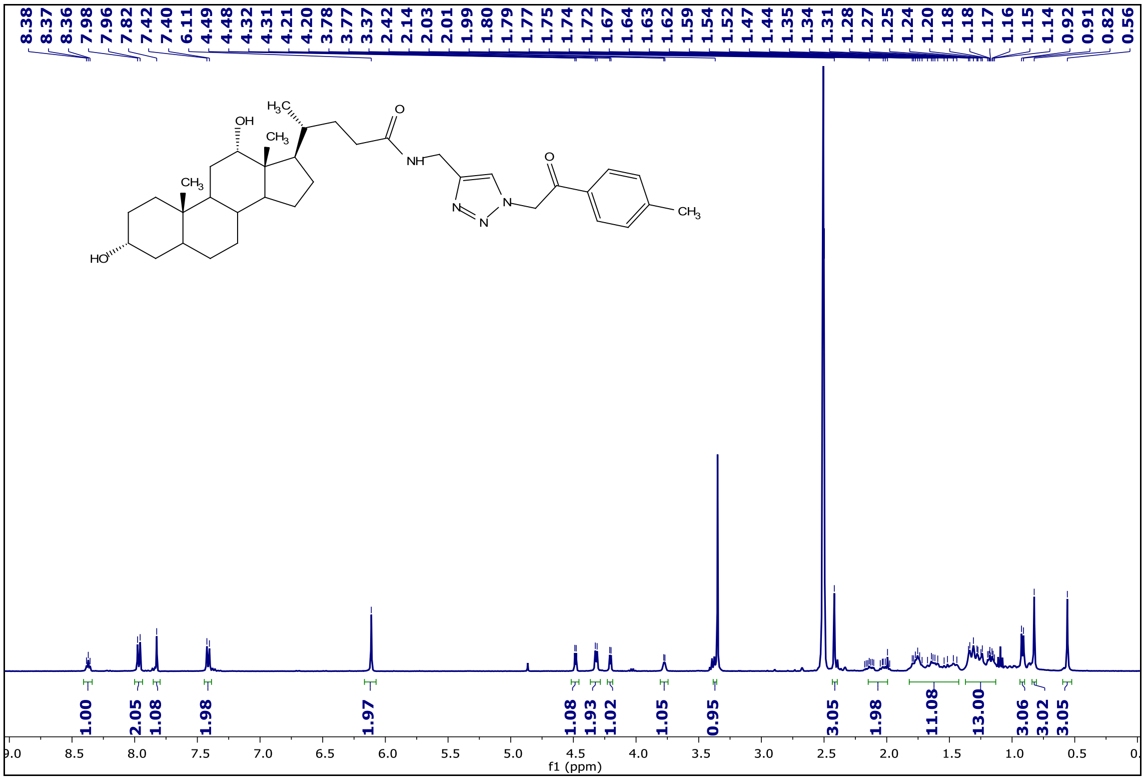

^{13}C NMR of 6db

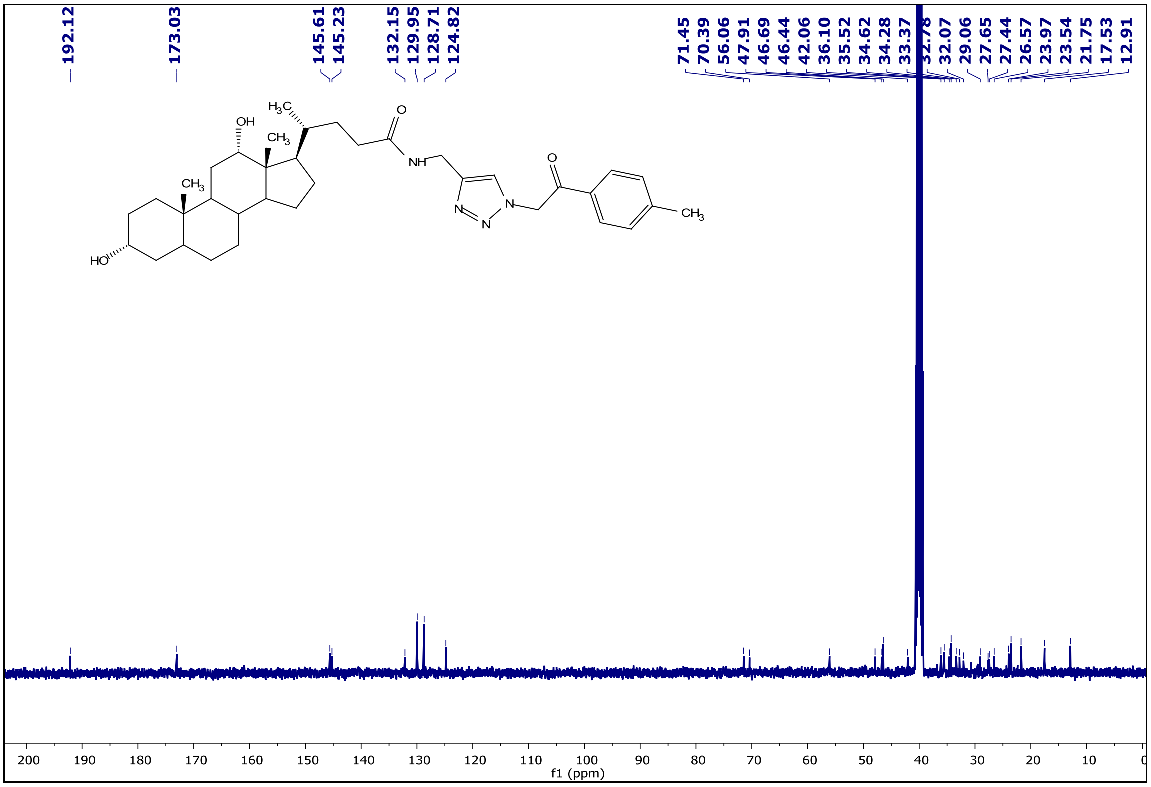

^{1}H NMR of 6ac

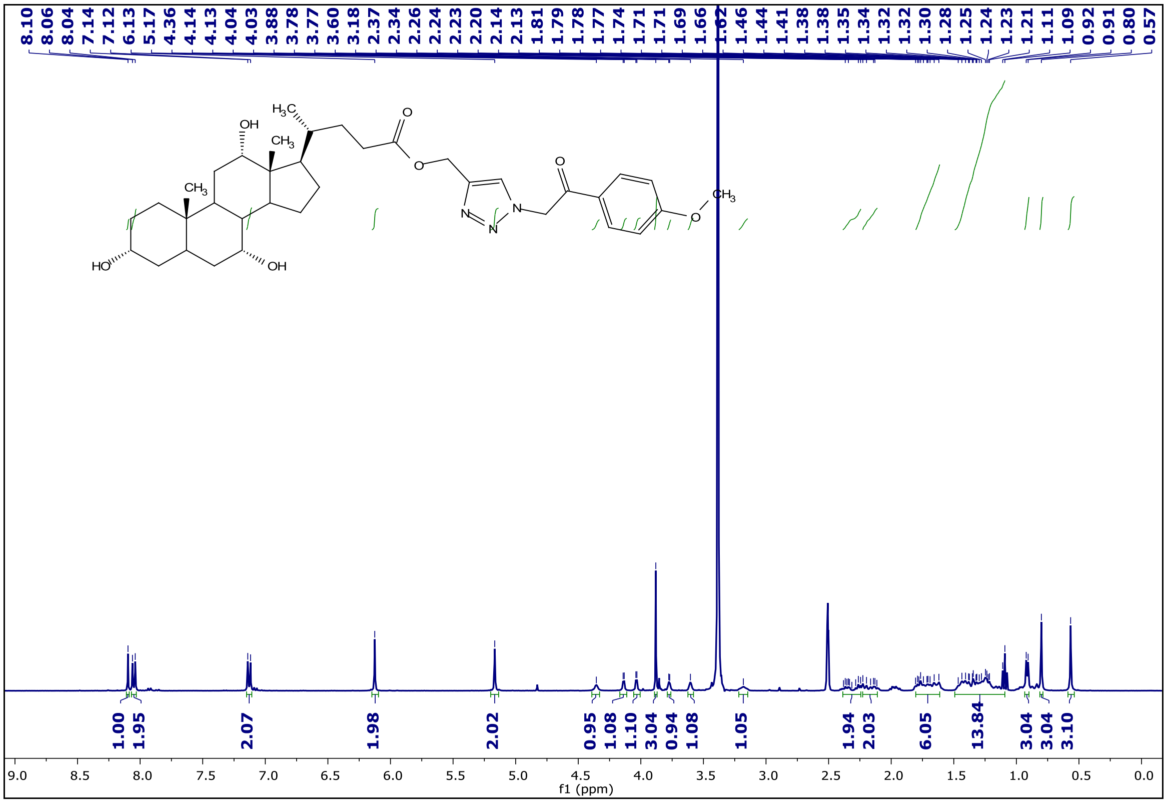

^{13}C NMR of 6ac

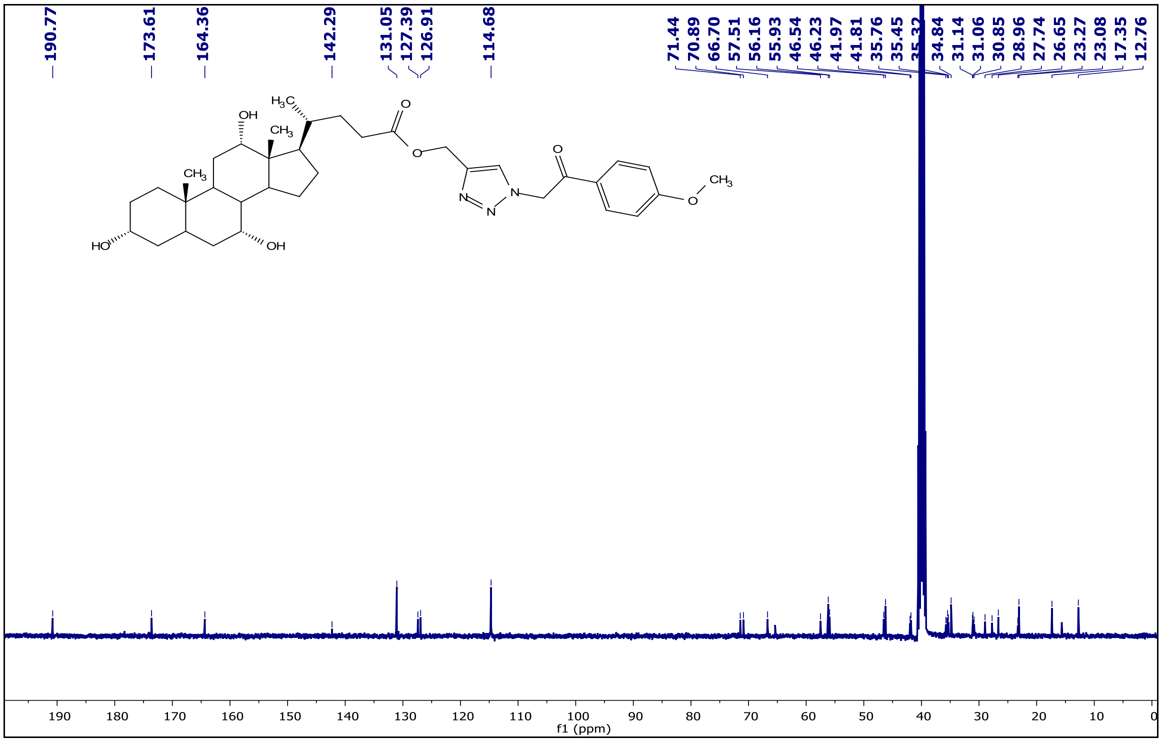

^{1}H NMR of 6bc

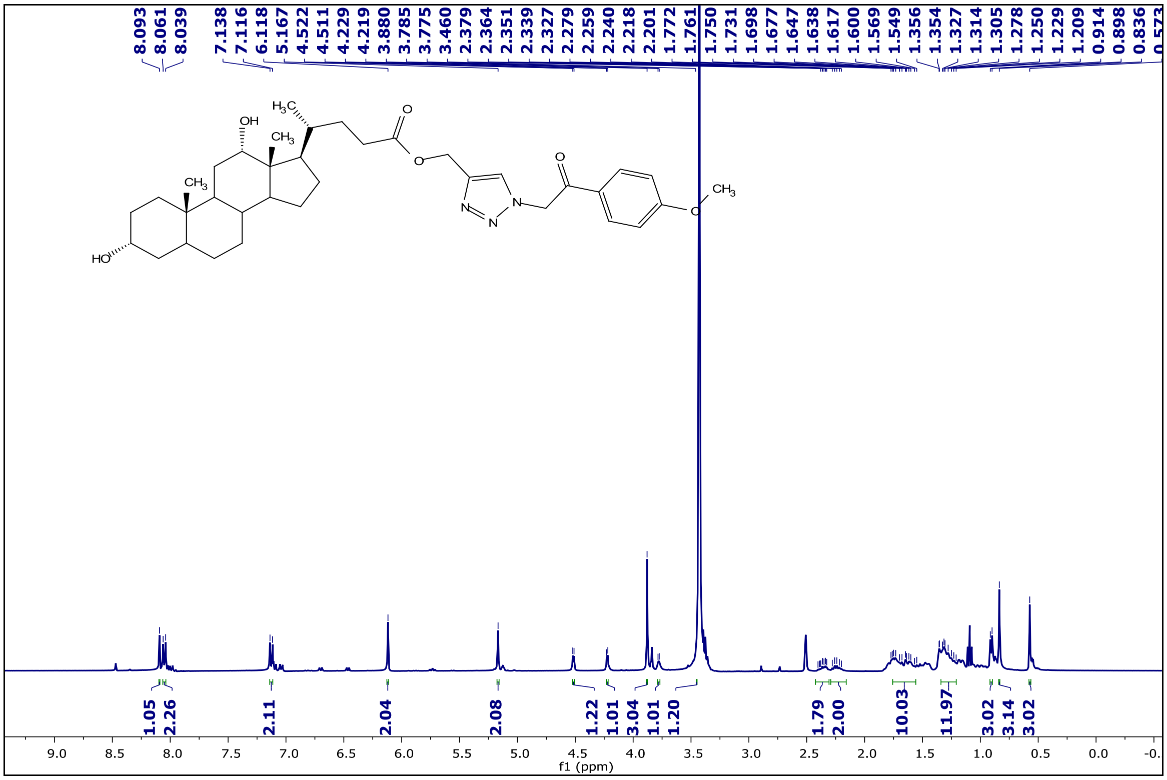

^{13}C NMR of 6bc

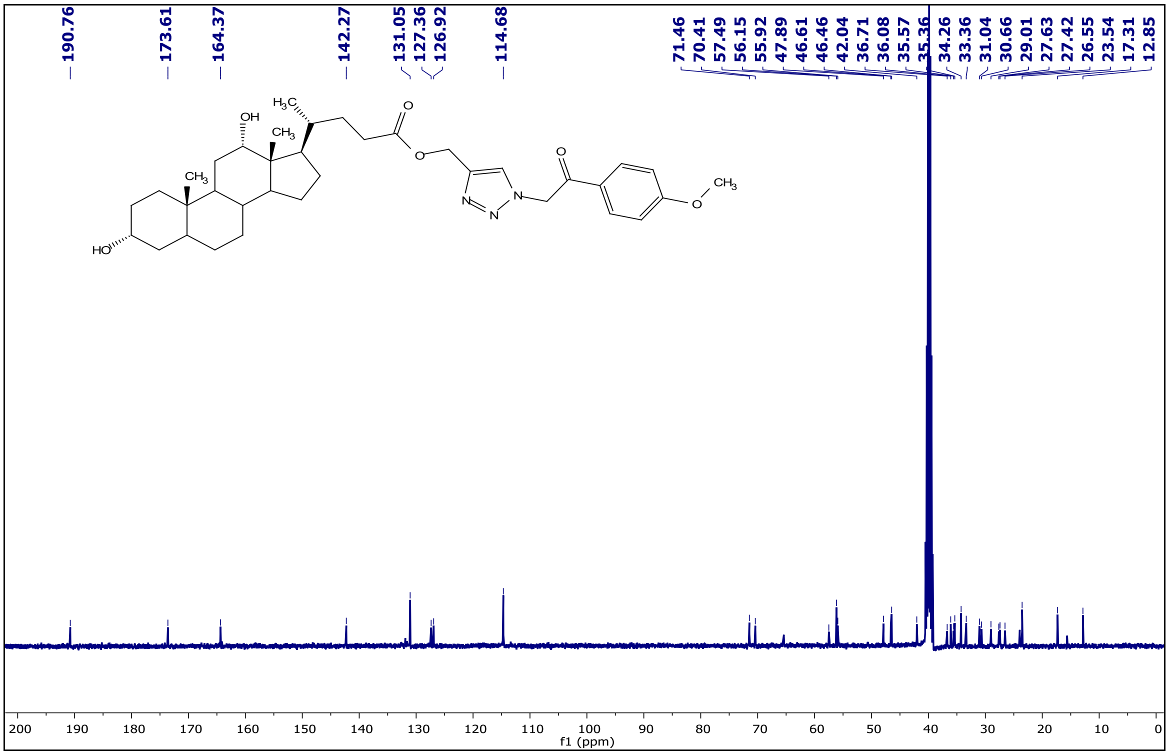

^{1}H NMR of 6cc

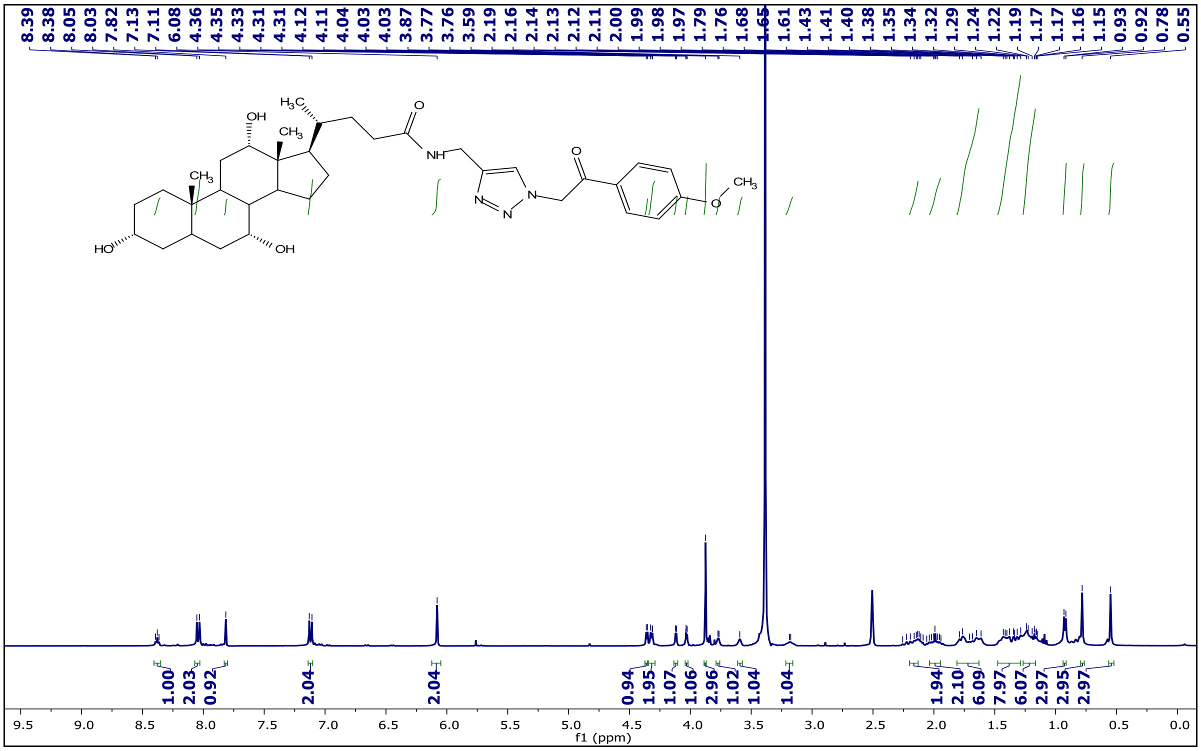

^{13}C NMR of 6cc

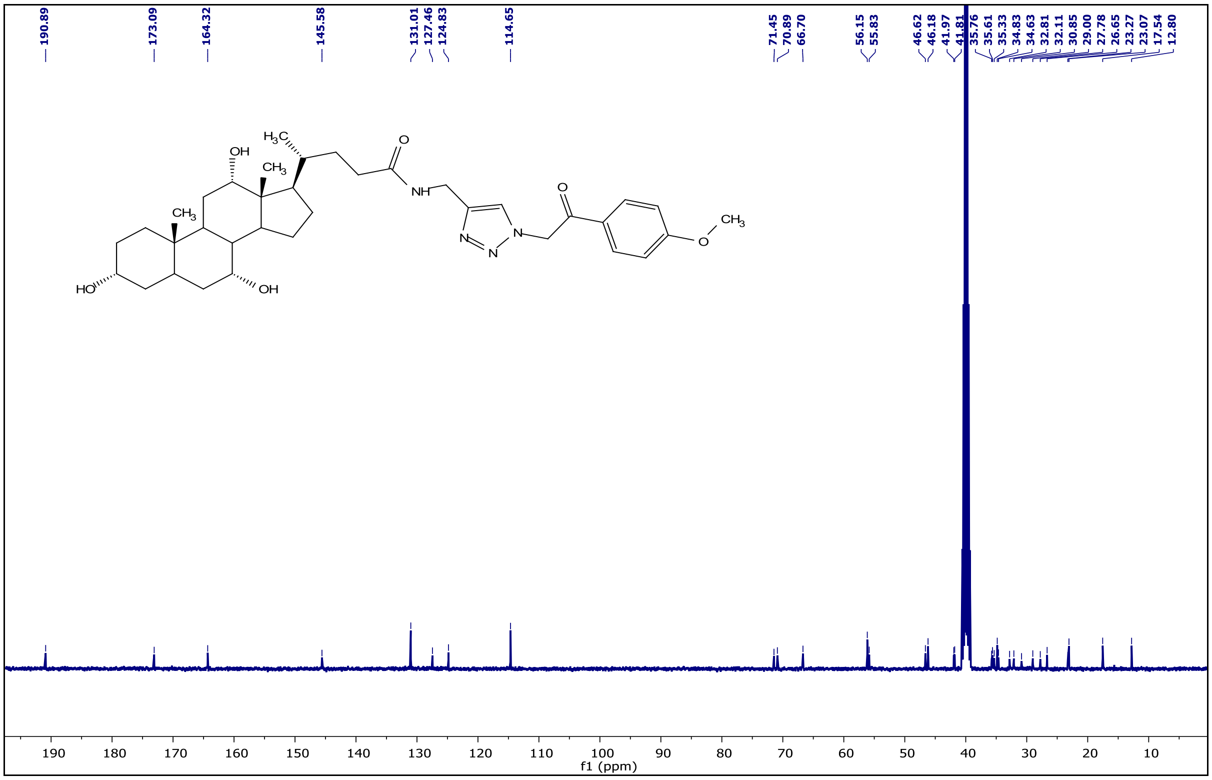

^{1}H NMR of 6dc

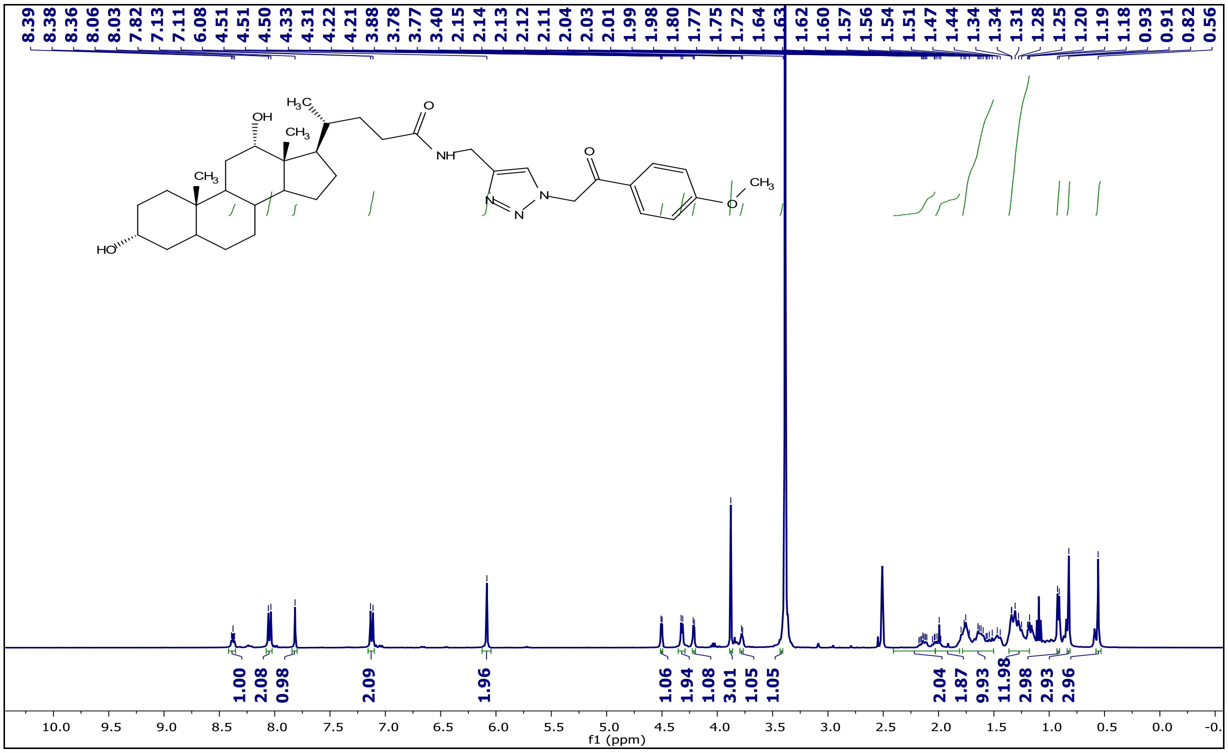

^{13}C NMR of 6dc

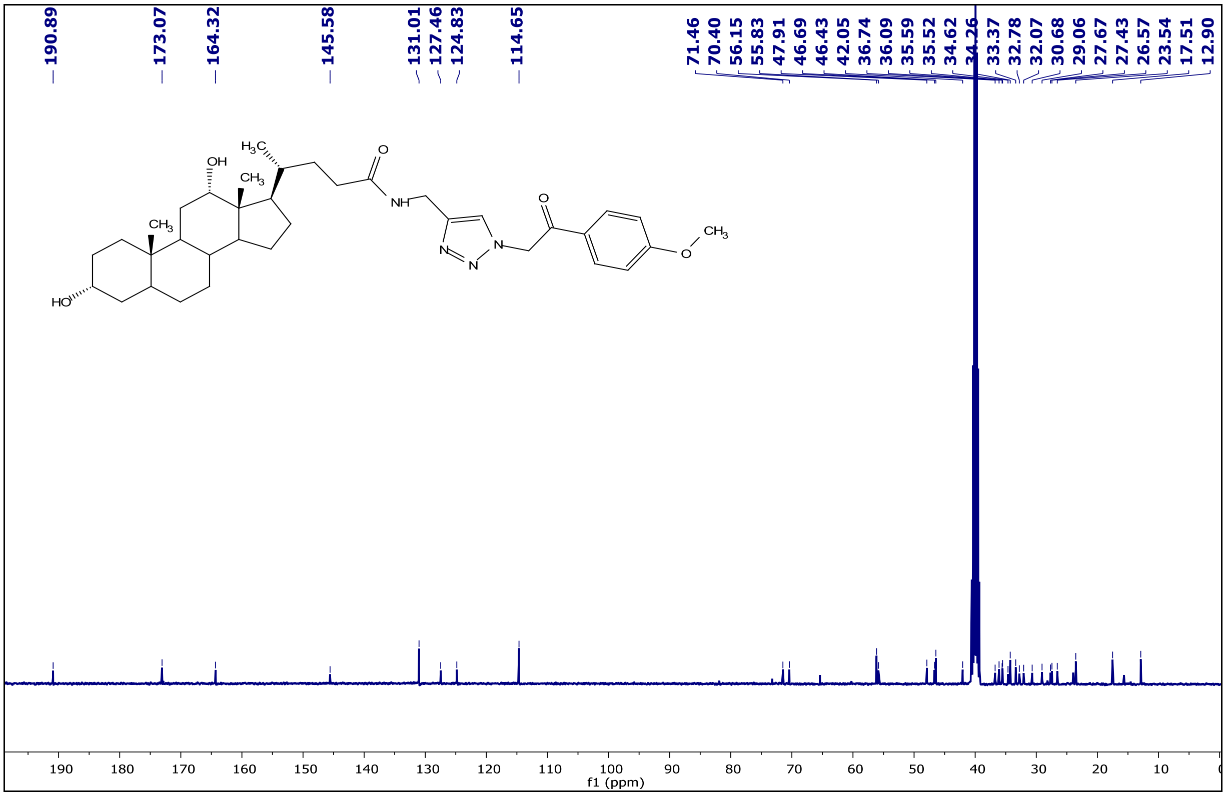

^{1}H NMR of 6ad

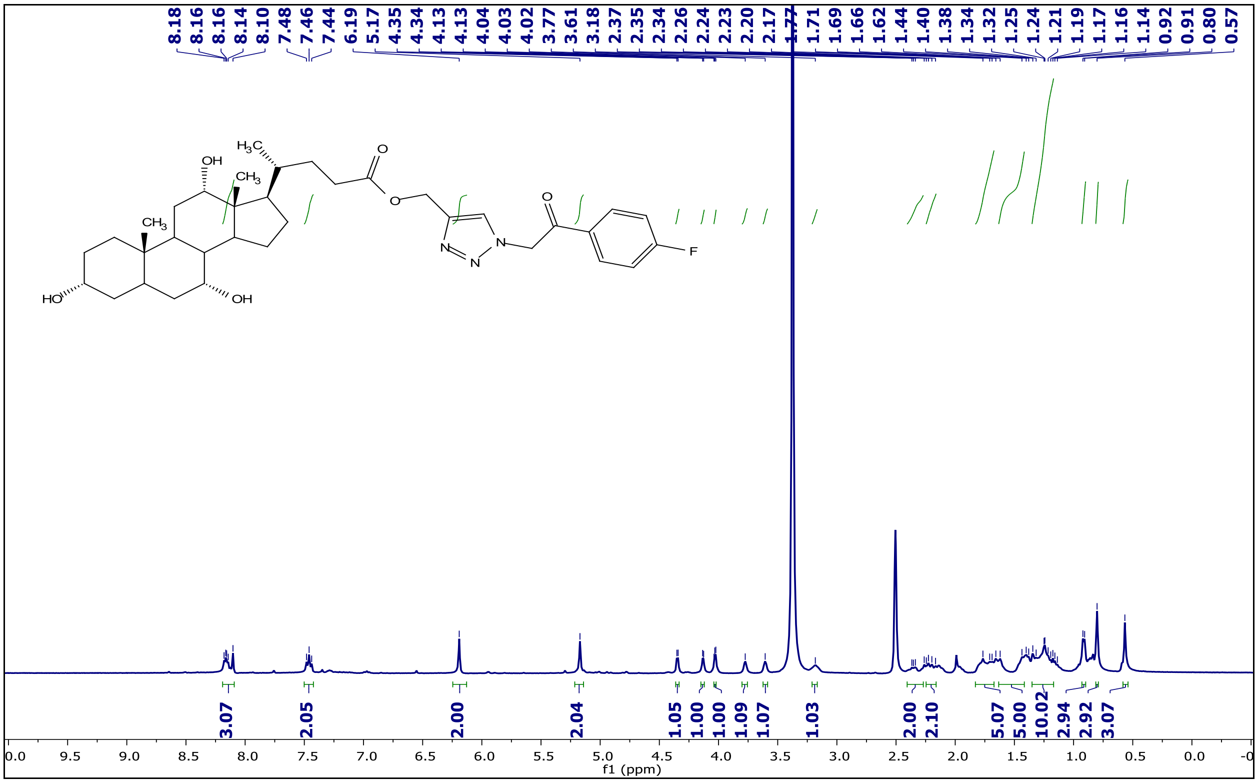

^{13}C NMR of 6ad

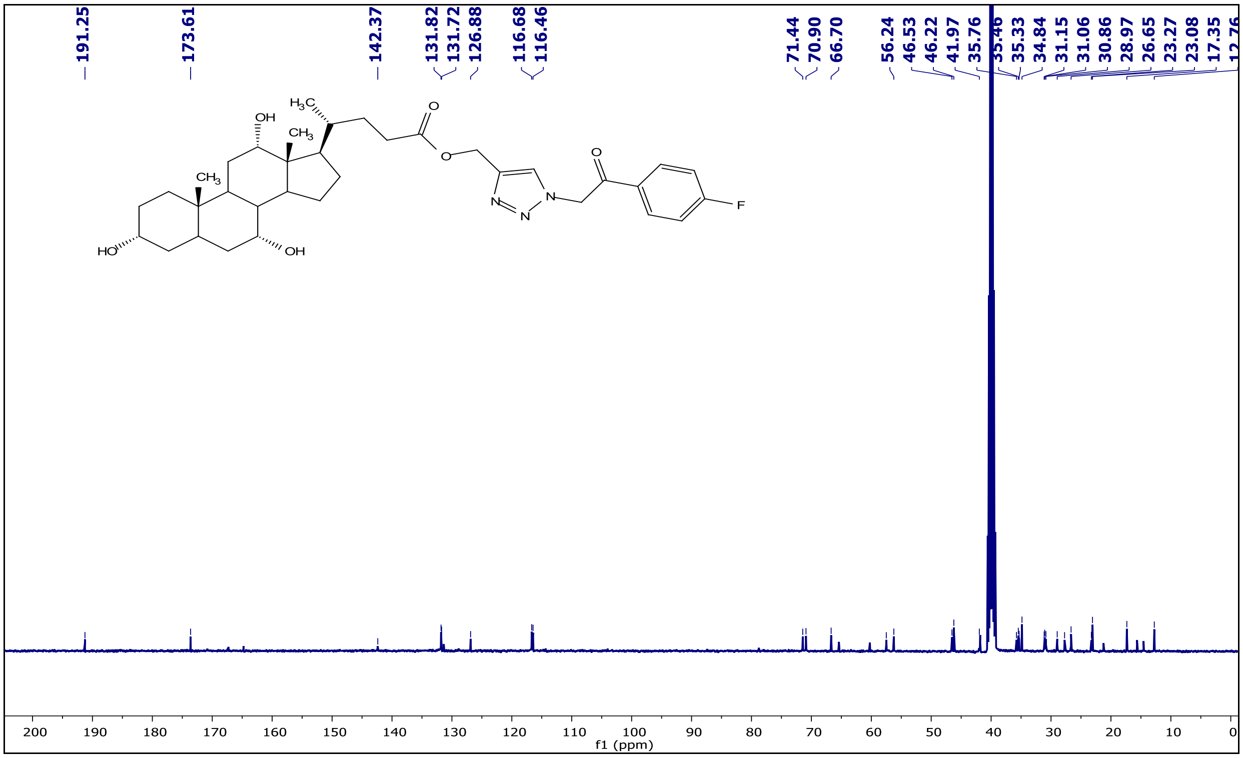

^{1}H NMR of 6bd

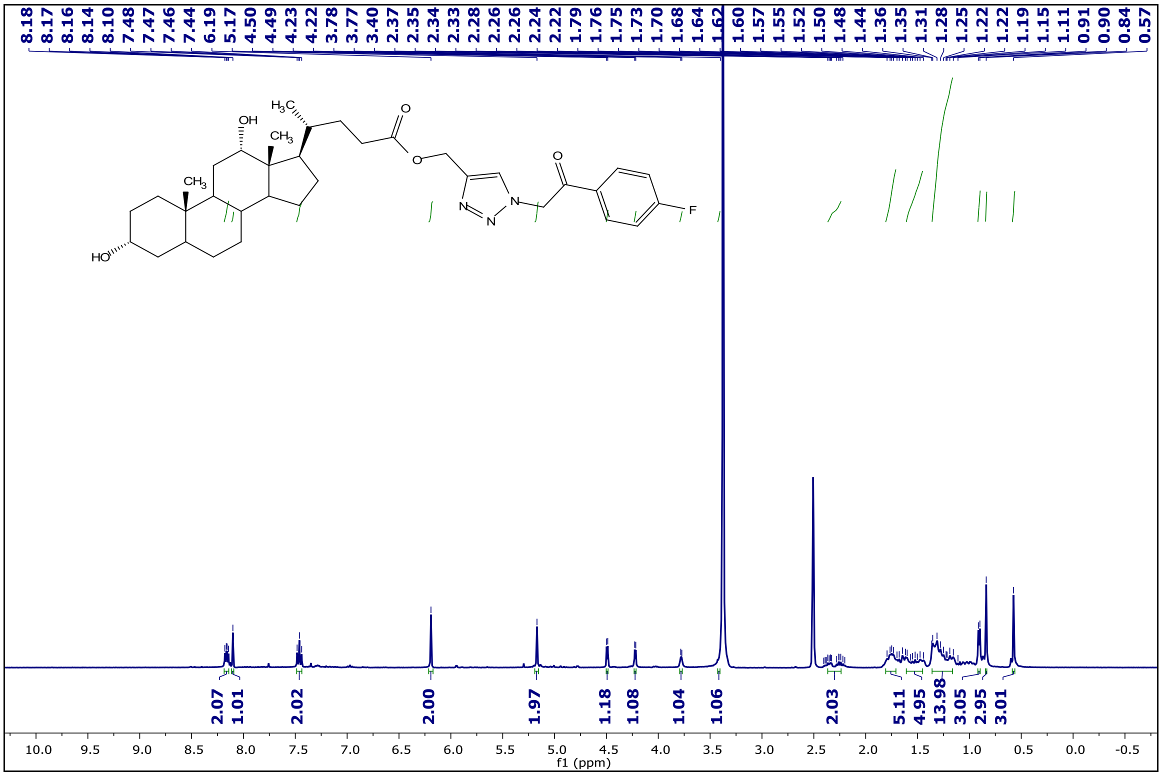

^{13}C NMR of 6bd

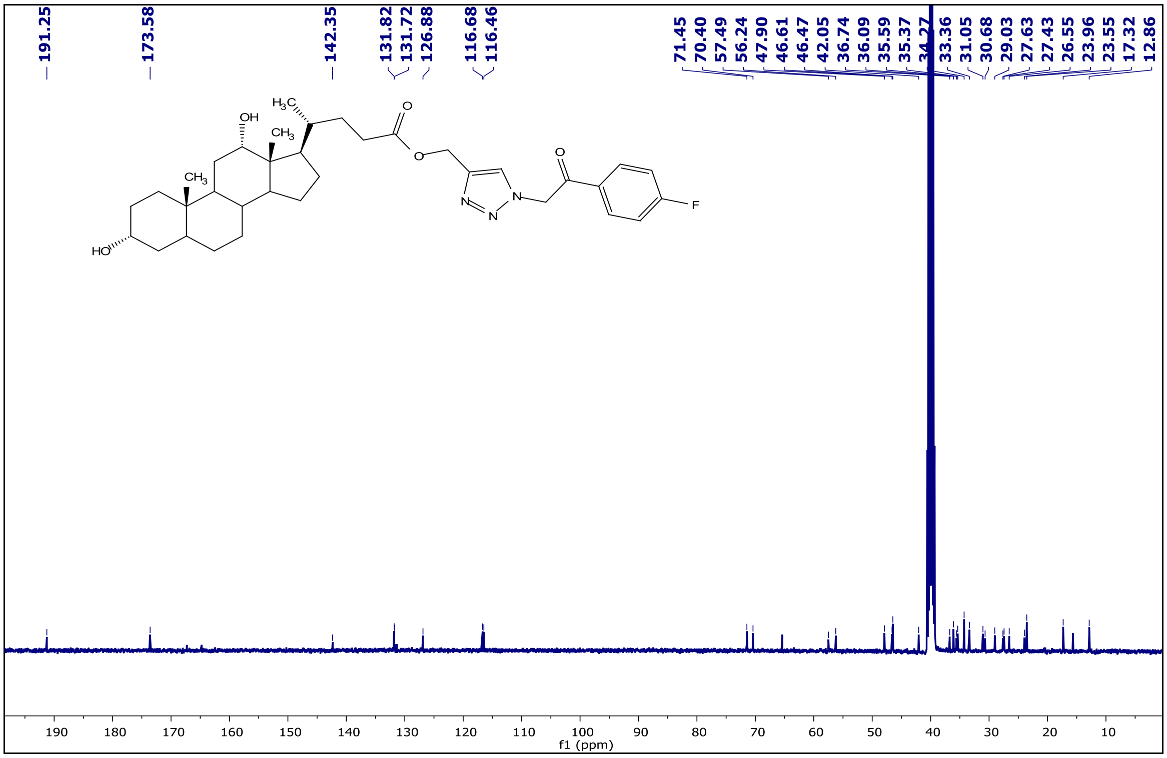

^{1}H NMR of 6cd

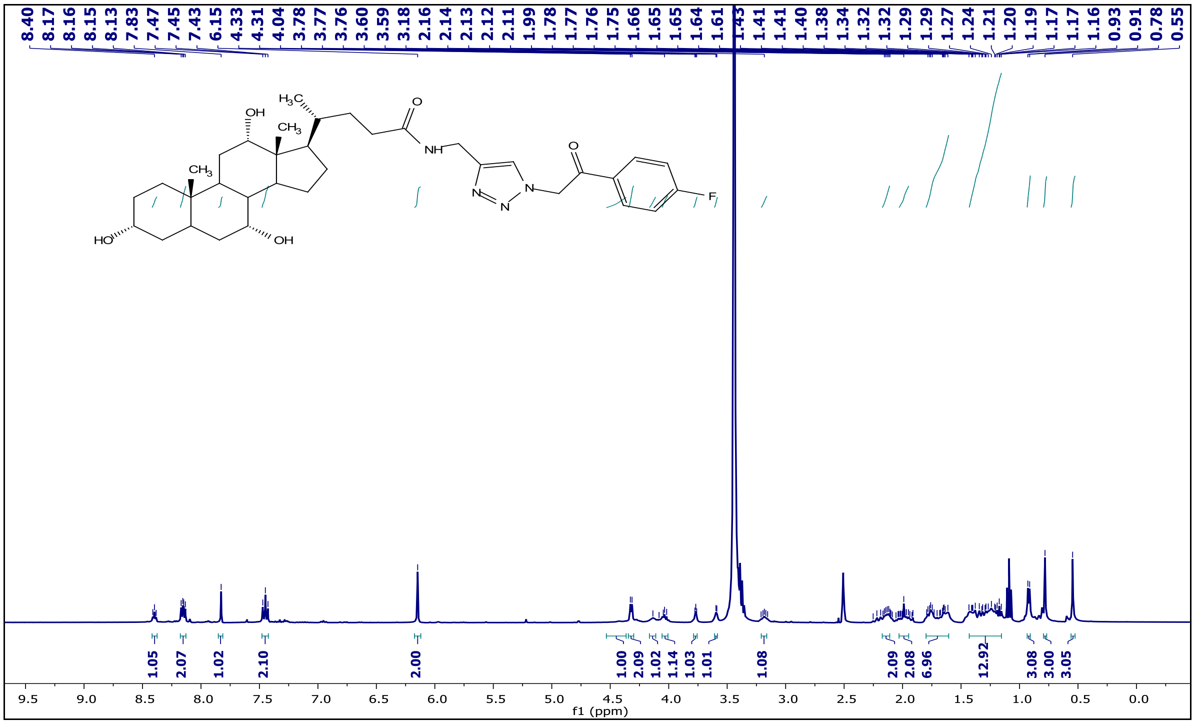

^{13}C NMR of 6cd

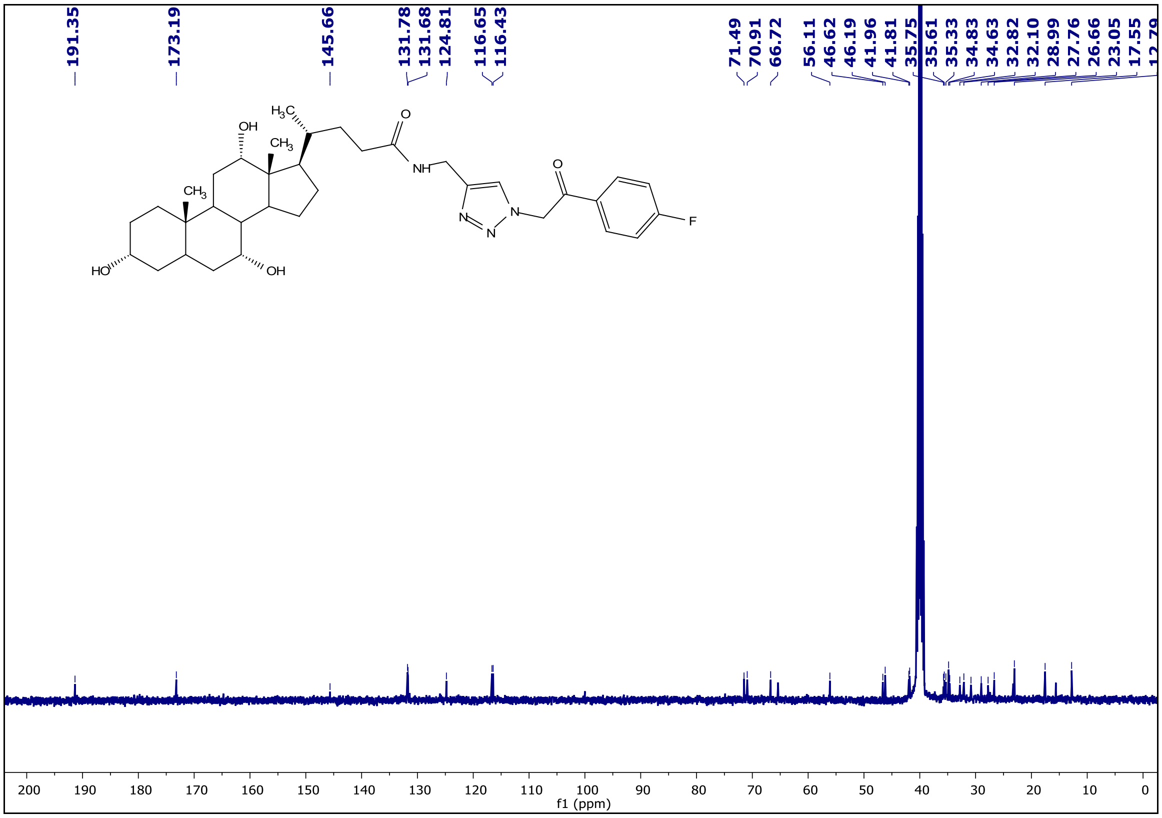

^{1}H NMR of 6dd

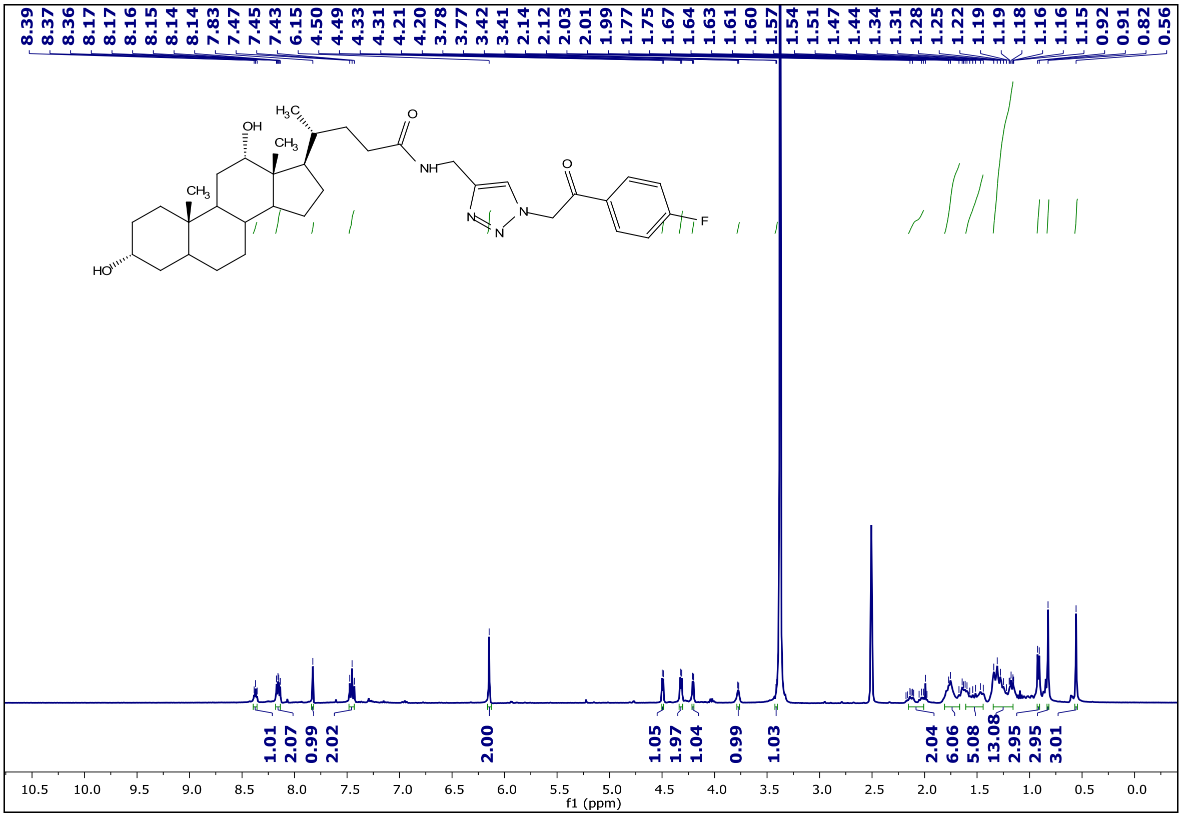

^{13}C NMR of 6dd

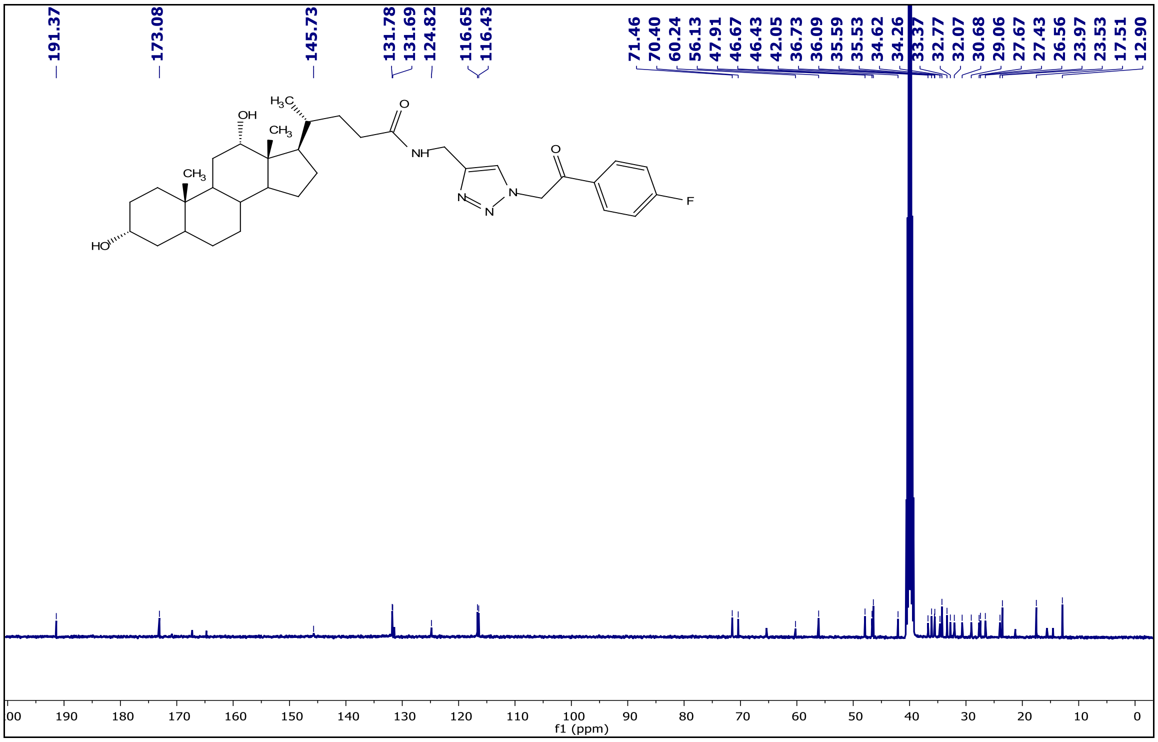

^{1}H NMR of 6ae

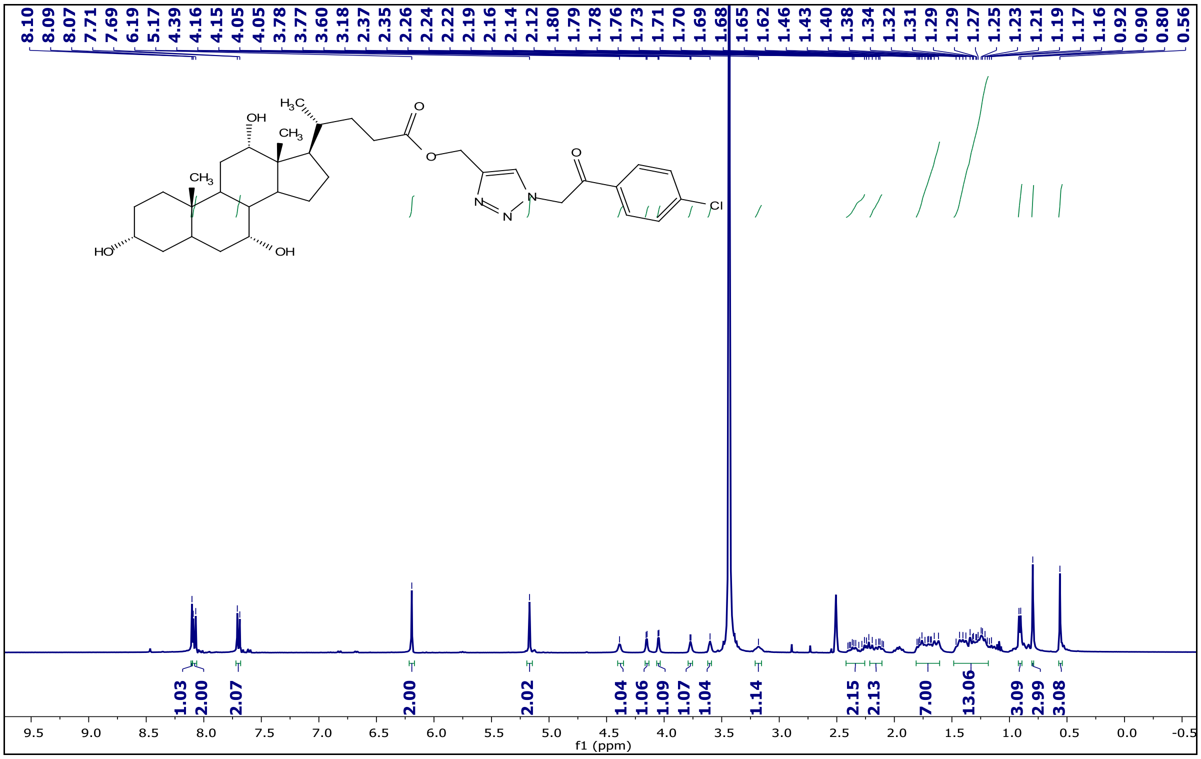

^{13}C NMR of 6ae

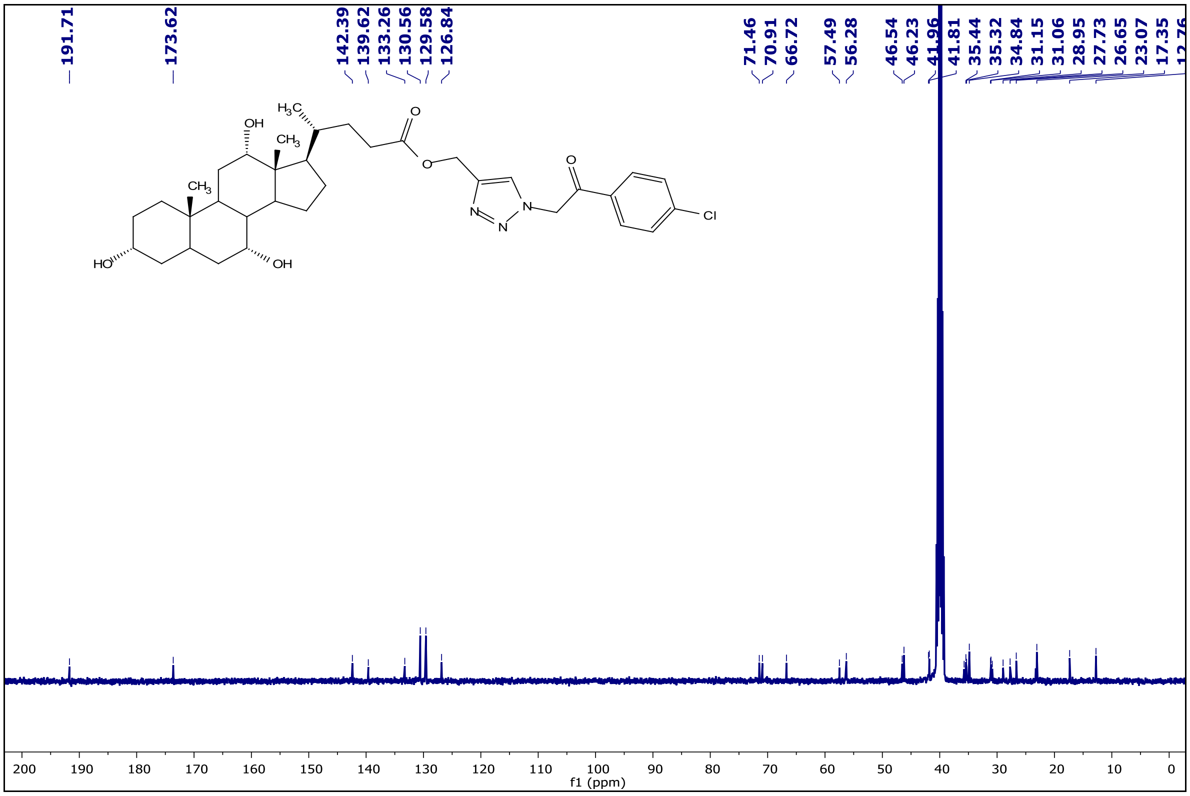

^{1}H NMR of 6be

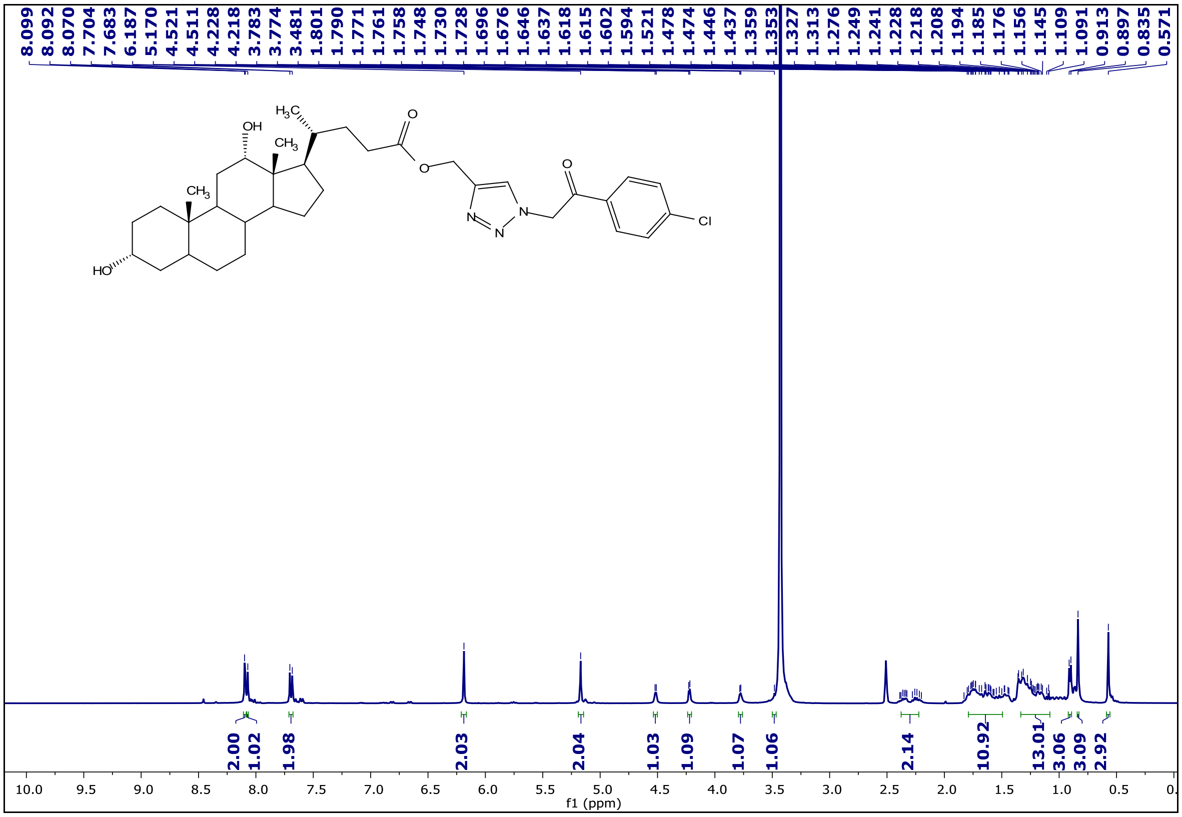

^{13}C NMR of 6be

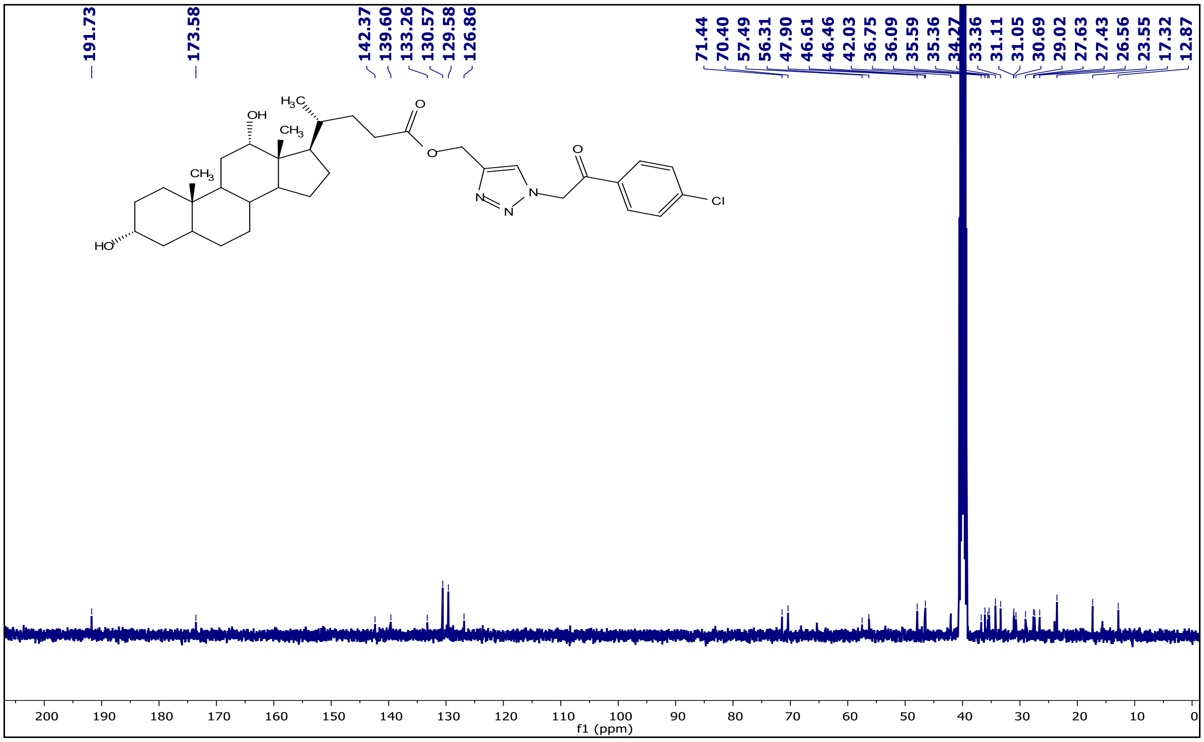

^{1}H NMR of 6ce

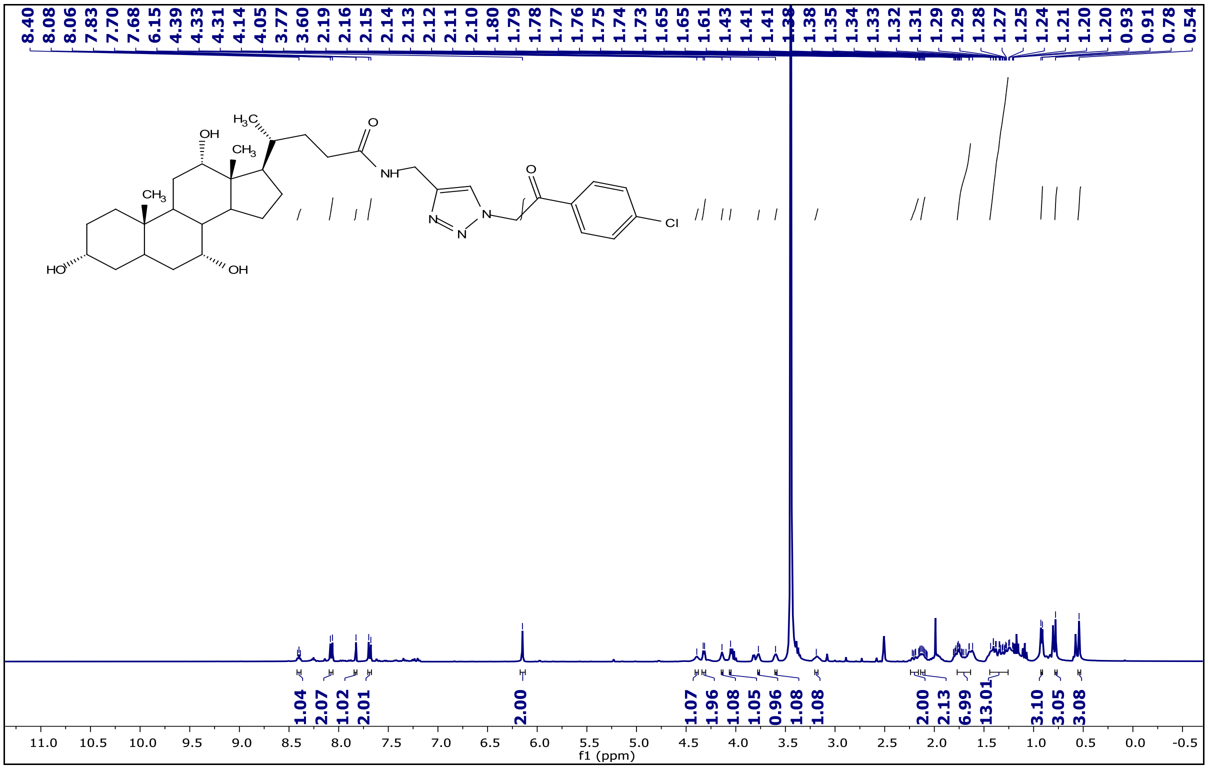

^{13}C NMR of 6ce

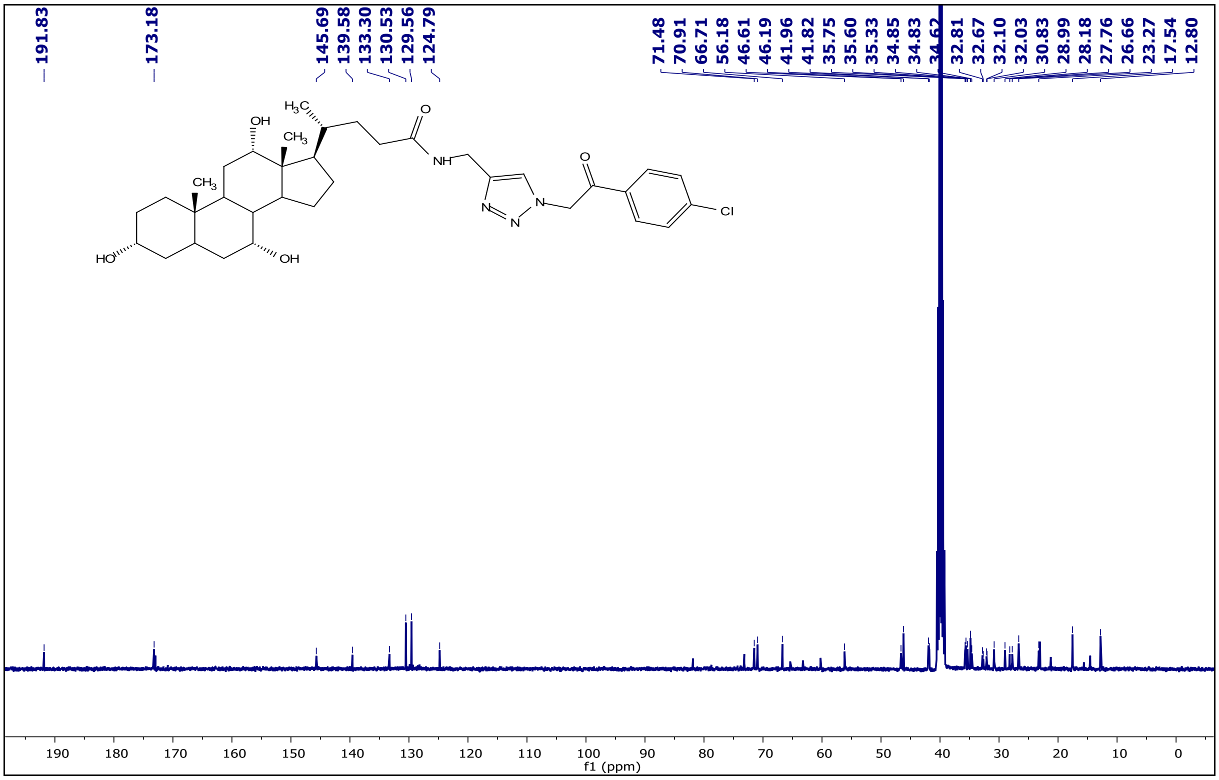

^{1}H NMR of 6de

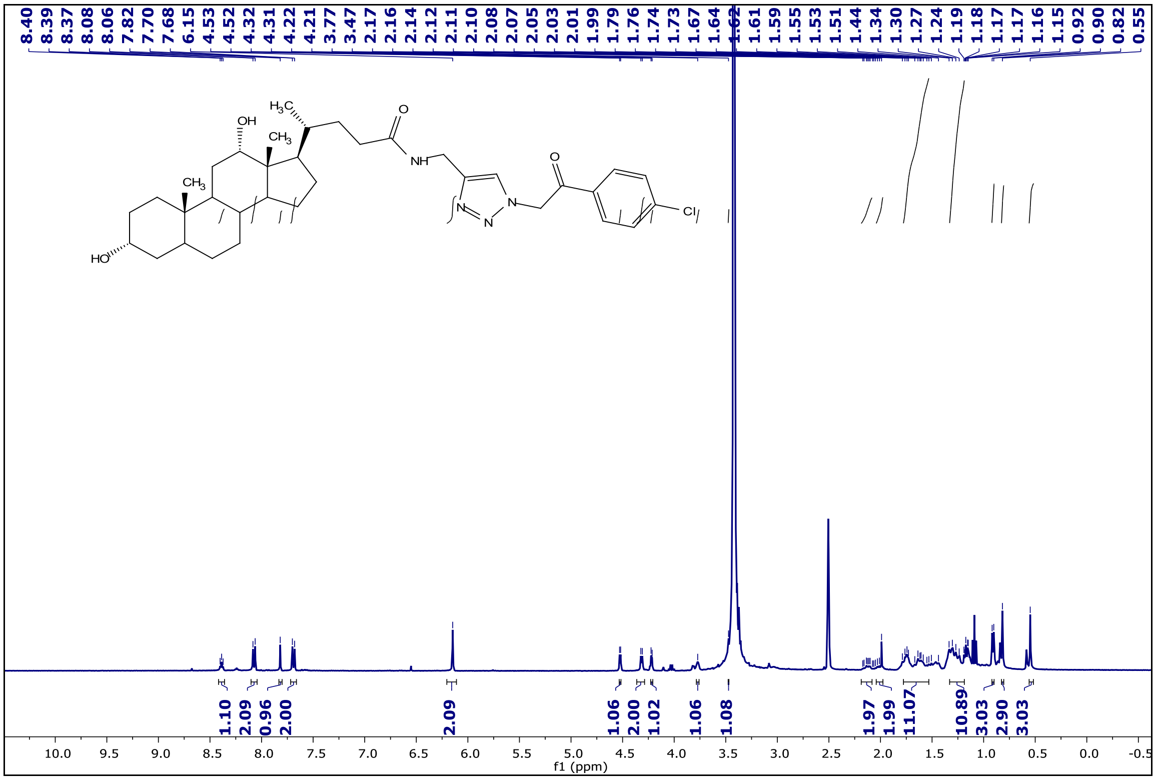

^{13}C NMR of 6de

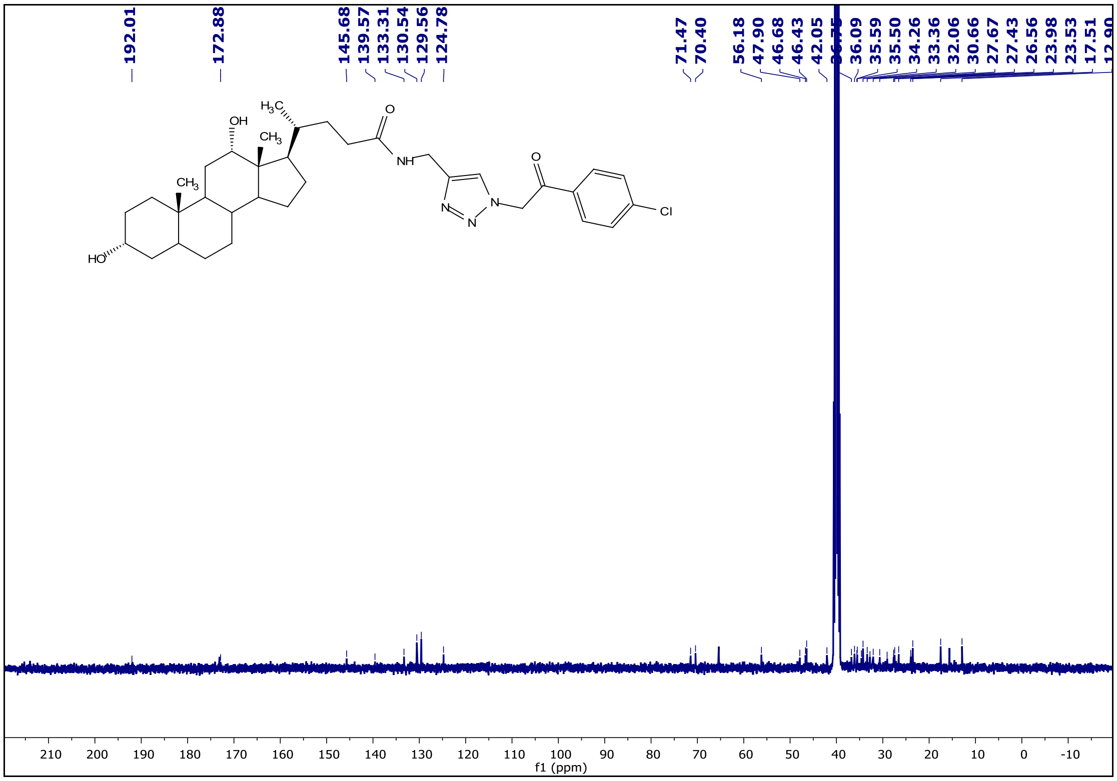

^{1}H NMR of 6af

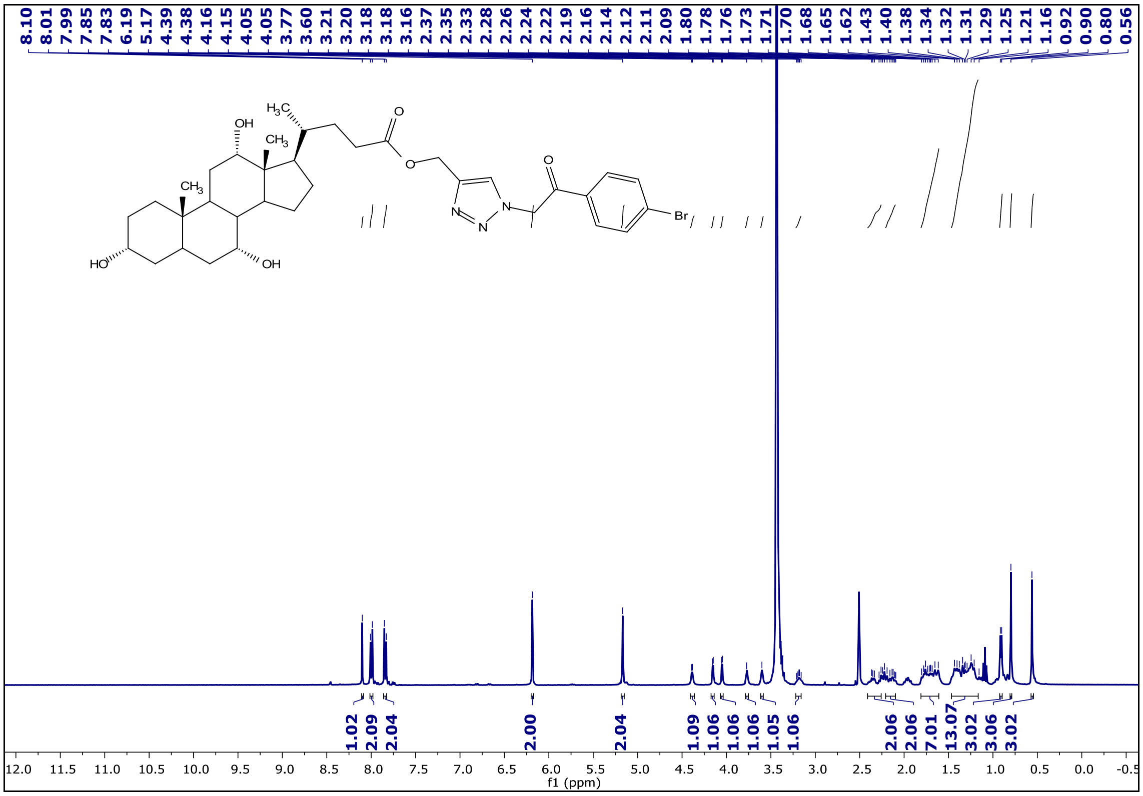

^{13}C NMR of 6af

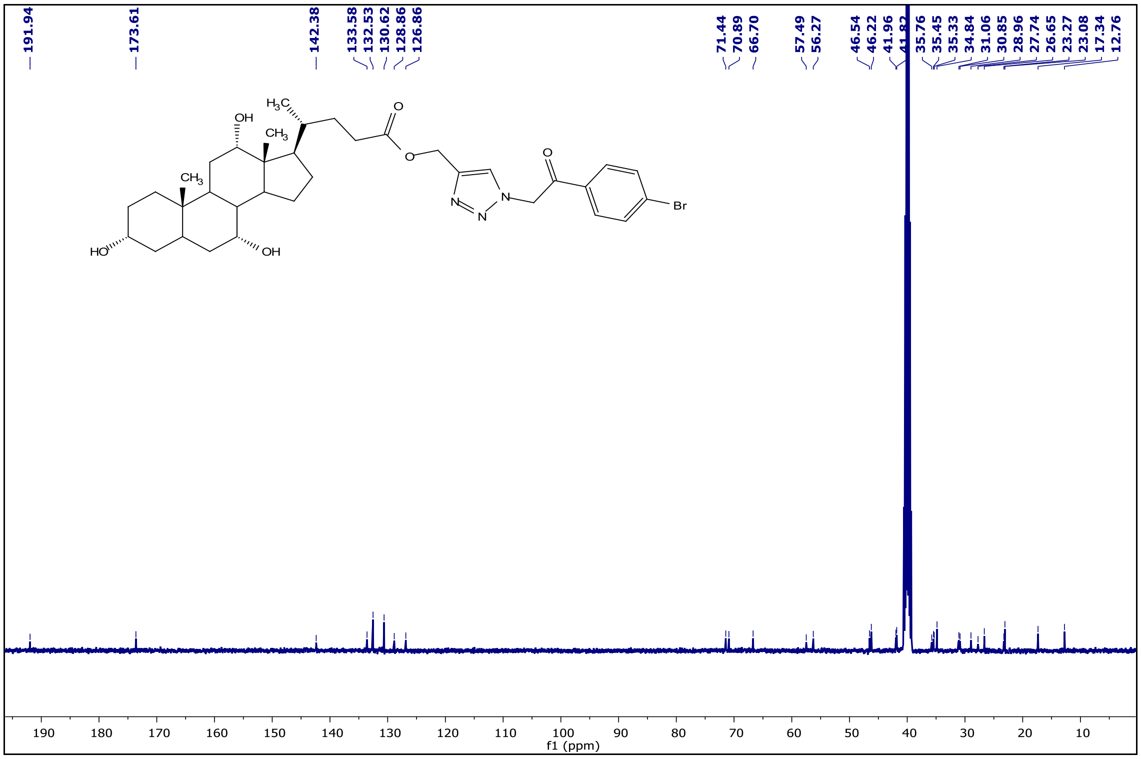

^{1}H NMR of 6bf

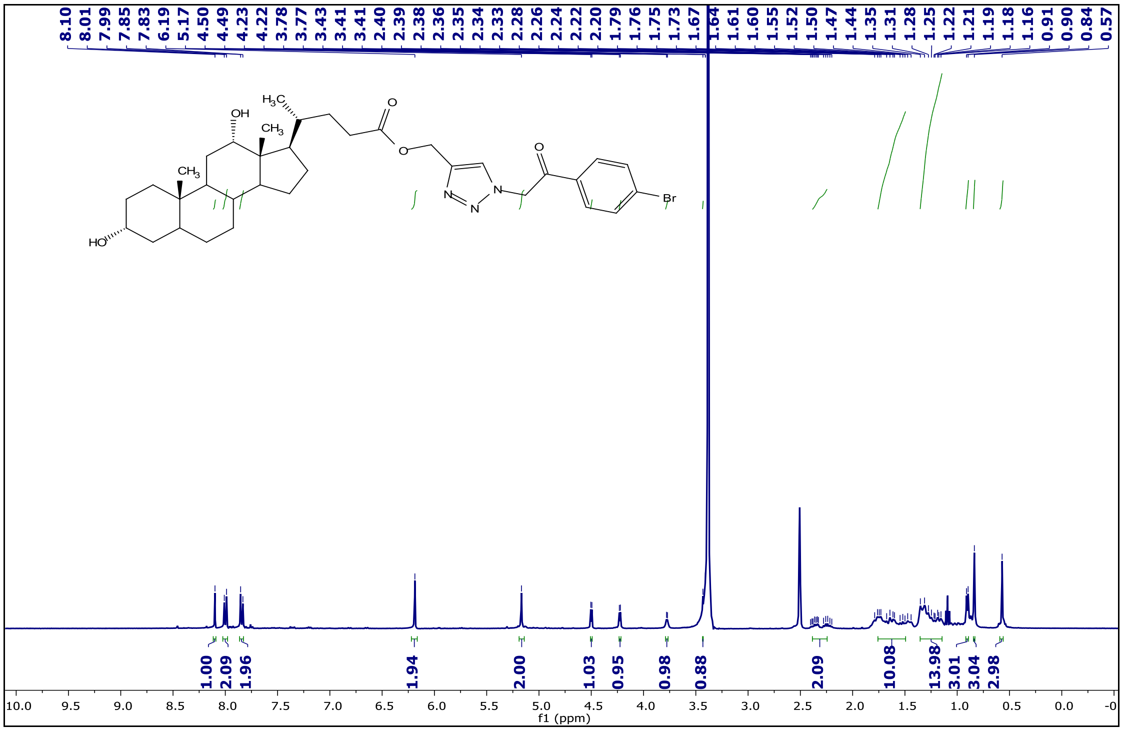

^{13}C NMR of 6bf

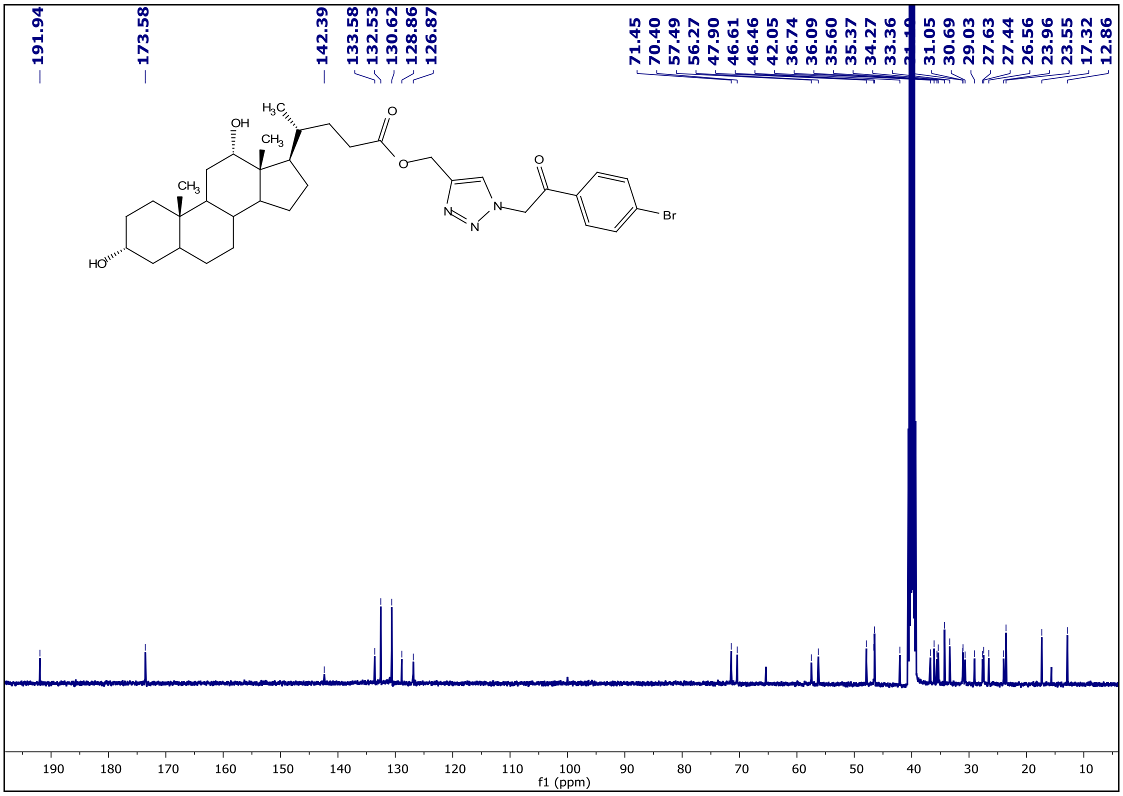

^{1}H NMR of 6cf

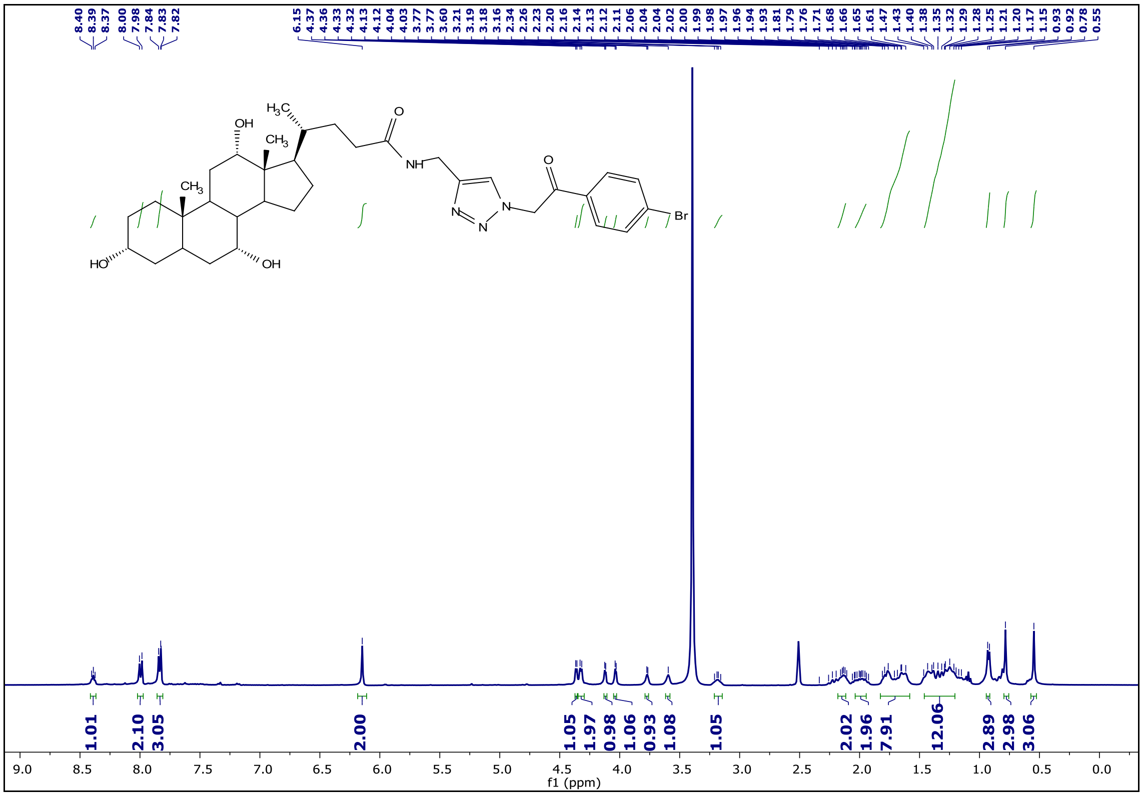

^{13}C NMR of 6cf

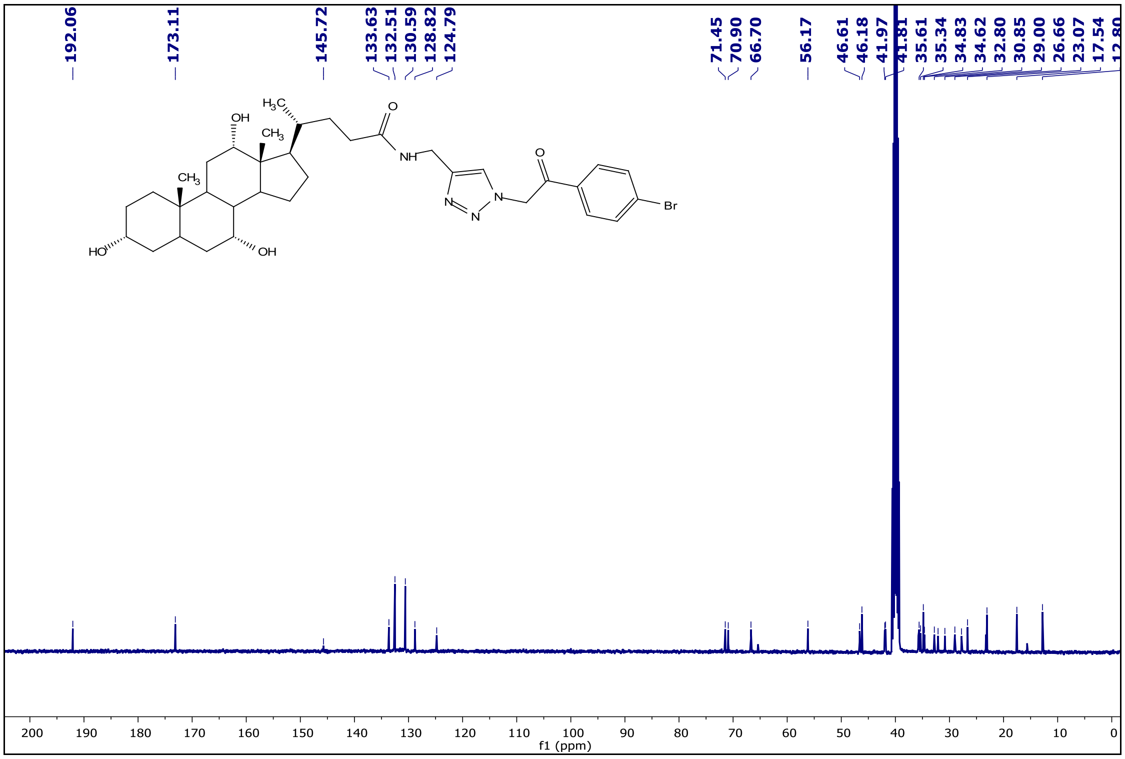

^{1}H NMR of 6df

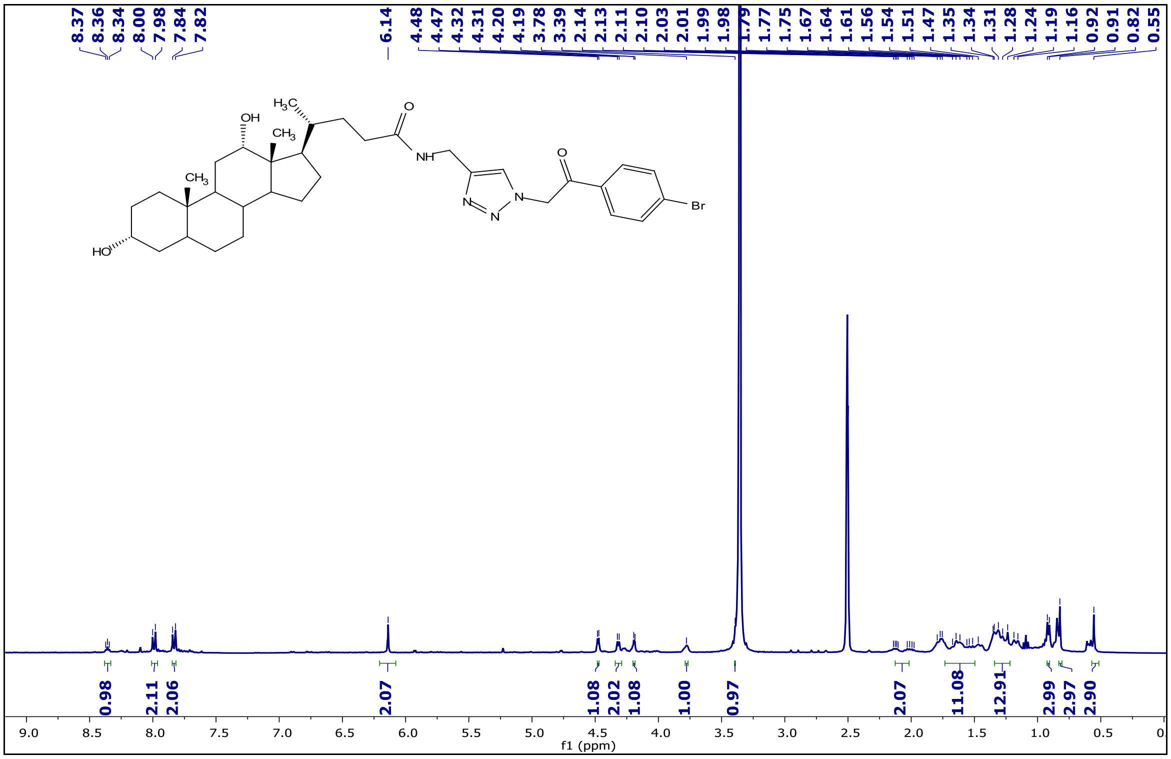

^{13}C NMR of 6df

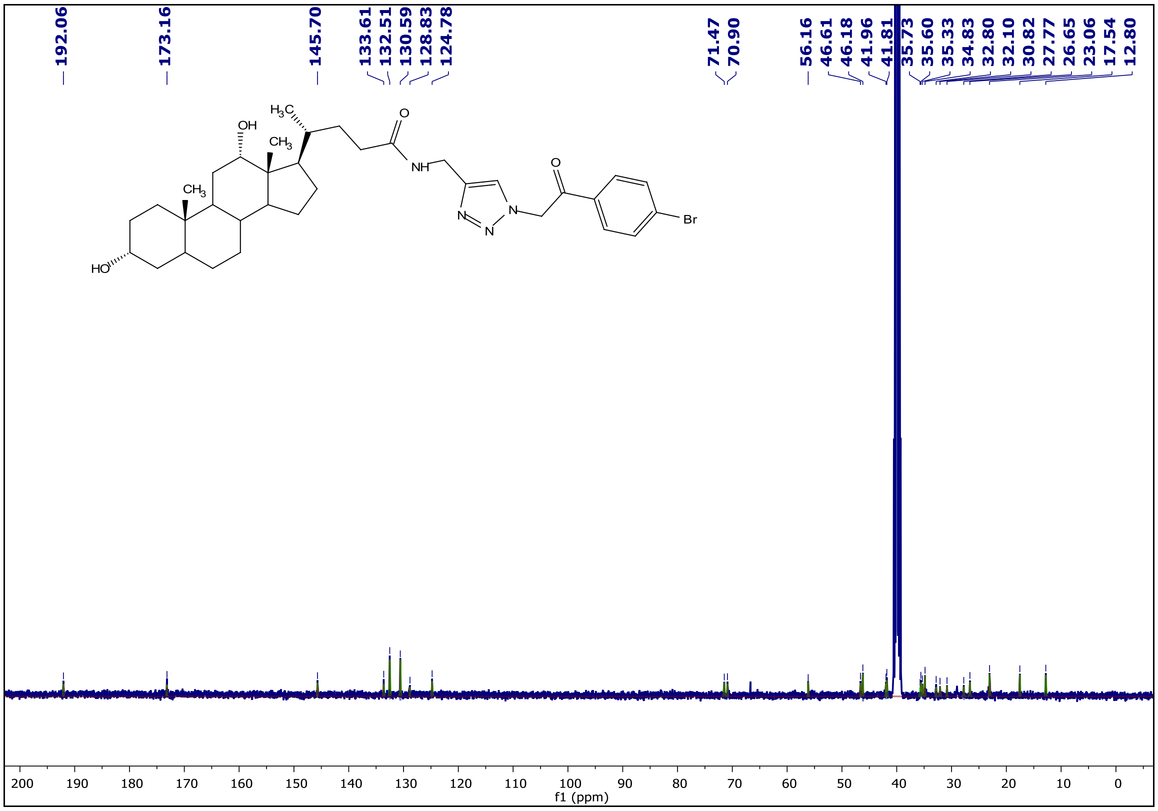

COSY of 6aa

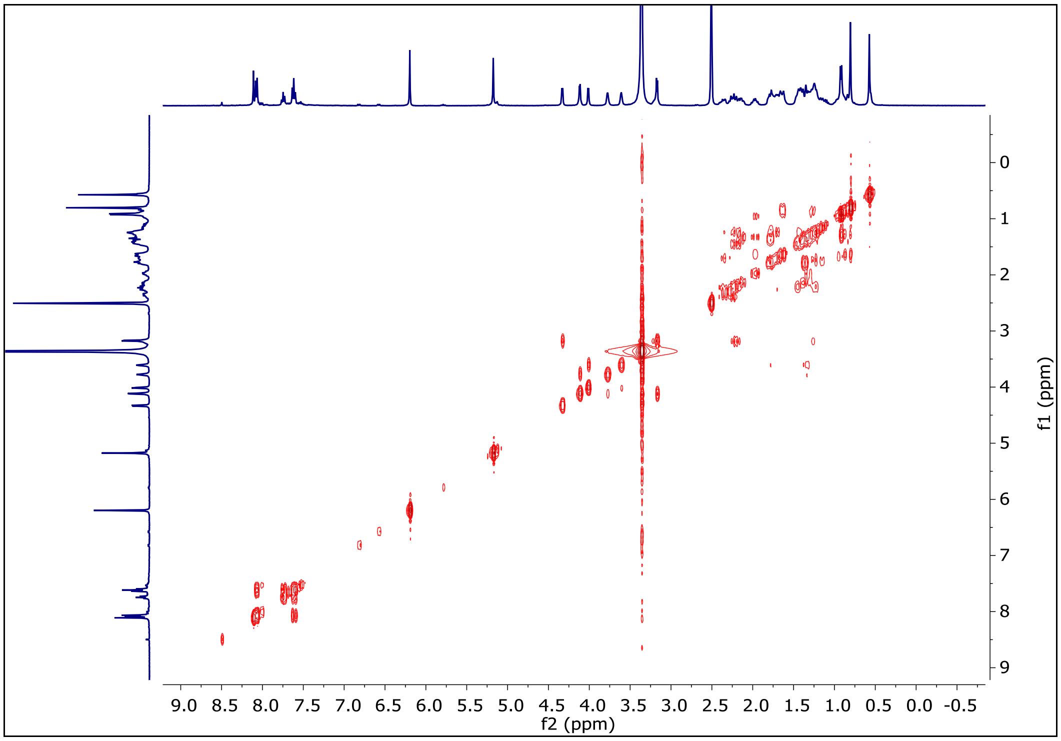

HSQC of 6aa

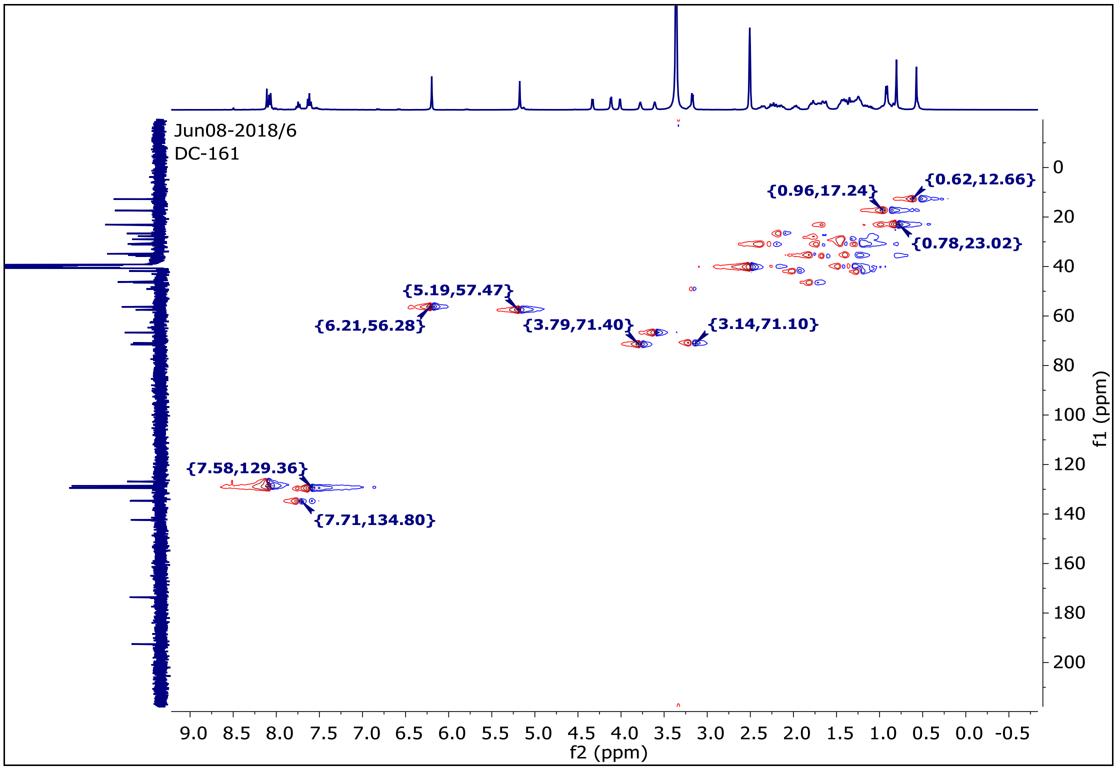

HMBC of 6aa

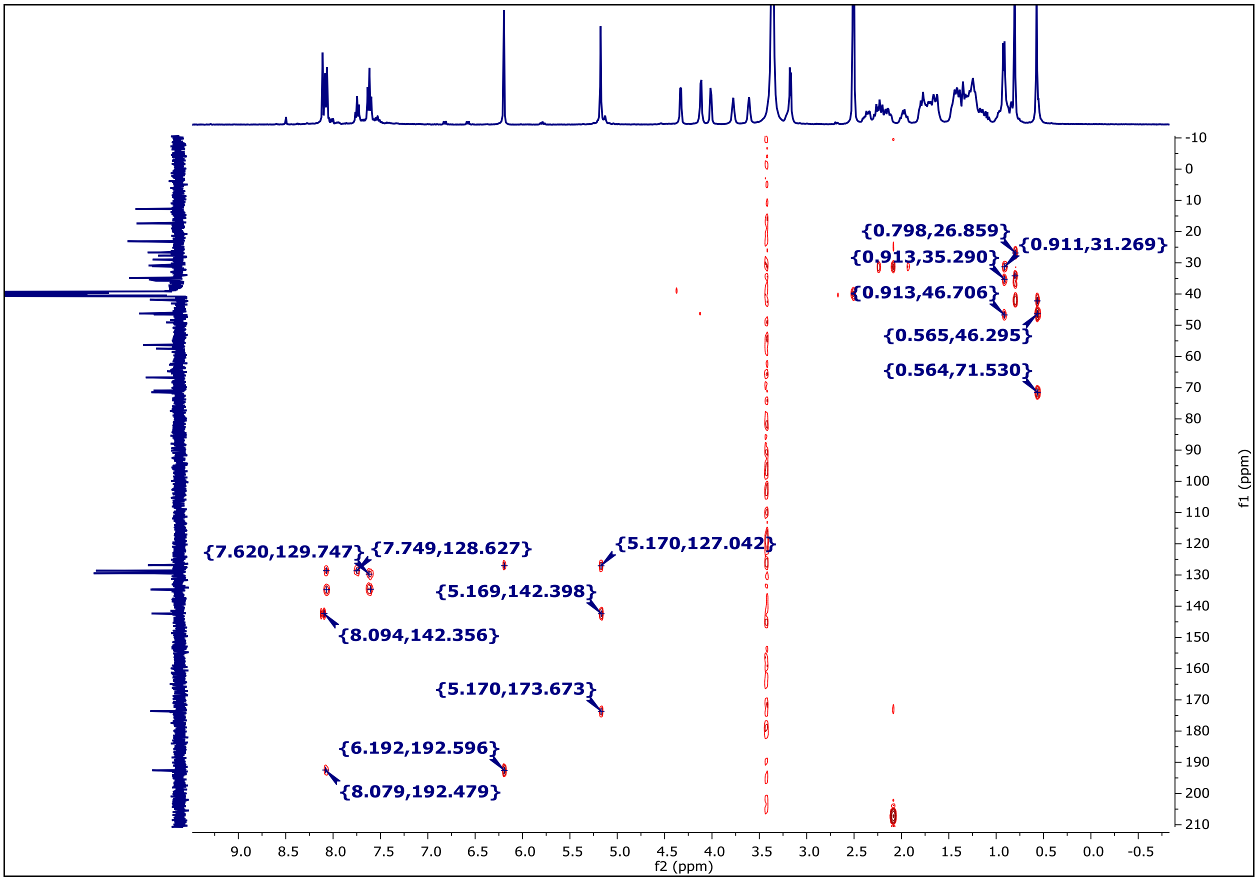

HRMS of 6aa

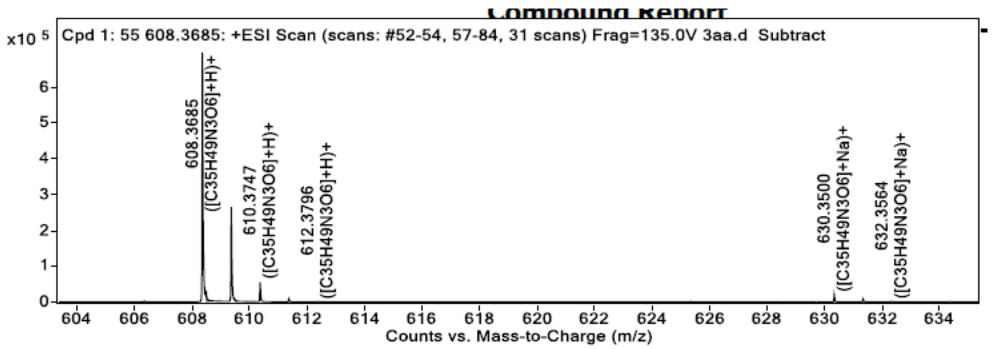

HRMS of 6ba

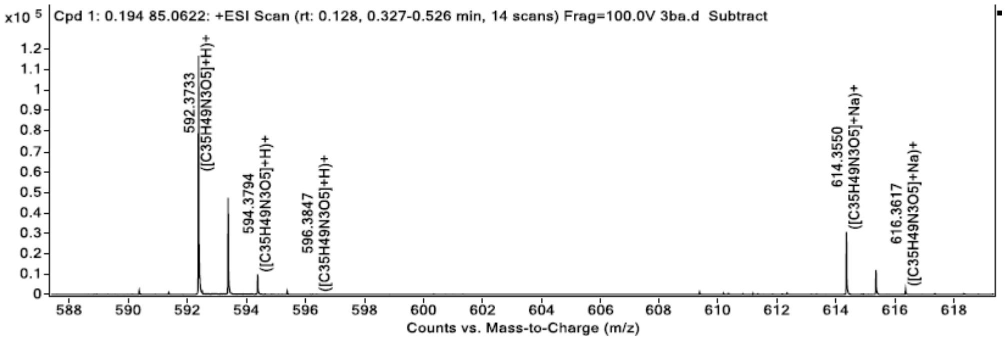

HRMS of 6ca

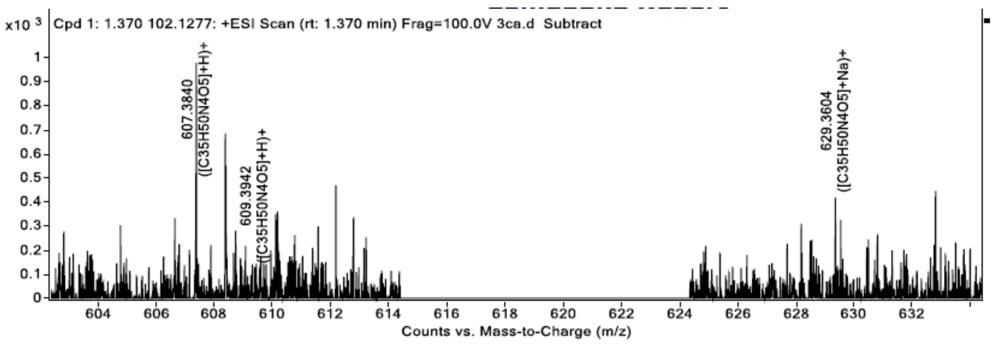

HRMS of 6da

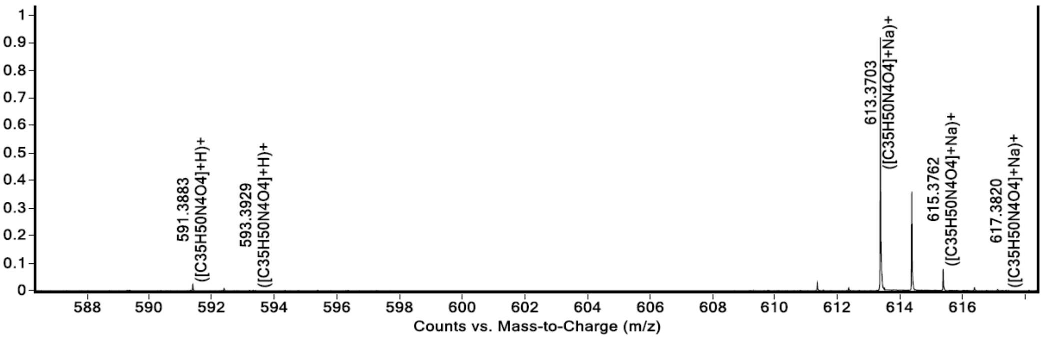

HRMS of 6ab

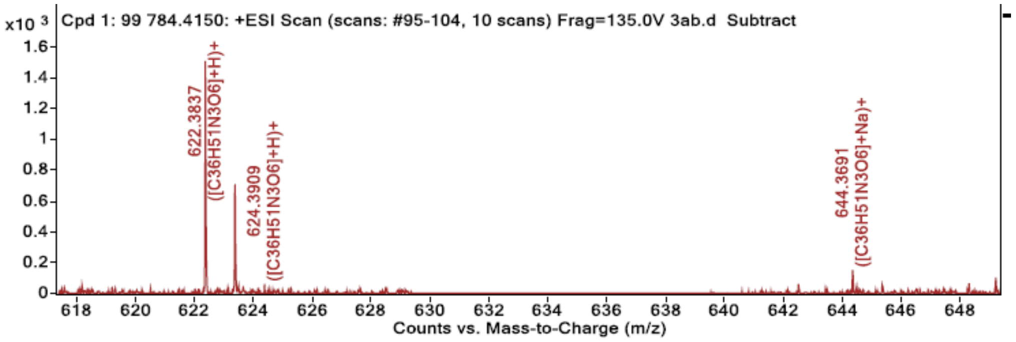

HRMS of 6bb

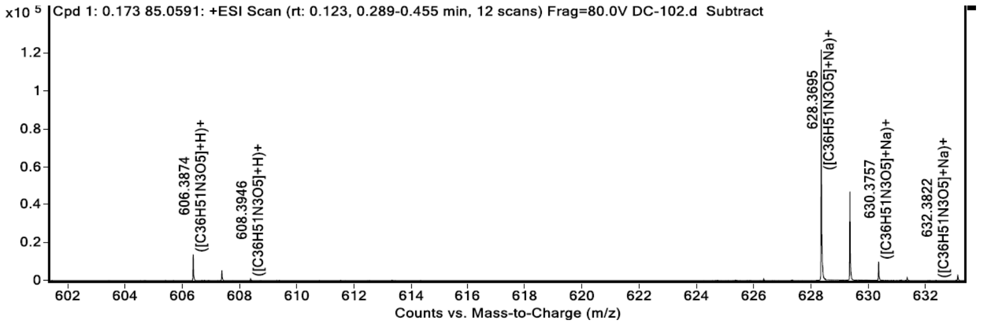

HRMS of 6cb

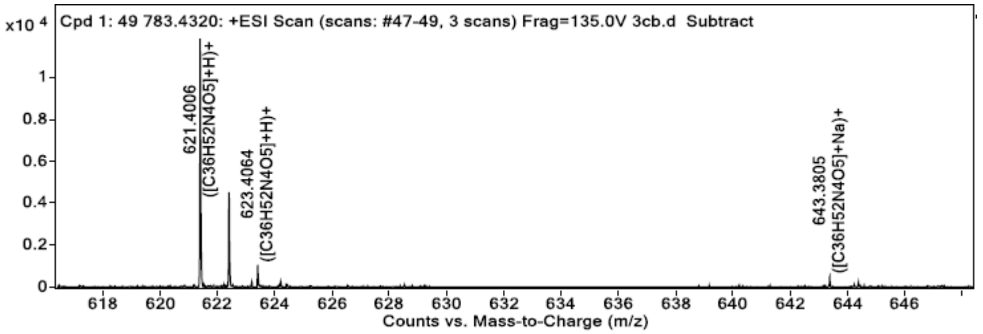

HRMS of 6db

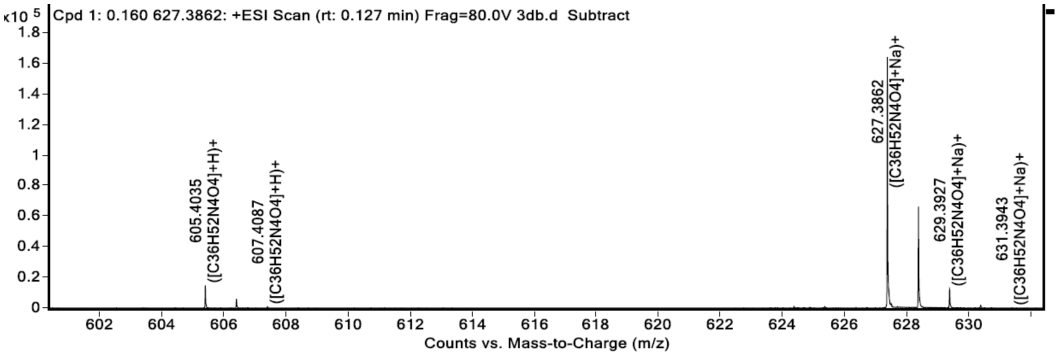

HRMS of 6ac

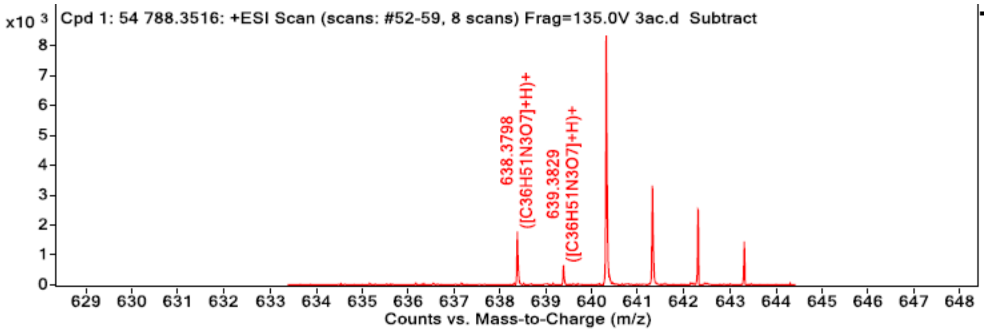

HRMS of 6bc

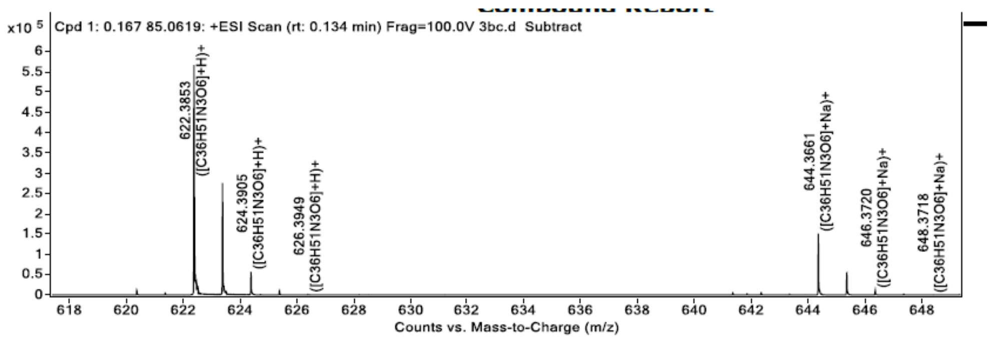

HRMS of 6cc

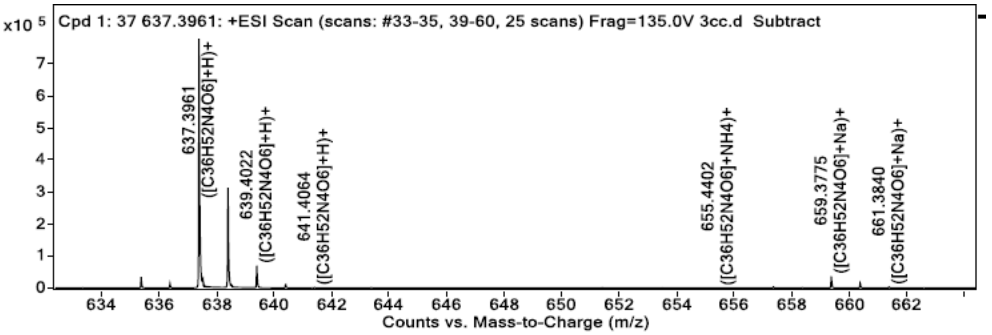

HRMS of 6dc

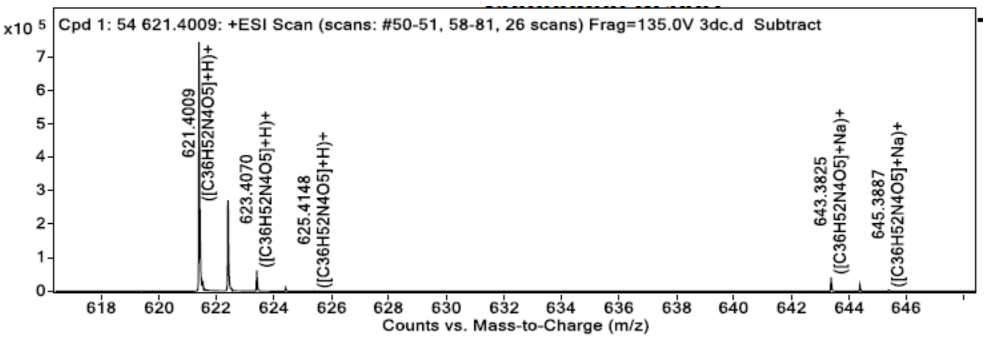

HRMS of 6ad

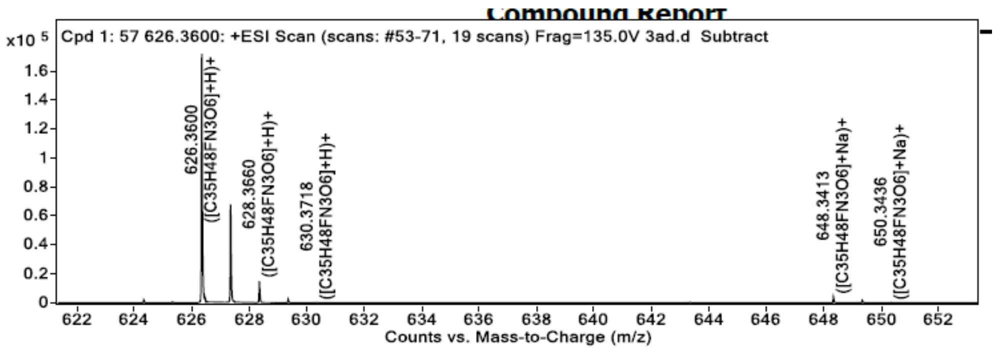

HRMS of 6bd

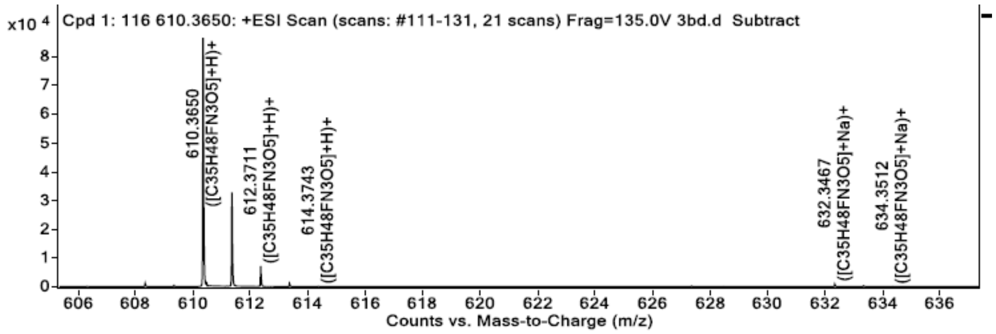

HRMS of 6cd

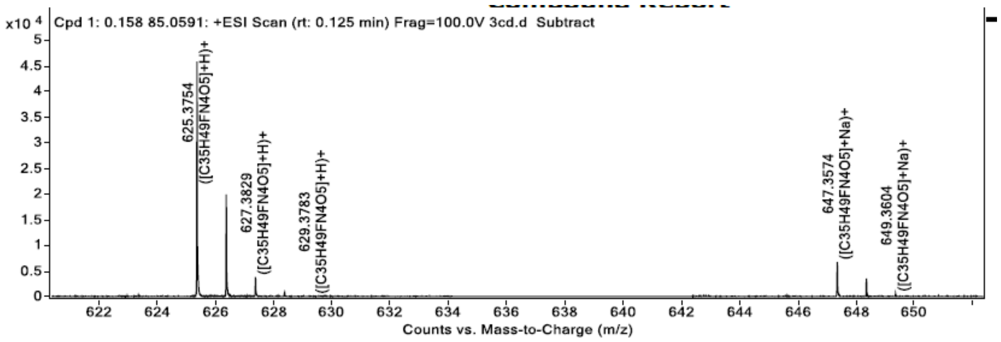

HRMS of 6dd

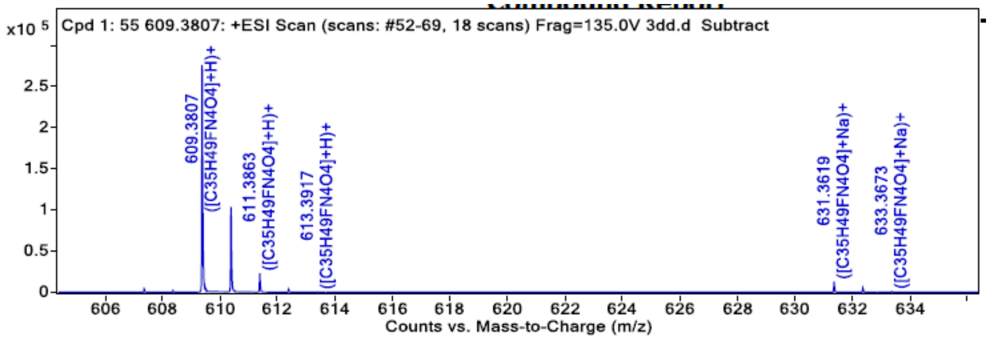

HRMS of 6ae

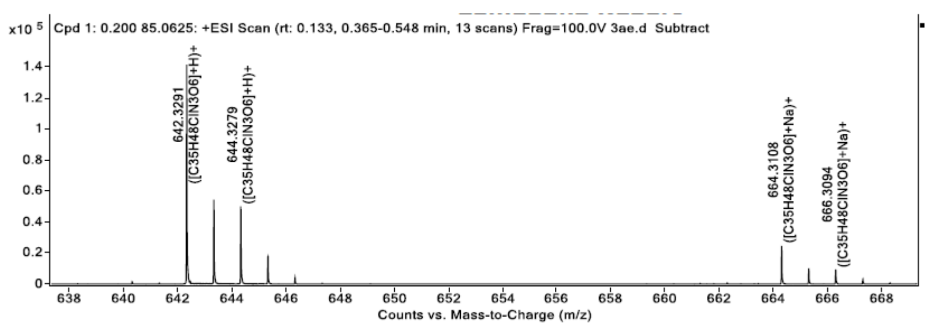

HRMS of 6be

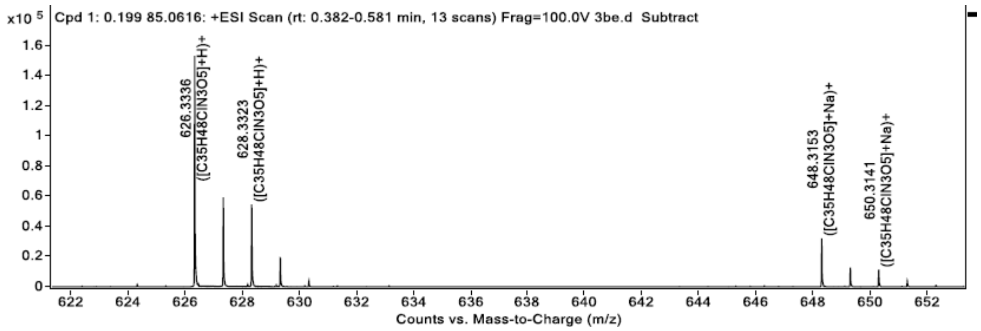

HRMS of 6ce

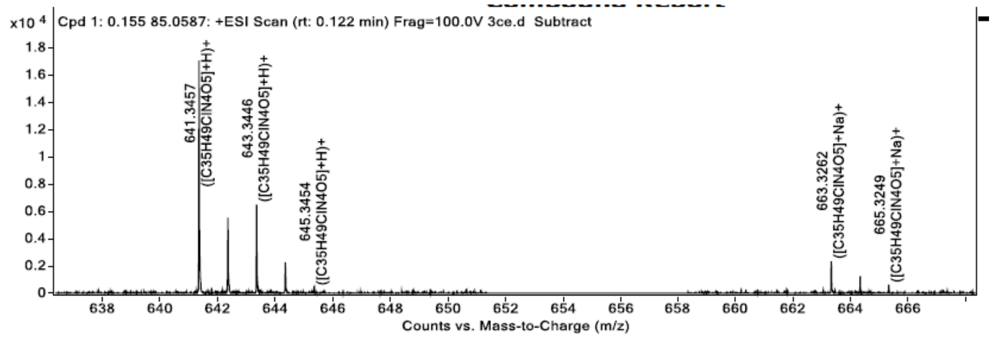

HRMS of 6de

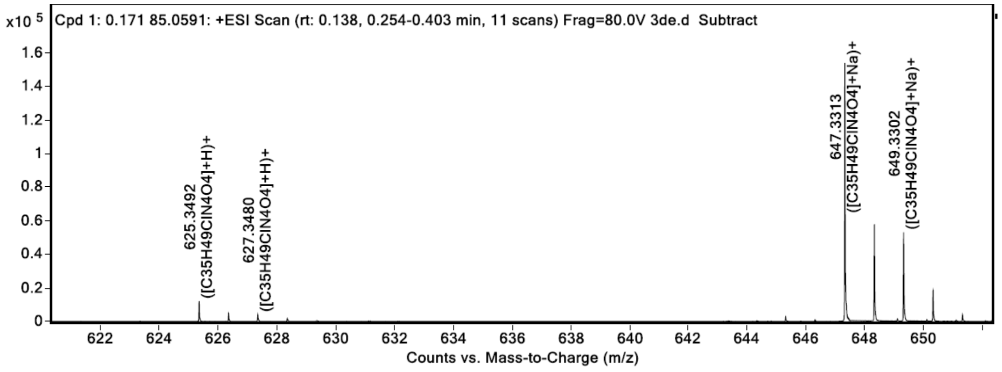

HRMS of 6af

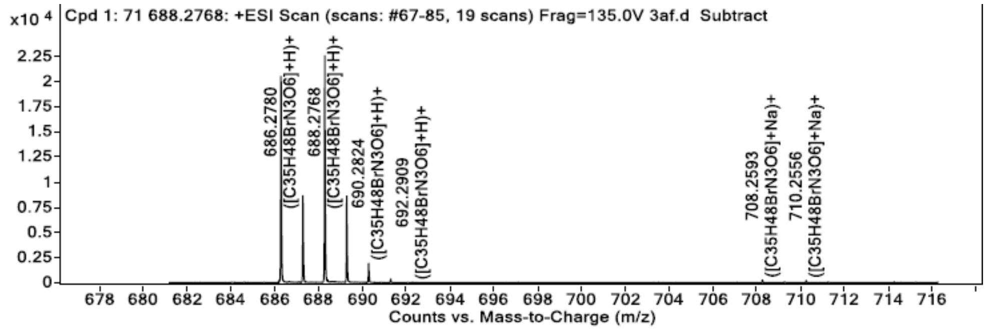

HRMS of 6bf

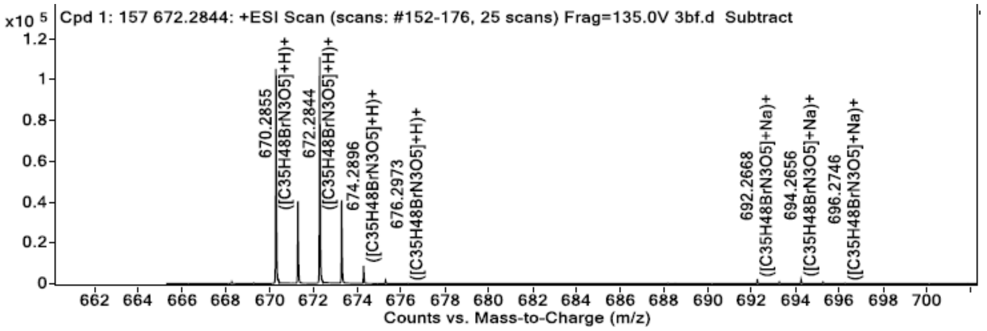

HRMS of 6cf

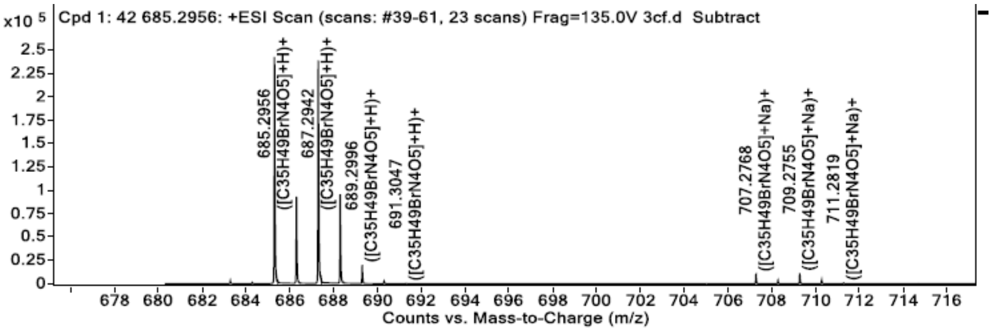

HRMS of 6df

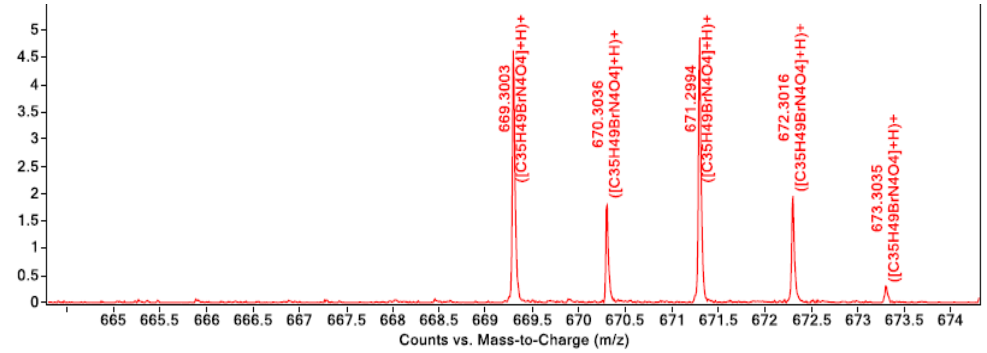

HPLC chromatogram of 6aa

Chromatogram

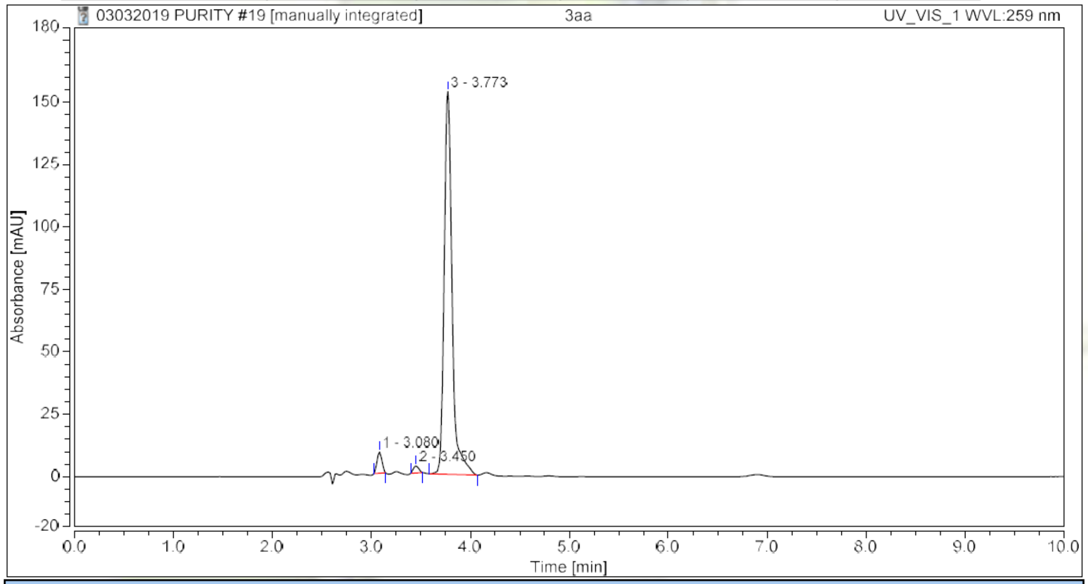

Integration Results

No.	Peak Name	Retention Time min	Area mAU*min	Height mAU	Relative Area %	Relative Height %	Amount n.a.
1		3.080	0.490	8.651	3.32	5.25	n.a.
2		3.450	0.179	2.865	1.22	1.74	n.a.
3		3.773	14.084	153.375	95.46	93.02	n.a.
**Total:**			**14.753**	**164.891**	**100.00**	**100.00**	

HPLC chromatogram of 6ba

Chromatogram

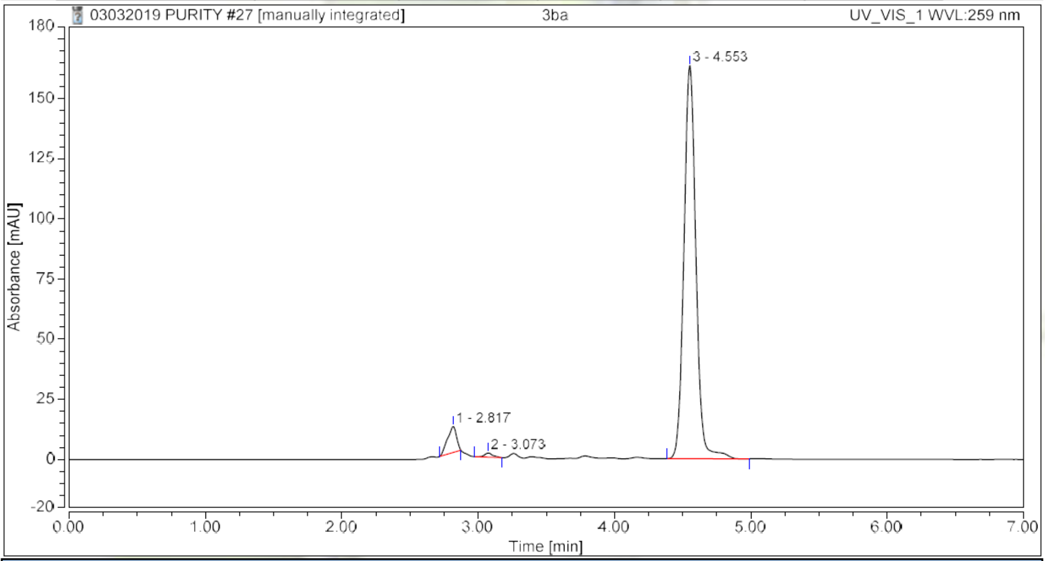

Integration Results

No.	Peak Name	Retention Time min	Area mAU*min	Height mAU	Relative Area %	Relative Height %	Amount n.a.
1		2.817	0.837	10.888	4.75	6.19	n.a.
2		3.073	0.123	1.737	0.70	0.99	n.a.
3		4.553	16.651	163.381	94.55	92.83	n.a.
**Total:**			**17.611**	**176.006**	**100.00**	**100.00**	

HPLC chromatogram of 6ca

Chromatogram

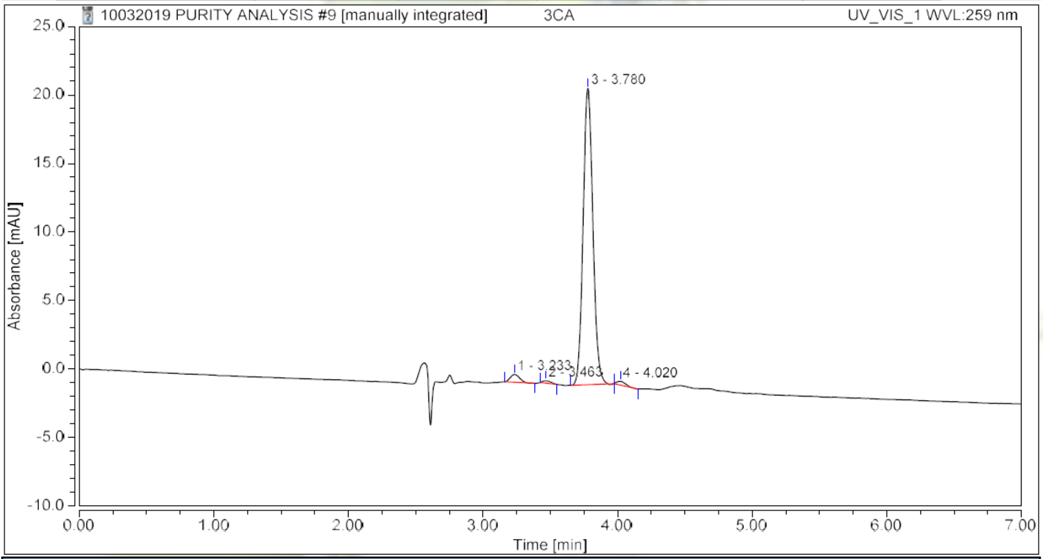

Integration Results

No.	Peak Name	Retention Time min	Area mAU*min	Height mAU	Relative Area %	Relative Height %	Amount n.a.
1		3.233	0.046	0.579	2.45	2.56	n.a.
2		3.463	0.012	0.179	0.65	0.79	n.a.
3		3.780	1.814	21.620	95.86	95.45	n.a.
4		4.020	0.020	0.272	1.04	1.20	n.a.
**Total:**			**1.893**	**22.651**	**100.00**	**100.00**	

HPLC chromatogram of 6da

Chromatogram

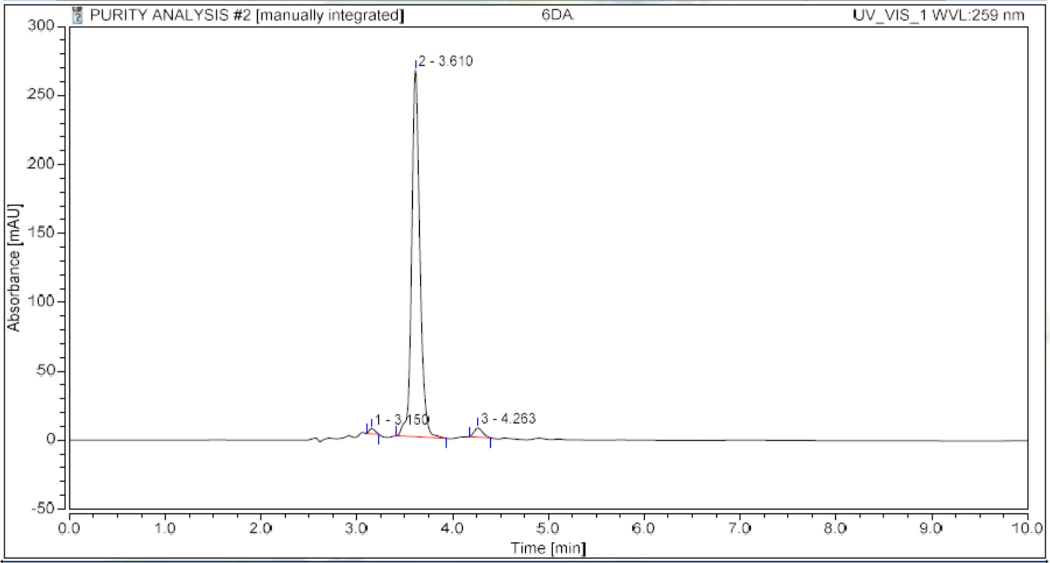

Integration Results

No.	Peak Name	Retention Time min	Area mAU*min	Height mAU	Relative Area %	Relative Height %	Amount n.a.
1		3.150	0.268	3.624	0.97	1.31	n.a.
2		3.610	26.594	265.966	96.50	96.25	n.a.
3		4.263	0.697	6.724	2.53	2.43	n.a.
**Total:**			**27.559**	**276.314**	**100.00**	**100.00**	

HPLC chromatogram of 6ab

Chromatogram

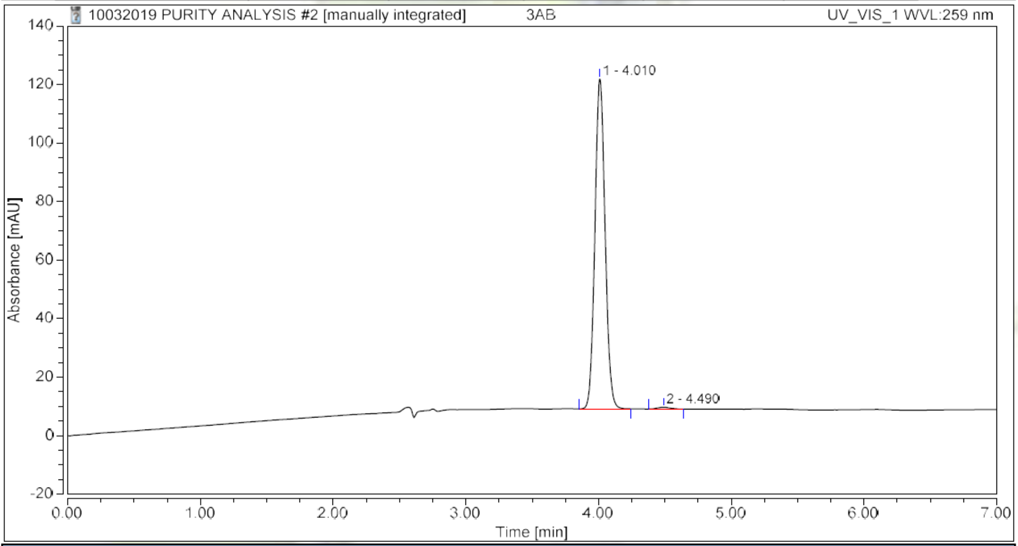

Integration Results

No.	Peak Name	Retention Time min	Area mAU*min	Height mAU	Relative Area %	Relative Height %	Amount n.a.
1		4.010	10.013	112.731	99.37	99.49	n.a.
2		4.490	0.063	0.574	0.63	0.51	n.a.
**Total:**			**10.077**	**113.305**	**100.00**	**100.00**	

HPLC chromatogram of 6bb

Chromatogram

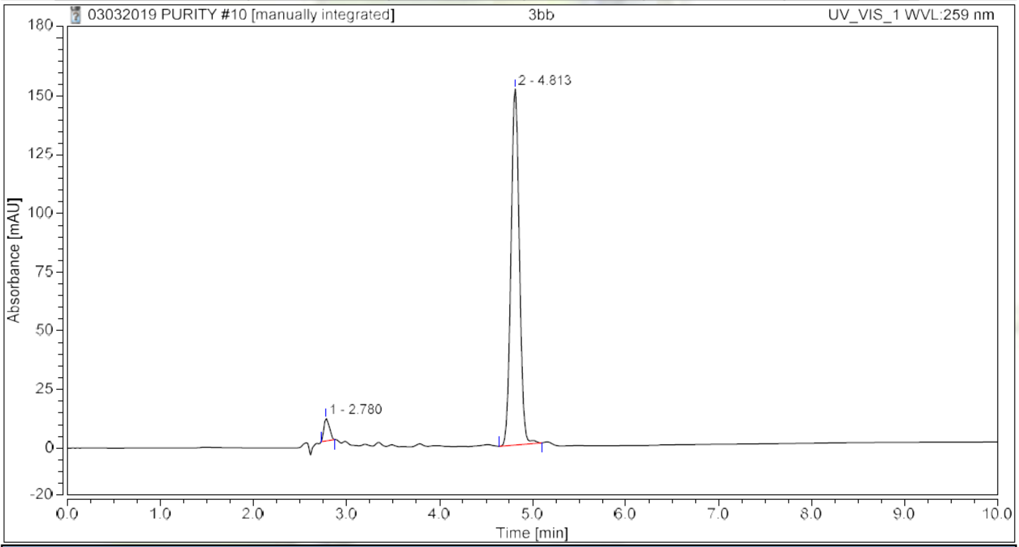

Integration Results

No.	Peak Name	Retention Time min	Area mAU*min	Height mAU	Relative Area %	Relative Height %	Amount n.a.
1		2.780	0.665	9.644	4.01	5.98	n.a.
2		4.813	15.920	151.748	95.99	94.02	n.a.
**Total:**			**16.585**	**161.392**	**100.00**	**100.00**	

HPLC chromatogram of 6cb

Chromatogram

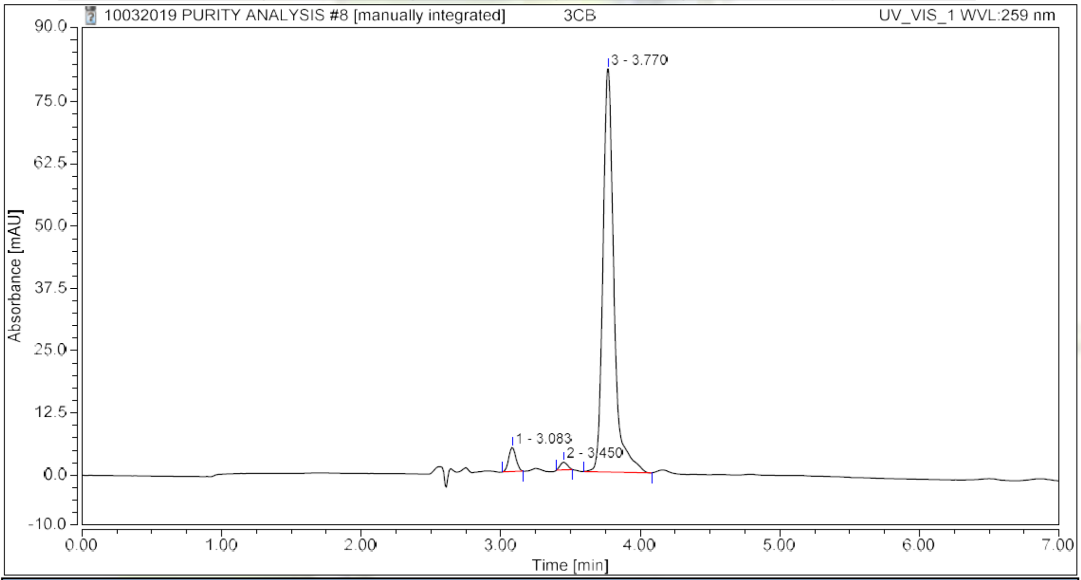

Integration Results

No.	Peak Name	Retention Time min	Area mAU*min	Height mAU	Relative Area %	Relative Height %	Amount n.a.
1		3.083	0.289	4.835	3.73	5.53	n.a.
2		3.450	0.094	1.543	1.21	1.77	n.a.
3		3.770	7.372	80.972	95.06	92.70	n.a.
**Total:**			**7.756**	**87.350**	**100.00**	**100.00**	

HPLC chromatogram of 6db

Chromatogram

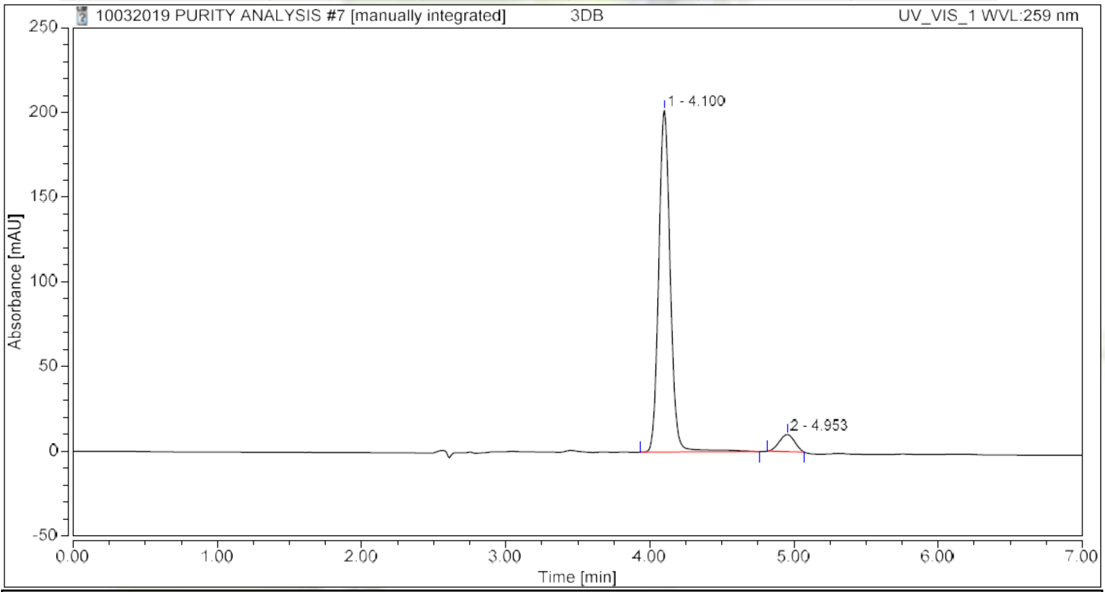

Integration Results

No.	Peak Name	Retention Time min	Area mAU*min	Height mAU	Relative Area %	Relative Height %	Amount n.a.
1		4.100	18.801	201.645	93.82	95.24	n.a.
2		4.953	1.239	10.086	6.18	4.76	n.a.
**Total:**			**20.040**	**211.731**	**100.00**	**100.00**	

HPLC chromatogram of 6ac

Chromatogram

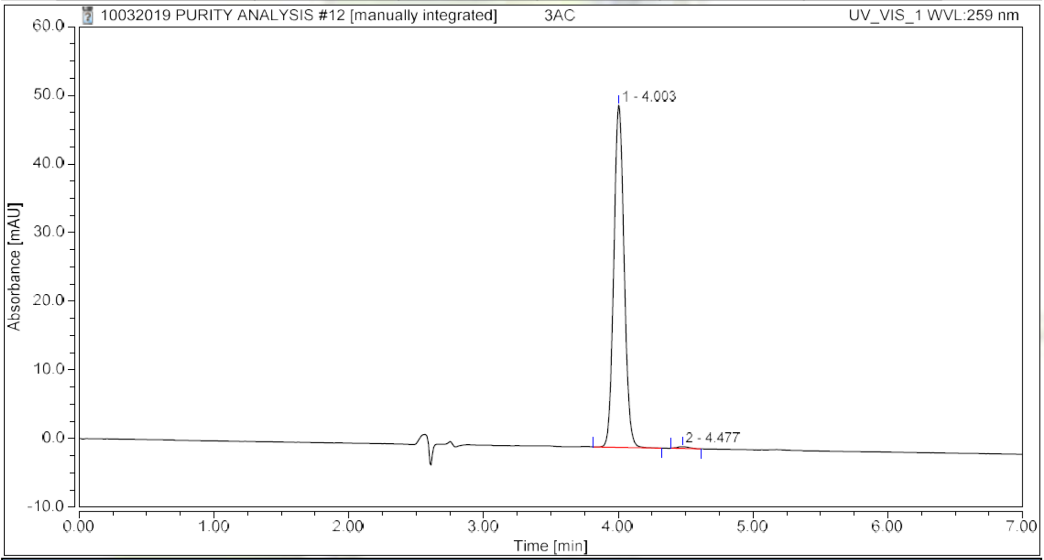

Integration Results

No.	Peak Name	Retention Time min	Area mAU*min	Height mAU	Relative Area %	Relative Height %	Amount n.a.
1		4.003	4.402	49.846	99.43	99.48	n.a.
2		4.477	0.025	0.260	0.57	0.52	n.a.
**Total:**			**4.428**	**50.106**	**100.00**	**100.00**	

HPLC chromatogram of 6bc

Chromatogram

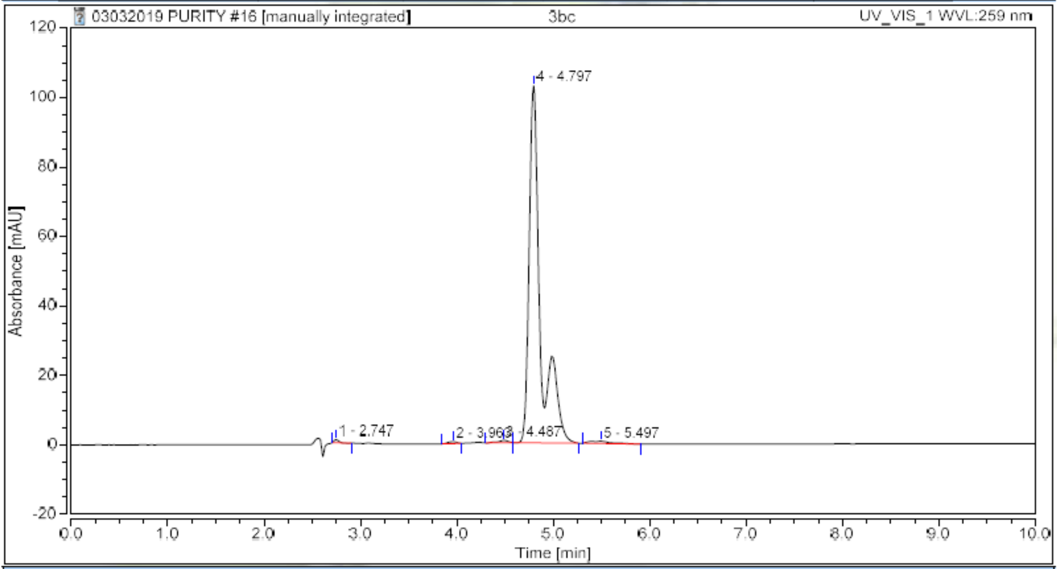

Integration Results

No.	Peak Name	Retention Time min	Area mAU*min	Height mAU	Relative Area %	Relative Height %	Amount n.a.
1		2.747	0.047	0.879	0.33	0.83	n.a.
2		3.963	0.059	0.563	0.41	0.53	n.a.
3		4.487	0.079	0.674	0.55	0.64	n.a.
4		4.797	14.069	102.808	97.61	97.40	n.a.
5		5.497	0.159	0.627	1.10	0.59	n.a.
**Total:**			**14.414**	**105.551**	**100.00**	**100.00**	

HPLC chromatogram of 6cc

Chromatogram

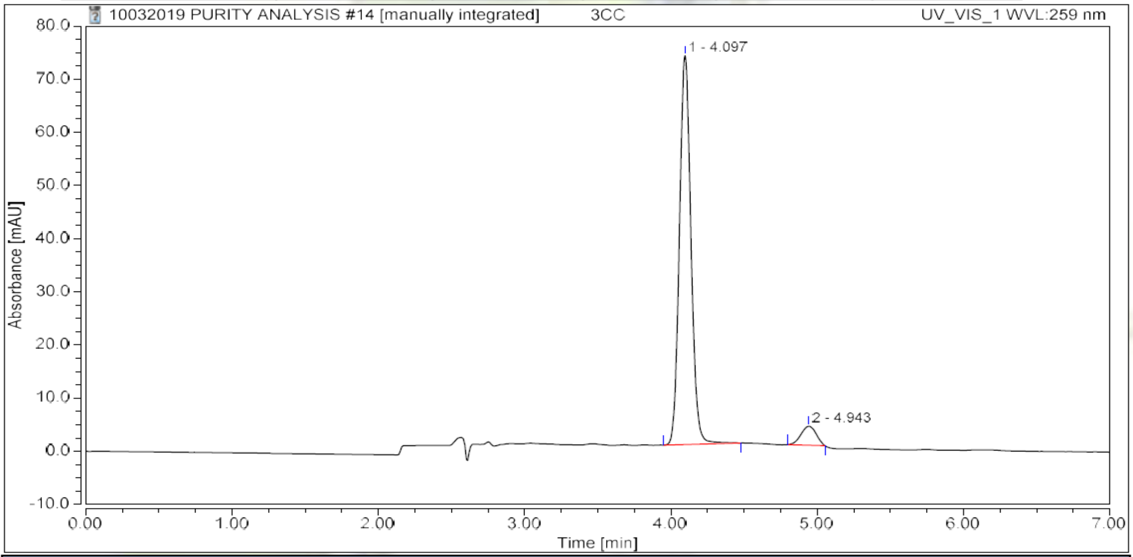

Integration Results

No.	Peak Name	Retention Time min	Area mAU*min	Height mAU	Relative Area %	Relative Height %	Amount n.a.
1		4.097	6.669	73.152	93.92	95.33	n.a.
2		4.943	0.432	3.587	6.08	4.67	n.a.
**Total:**			**7.101**	**76.739**	**100.00**	**100.00**	

HPLC chromatogram of 6dc

Chromatogram

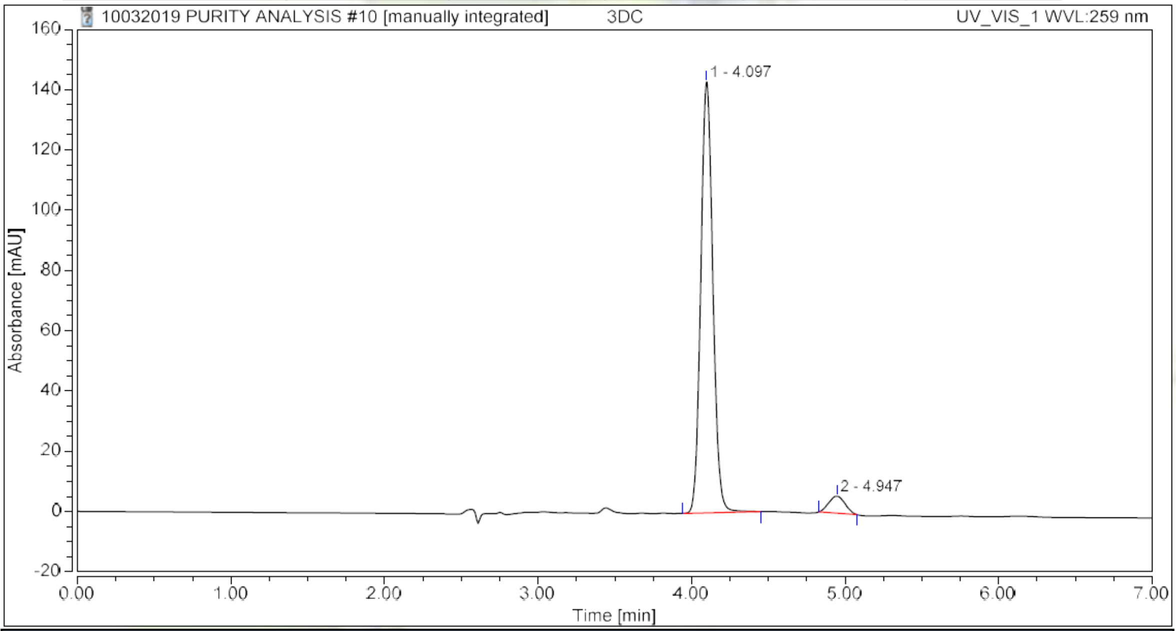

Integration Results

No.	Peak Name	Retention Time min	Area mAU*min	Height mAU	Relative Area %	Relative Height %	Amount n.a.
1		4.097	13.014	143.123	94.90	96.17	n.a.
2		4.947	0.699	5.694	5.10	3.83	n.a.
**Total:**			**13.713**	**148.817**	**100.00**	**100.00**	

HPLC chromatogram of 6ad

Chromatogram

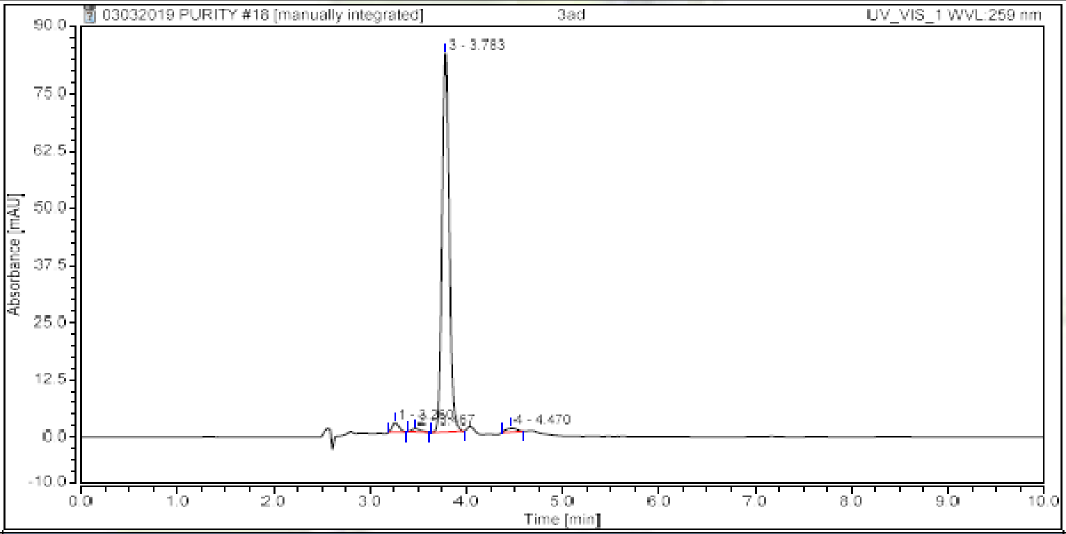

Integration Results

No.	Peak Name	Retention Time<br>min	Area<br>mAU*min	Height<br>mAU	Relative Area<br>%	Relative Height<br>%	Amount<br>n.a.
1		3.260	0.166	2.069	2.24	2.39	n.a.
2		3.467	0.080	0.750	1.08	0.87	n.a.
3		3.783	7.053	82.872	95.12	95.74	n.a.
4		4.470	0.116	0.870	1.56	1.01	n.a.
Total:			7.415	86.562	100.00	100.00	

HPLC chromatogram of 6bd

Chromatogram

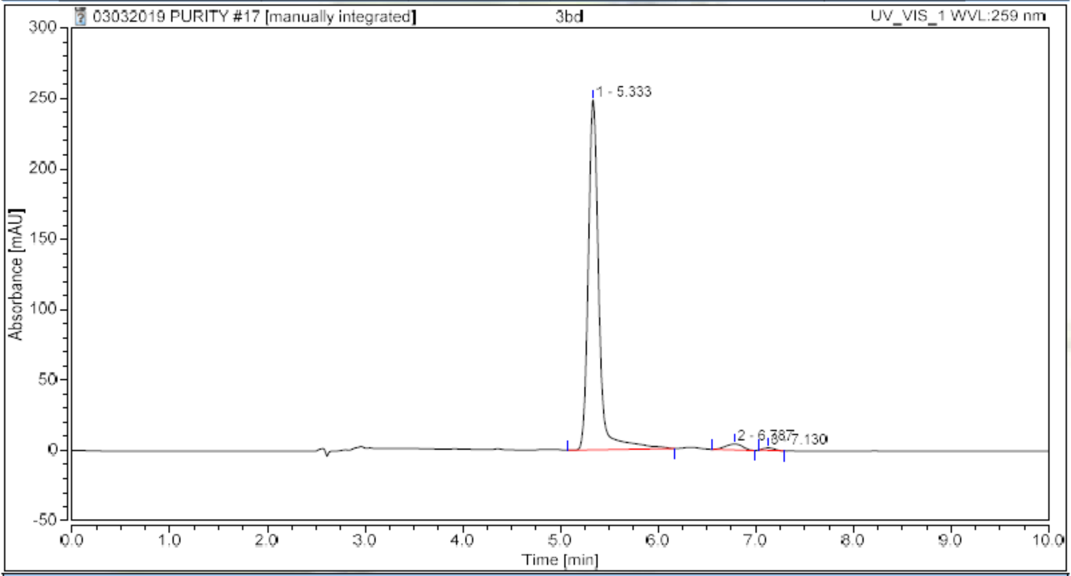

Integration Results

No.	Peak Name	Retention Time<br>min	Area<br>mAU*min	Height<br>mAU	Relative Area<br>%	Relative Height<br>%	Amount<br>n.a.
1		5.333	31.413	248.420	96.72	97.66	n.a.
2		6.787	0.835	4.138	2.57	1.63	n.a.
3		7.130	0.232	1.805	0.71	0.71	n.a.
Total:			32.480	254.363	100.00	100.00	

HPLC chromatogram of 6cd

Chromatogram

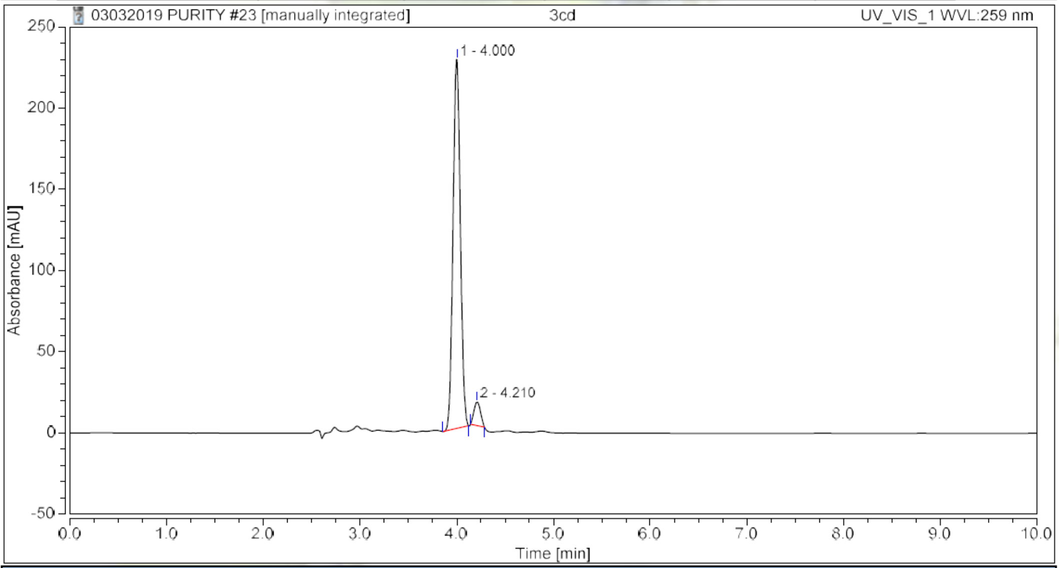

Integration Results

No.	Peak Name	Retention Time min	Area mAU*min	Height mAU	Relative Area %	Relative Height %	Amount n.a.
1		4.000	19.930	227.234	94.54	93.91	n.a.
2		4.210	1.152	14.747	5.46	6.09	n.a.
**Total:**			**21.082**	**241.981**	**100.00**	**100.00**	

HPLC chromatogram of 6dd

Chromatogram

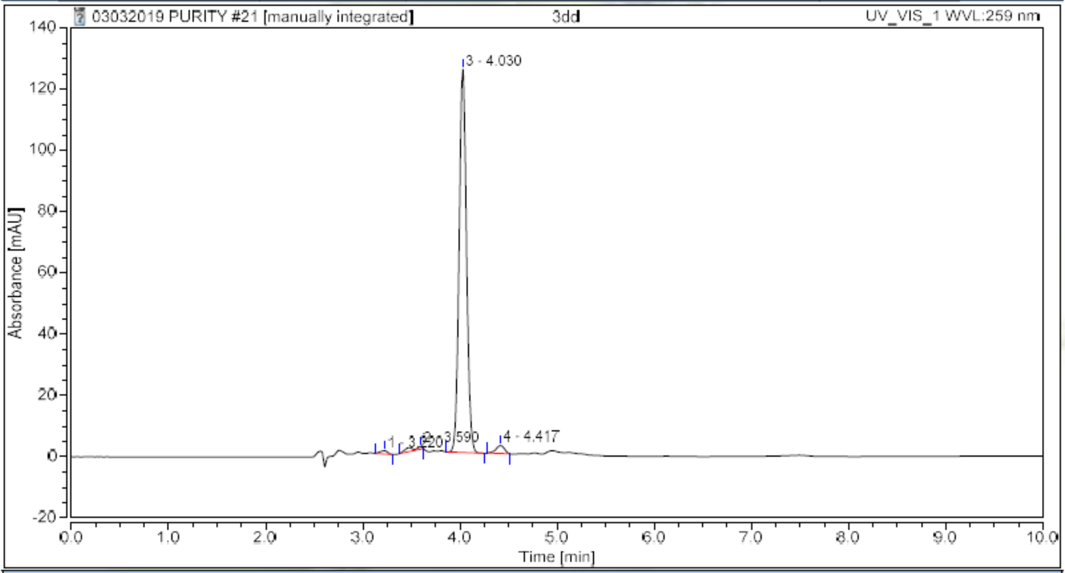

Integration Results

No.	Peak Name	Retention Time min	Area mAU*min	Height mAU	Relative Area %	Relative Height %	Amount n.a.
1		3.220	0.094	1.064	0.81	0.82	n.a.
2		3.590	0.165	0.876	1.42	0.67	n.a.
3		4.030	11.092	125.218	95.47	96.45	n.a.
4		4.417	0.268	2.666	2.30	2.05	n.a.
**Total:**			**11.618**	**129.823**	**100.00**	**100.00**	

HPLC chromatogram of 6ae

Chromatogram

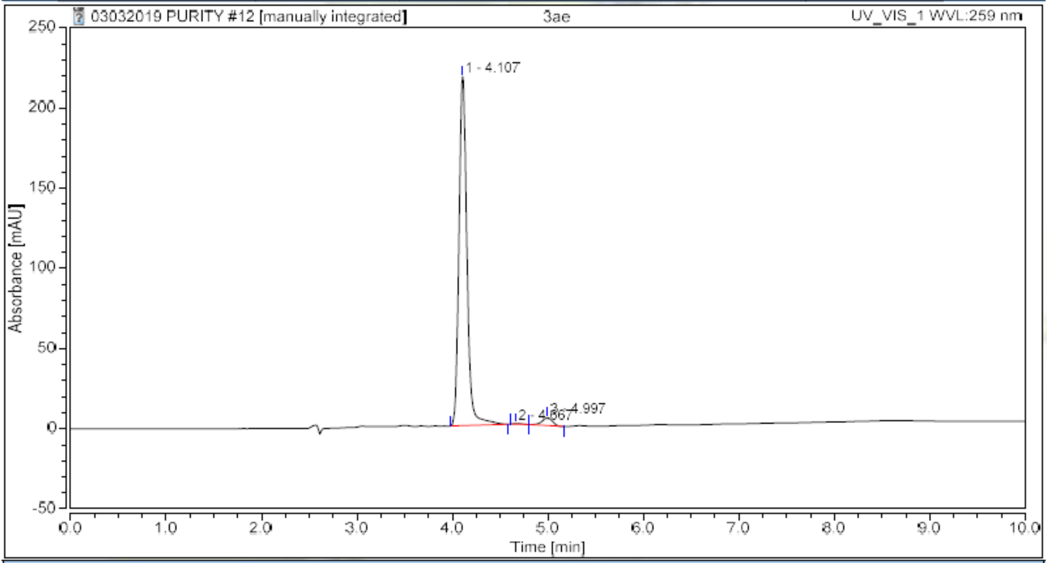

Integration Results

No.	Peak Name	Retention Time min	Area mAU*min	Height mAU	Relative Area %	Relative Height %	Amount n.a.
1		4.107	21.009	217.661	97.01	97.60	n.a.
2		4.667	0.045	0.460	0.21	0.21	n.a.
3		4.997	0.601	4.901	2.78	2.20	n.a.
**Total:**			**21.656**	**223.022**	**100.00**	**100.00**	

HPLC chromatogram of 6be

Chromatogram

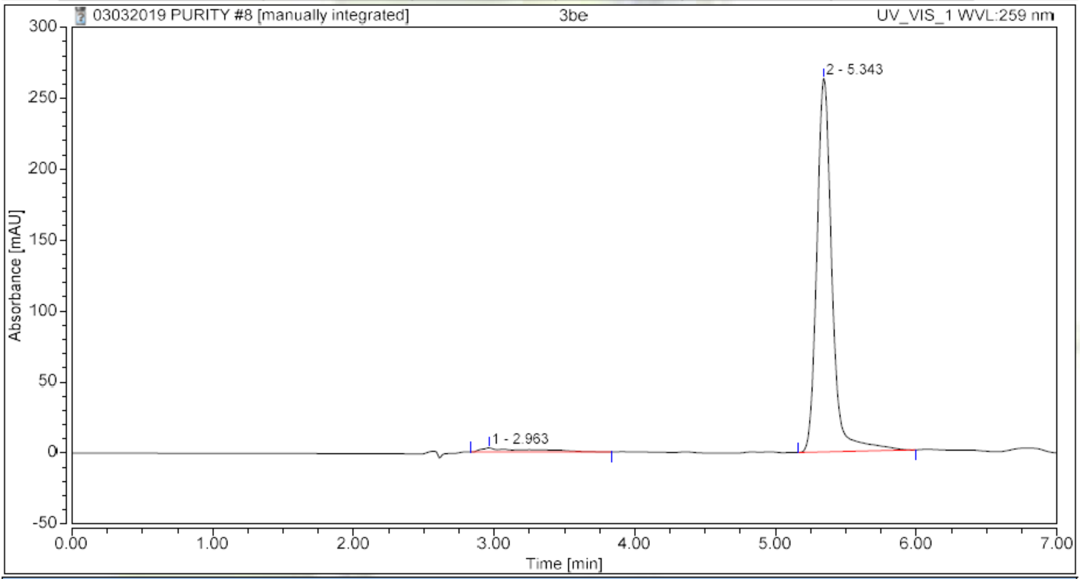

Integration Results

No.	Peak Name	Retention Time min	Area mAU*min	Height mAU	Relative Area %	Relative Height %	Amount n.a.
1		2.963	1.111	2.864	3.27	1.08	n.a.
2		5.343	32.871	263.294	96.73	98.92	n.a.
**Total:**			**33.983**	**266.158**	**100.00**	**100.00**	

HPLC chromatogram 6ce

Chromatogram

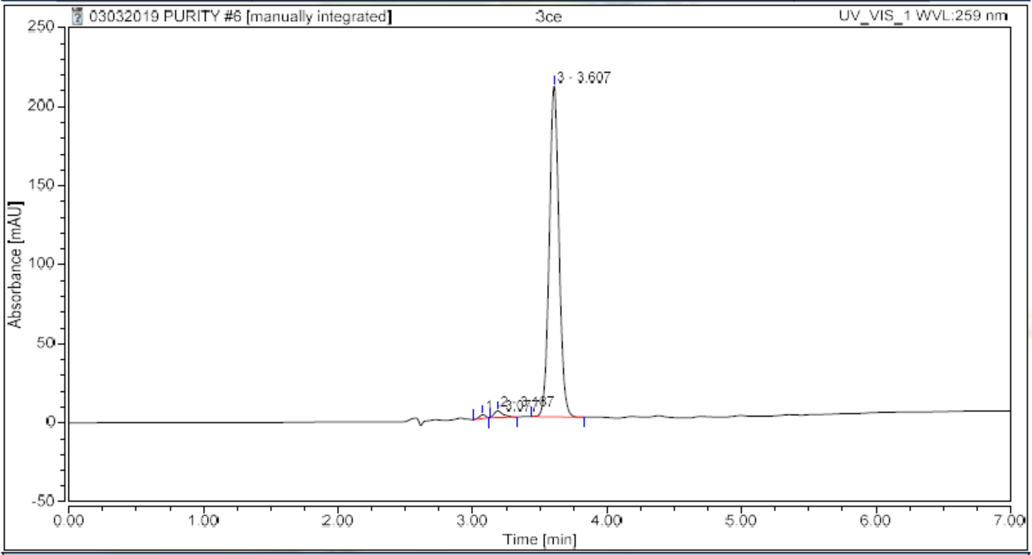

Integration Results

No.	Peak Name	Retention Time min	Area mAU*min	Height mAU	Relative Area %	Relative Height %	Amount n.a.
1		3.077	0.121	2.259	0.67	1.05	n.a.
2		3.187	0.315	4.178	1.76	1.94	n.a.
3		3.607	17.529	209.082	97.57	97.01	n.a.
Total:			**17.965**	**215.519**	**100.00**	**100.00**	

HPLC chromatogram of 6de

Chromatogram

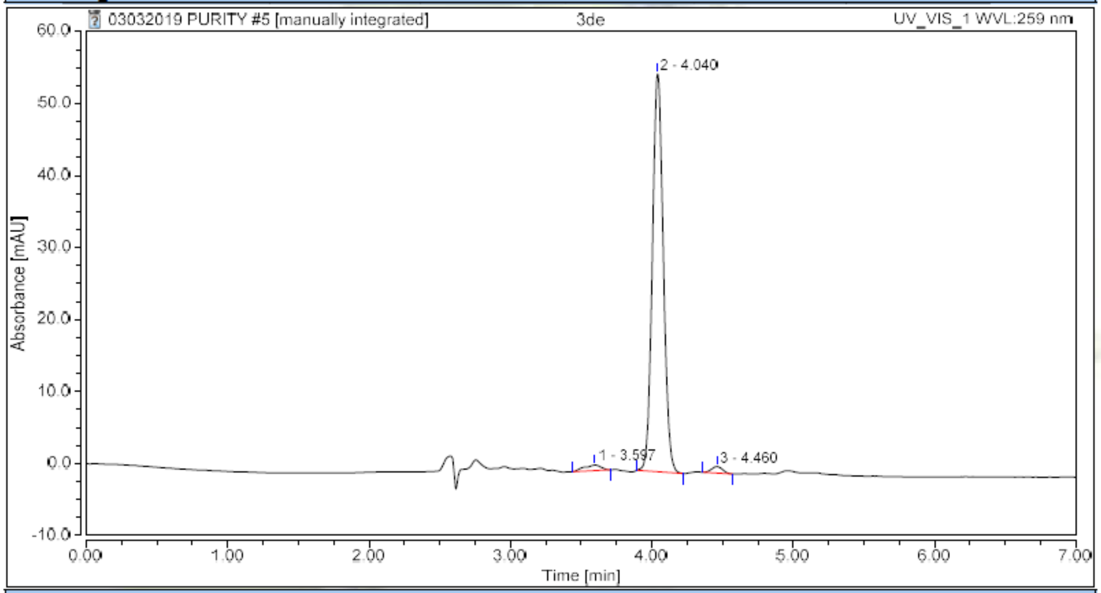

Integration Results

No.	Peak Name	Retention Time min	Area mAU*min	Height mAU	Relative Area %	Relative Height %	Amount n.a.
1		3.597	0.108	0.782	2.10	1.37	n.a.
2		4.040	4.954	55.233	96.32	97.03	n.a.
3		4.460	0.081	0.907	1.58	1.59	n.a.
Total:			**5.143**	**56.922**	**100.00**	**100.00**	

HPLC chromatogram of 6af

Chromatogram

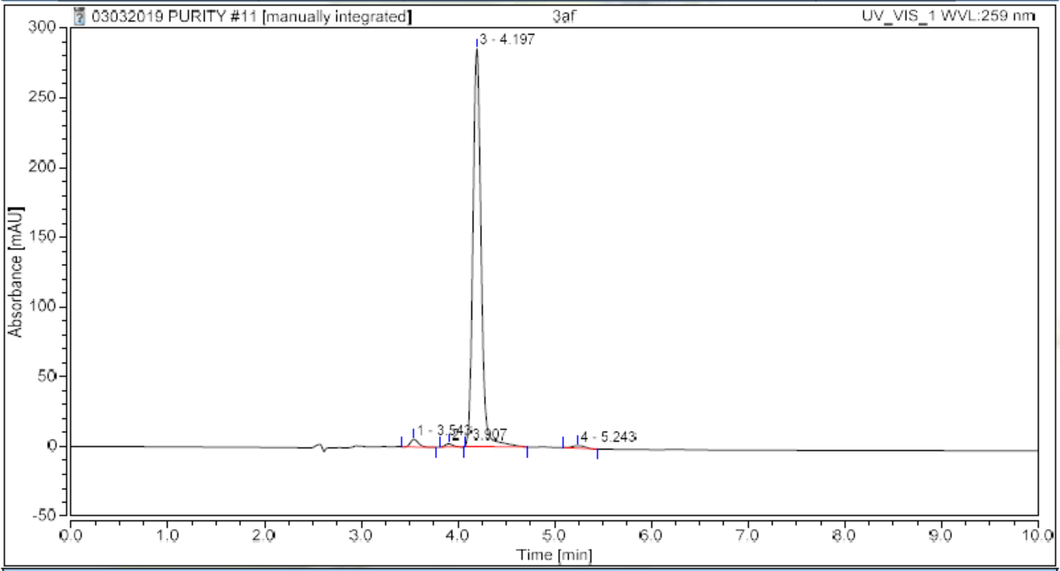

Integration Results

No.	Peak Name	Retention Time min	Area mAU*min	Height mAU	Relative Area %	Relative Height %	Amount n.a.
1		3.543	0.512	5.509	1.78	1.87	n.a.
2		3.907	0.201	2.377	0.70	0.81	n.a.
3		4.197	27.758	285.187	96.62	96.72	n.a.
4		5.243	0.259	1.784	0.90	0.60	n.a.
Total:			**28.730**	**294.857**	**100.00**	**100.00**	

HPLC chromatogram of 6bf

Chromatogram

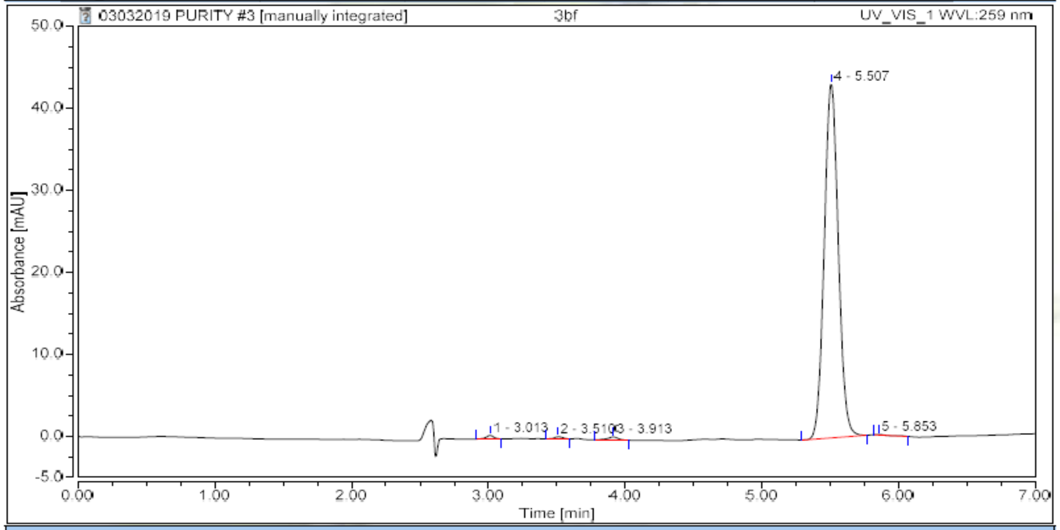

Integration Results

No.	Peak Name	Retention Time min	Area mAU*min	Height mAU	Relative Area %	Relative Height %	Amount n.a.
1		3.013	0.026	0.415	0.50	0.94	n.a.
2		3.510	0.018	0.255	0.34	0.57	n.a.
3		3.913	0.034	0.379	0.65	0.86	n.a.
4		5.507	5.142	43.156	98.37	97.48	n.a.
5		5.853	0.007	0.069	0.14	0.16	n.a.
Total:			**5.227**	**44.273**	**100.00**	**100.00**	

HPLC chromatogram of 6cf

Chromatogram

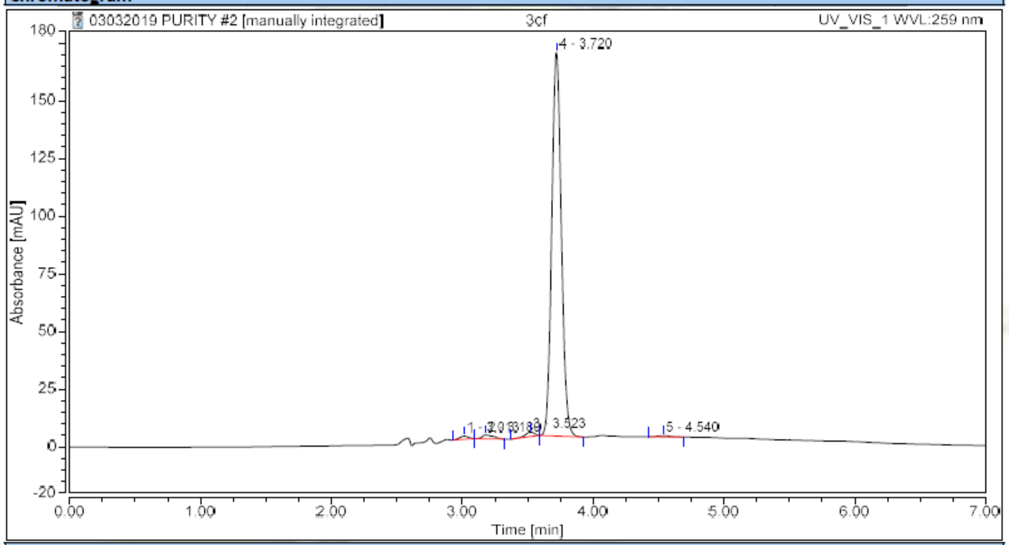

Integration Results

No.	Peak Name	Retention Time min	Area mAU*min	Height mAU	Relative Area %	Relative Height %	Amount n.a.
1		3.013	0.101	1.363	0.69	0.79	n.a.
2		3.180	0.164	1.642	1.12	0.96	n.a.
3		3.523	0.150	1.892	1.03	1.10	n.a.
4		3.720	14.070	166.164	96.70	96.81	n.a.
5		4.540	0.066	0.570	0.45	0.33	n.a.
**Total:**			**14.550**	**171.632**	**100.00**	**100.00**	

HPLC chromatogram of 6df

Chromatogram

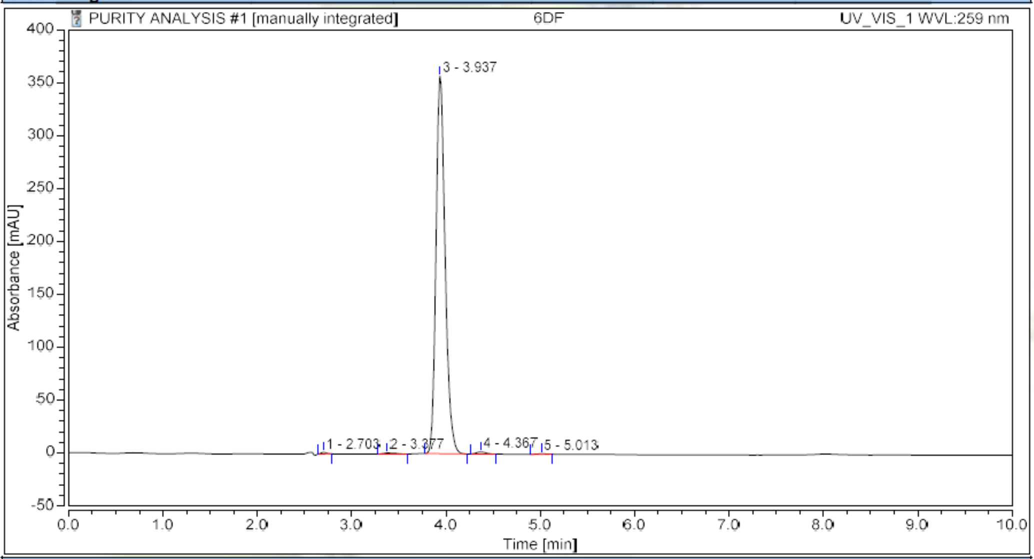

Integration Results

No.	Peak Name	Retention Time min	Area mAU*min	Height mAU	Relative Area %	Relative Height %	Amount n.a.
1		2.703	0.105	1.573	0.26	0.43	n.a.
2		3.377	0.140	0.935	0.35	0.26	n.a.
3		3.937	39.949	357.101	98.65	98.62	n.a.
4		4.367	0.242	1.989	0.60	0.55	n.a.
5		5.013	0.059	0.514	0.15	0.14	n.a.
**Total:**			**40.495**	**362.113**	**100.00**	**100.00**	
